# Base-Mediated Synthesis of Imidazole-Fused 1,4-Benzoxazepines via 7-*exo-dig* Cyclizations: Propargyl Group Transformation

**DOI:** 10.1021/acs.joc.5c00106

**Published:** 2025-03-26

**Authors:** Nalan Korkmaz Cokol, Fevzi Can Inyurt, İpek Öktem, Ertan Sahin, Ozlem Sari, Cagatay Dengiz, Metin Balci

**Affiliations:** † Department of Chemistry, Middle East Technical University, 06800 Ankara, Turkey; ‡ Department of Chemistry, Atatürk University, 25240 Erzurum, Turkey; § Network Technologies Department, TÜBİTAK ULAKBİM, TR-06800 Ankara, Turkey

## Abstract

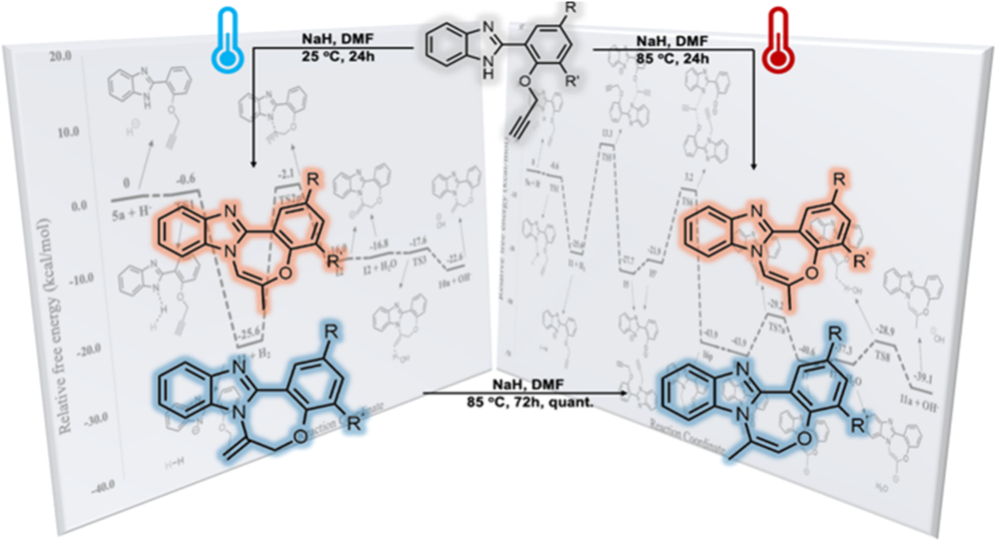

Herein, we describe the synthesis of a series of imidazole-fused 1,4-benzoxazepines using 7-*exo-dig* cyclizations. Two sets of substrates, one containing disubstituted alkyne functional groups and the other featuring terminal alkynes, were synthesized by using *O*-propargylation, Sonogashira cross-coupling, and condensation reactions between aldehydes and *o*-diaminobenzene. While the disubstituted substrates yielded exocyclic *E*/*Z* configured cyclization products smoothly, the reactions involving terminal alkynes resulted in the formation of isomeric products with altered skeletal structures, in addition to the expected 7-*exo-dig* cyclization products. Density functional theory (DFT) calculations were used to clarify the mechanisms underlying the formation of these products. It is suggested that these unexpected products are formed through a series of intermolecular *O*-to-*N*-propargyl transfer reactions, followed by 7-*exo-dig* cyclization, in accordance with Baldwin’s rules. Furthermore, this study extensively demonstrates the conversion of exocyclic products to endocyclic products through a base-mediated 1,3-H shift.

## Introduction

Fused ring systems, characterized by sharing atoms from two or more adjacent rings, are of particular interest due to their diverse biological activities.^1^ Medium-sized seven-membered ring systems hold particular significance because of their higher affinity for biological receptors, improved oral bioavailability, and enhanced cell permeability compared to smaller and larger ring systems.^2^ Among compounds containing a seven-membered fused ring system, benzoxazepines stand out as one of the most notable groups of compounds due to their diverse bioactivity profiles.^3^

**Figure 1:**
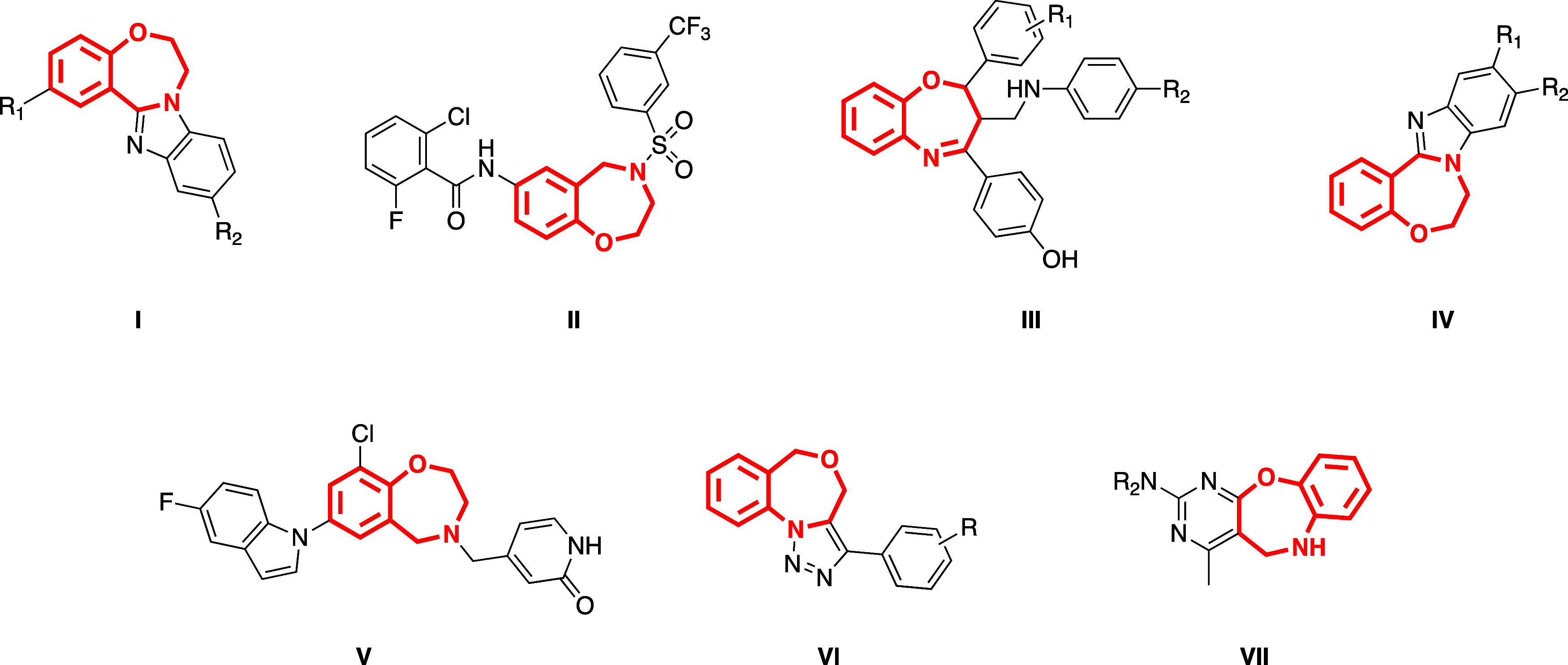
Representative bioactive benzoxazepine derivatives from the literature.

Upon analyzing all potential isomers of benzoxazepines, it appears that 1,4- and 1,5-benzoxazepines emerge as predominant, exhibiting significant biological activities.^4^Figure 1 provides an overview of important examples of biologically active compounds with benzoxazepine core structures. Benzimidazole-fused 1,4-benzoxazepine derivatives **I** have been identified as selective inhibitors of PI3Kα, showing promise for treating tumors.^5^ Moreover, compound **II** exhibited characteristics of a partial inverse agonist for RORγ, while compound **III** underwent assessment as an anticonvulsant agent.^6,7^ It is reported that compound **IV** holds promise as a potential chemotherapeutic agent based on the results from anticancer, anti-inflammatory, and antimicrobial assays.^8^ Additionally, compounds **V**, **VI**, and **VII** exhibit EP2 antagonist properties,^9^ serve as anticancer agents,^10^ and demonstrate antidepressant effects,^11^ respectively. Due to these factors, researchers have been exploring novel synthetic approaches to build synthetic libraries of benzoxazepine derivatives. One of the earliest examples of benzoxazepine synthesis involves the cyclization reactions of aminophthalides in polyphosphoric acid, which are obtained by condensation reactions between 2-aryloxyalkylamines and 2-formylbenzoic acid.^12^ In another study published in 2016, benzoxazepines were synthesized through cyclization of enaminone derivatives in the presence of Cs_2_CO_3_.^13^ In the subsequent year, the synthesis involving tandem C–N coupling/C–H carbonylation transformations led to an alternative approach for obtaining 1,4-benzoxazepine derivatives.^14^ More recently, Zora and co-workers synthesized benzoxazepine derivatives using an InCl_3_-catalyzed, one-pot, two-component reaction.^15^

Apart from benzoxazepines, there’s a growing focus on exploring the biological activity associated with benzimidazoles.^16^ The benzimidazole structure is essentially a fused ring system consisting of a five-membered, cyclic, and aromatic imidazole ring containing two nitrogen atoms joined with a benzene ring. Given the extensive application of benzimidazole-containing systems as chemotherapeutic agents for treating diverse disorders, coupled with their notable safety, bioavailability, and stability, it is unsurprising that they hold significance as target molecules in medicinal chemistry.^17^ Examples of benzimidazole-containing systems include their potent activity against antihepatitis B virus (HBV),^18^ their antibacterial efficacy against *Salmonella typhimurium*,^19^ and their utilization as antimalarial agents,^20^ among various others. The benzimidazole component carries considerable significance in drug synthesis, driving the quest for an optimal strategy to access novel benzimidazole variants featuring diverse substituents.^21^ The traditional approach to synthesizing benzimidazoles primarily involves catalyst-free,^22,23^ as well as metal-^24,25^ and acid-catalyzed^26^ condensation reactions between 1,2-diaminobenzenes and aldehydes. Besides aldehydes, 1,2-diaminobenzenes are also known to react with primary alcohols,^27^ primary amines,^28^ β-keto esters,^29^ carboxylic acids to form benzimidazole derivatives.^30^ As illustrated by compounds **I** and **IV** in Figure 1, the fusion of benzoxazepine and benzimidazole units leads to the formation of benzimidazole-fused benzoxazepines, which exhibit significant bioactive properties.^5,8^

**Scheme 1:**
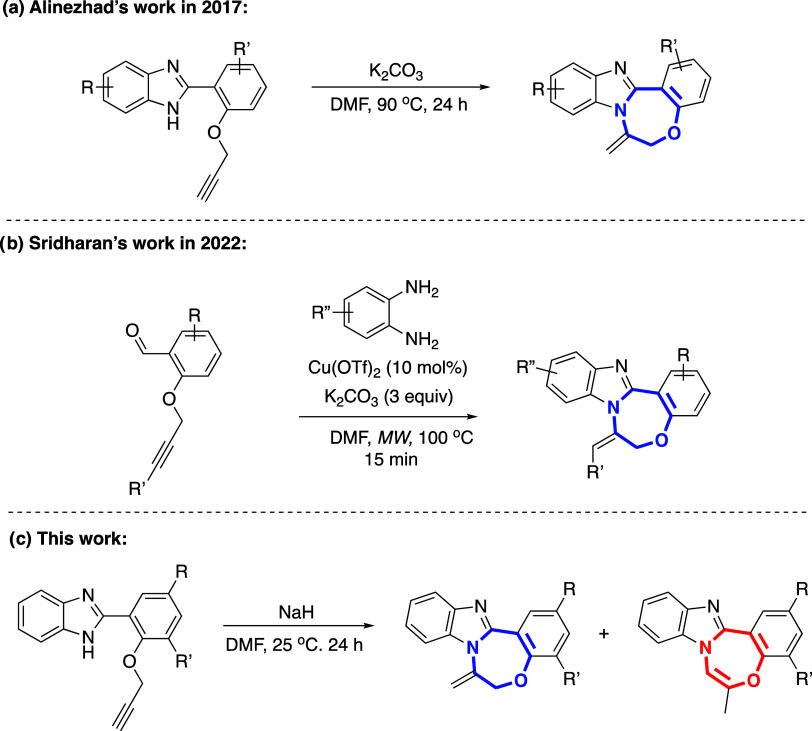
Comparison of Recent Approaches to Benzoxazepine-Imidazole Derivatives: Highlighting New Aspects of This Study in Contrast to the Existing Literature

In a recent study on the synthesis of benzoxazepine-imidazole derivatives, Alinezhad and co-workers utilized a transition metal-free approach involving base-mediated regioselective intramolecular 7-*exo-dig* hydroamination at 90 °C (Scheme 1).^31^

However, their research yielded only the *exo* product, as the *endo* product could not be obtained due to the utilization of K_2_CO_3_ as a base, which was unable to facilitate the 1,3-H shift process required to obtain the *endo*-configured product. In another study conducted in 2022, Rajput et al. developed the microwave-assisted copper(II)-catalyzed synthesis of benzimidazo-oxazepine via base-mediated 7-*exo-dig* cyclization.^32^ Herein, with the utilization of NaH as a base, we have observed the anticipated 7-*exo-dig* products as well as the occurrence of intermolecular propargyl transfer followed by intramolecular 7-*exo-dig* cascade reactions under ambient conditions, which have not been documented before. Additionally, as the temperature increased, it was noted that the *exo* products obtained in the basic medium underwent smooth conversion to *endo* products through a base-catalyzed 1,3-H shift.

## Results and Discussion

### Synthesis and Characterizations

**Scheme 2:**
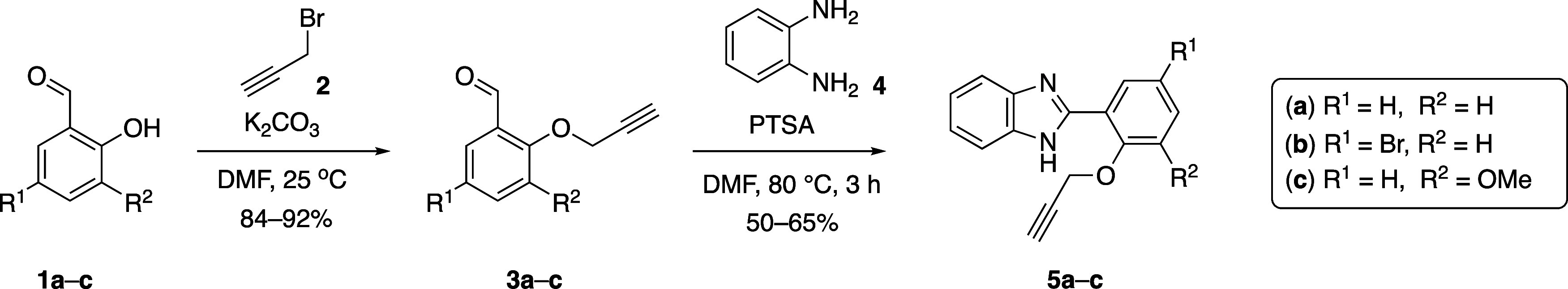
Synthetic Route for the Preparation of Substrates **5a**–**c**

In the first part of this work, we synthesized benzimidazole scaffolds **5a**–**c** intended for use in NaH-mediated cyclization reactions. The synthesis of **3a**–**c** started with the *O*-propargylation of salicylaldehyde derivatives **1a**–**c** using propargyl bromide (**2**) and K_2_CO_3_, following established literature procedures (Scheme 2).^33^ With **3a**–**c** in hand, substrates **5a**–**c** bearing terminal alkyne groups were obtained in 50–65% yields through condensation reactions with 1,2-diaminobenzene (**4**) in *N*,*N*-dimethylformamide (DMF) in the presence of PTSA.

**Scheme 3:**
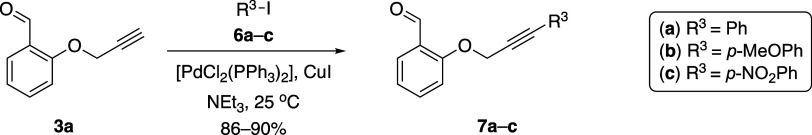
Sonogashira Cross-Coupling Reactions of Compound **3a** and Aryl Iodides **6a**–**c**

To examine variations in the behavior of substituted and terminal alkynes in base-mediated cyclization reactions, we performed Sonogashira cross-coupling reactions^34^ on **3a** to access disubstituted alkynes (Scheme 3). To systematically monitor the impact of electron density on the alkynes in base-mediated cyclization reactions, aryl iodides **6a**–**c** featuring electron-donating (−OMe), electron-withdrawing (−NO_2_), and neutral (−H) substituents were used in cross-coupling reactions and the desired compounds **7a**–**c** were obtained in yields ranging from 86 to 90%.

**Scheme 4:**
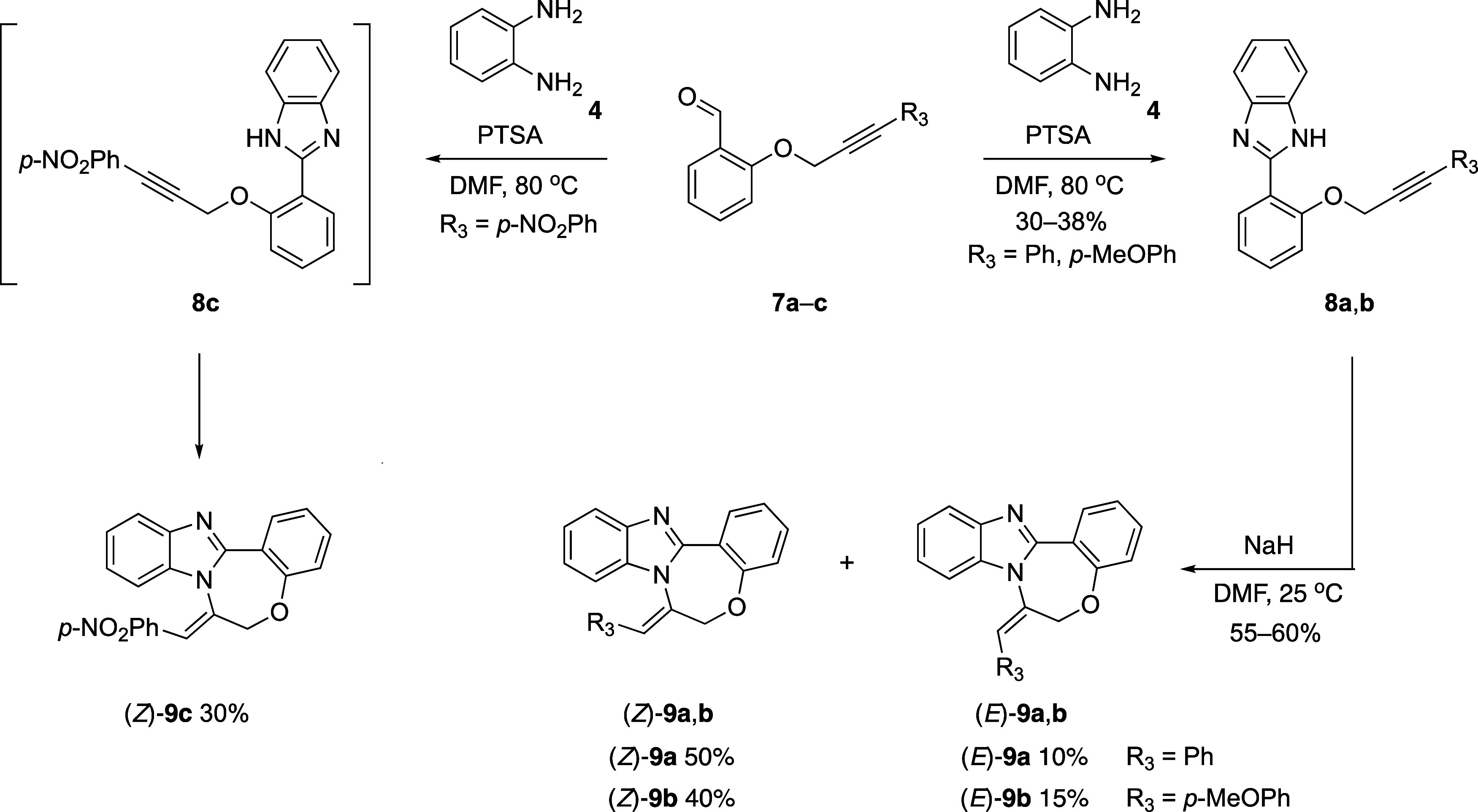
Condensation Reactions between *o*-Phenylenediamine (**4**) and Salicylaldehydes **7a**–**c**

In line with the synthesis of **5a**–**c** outlined in Scheme 2, key substrates **8a**–**c** incorporating benzimidazole units were attempted to be synthesized by reacting salicylaldehyde derivatives **7a**–**c** with disubstituted alkyne groups in the presence of 1,2-diaminobenzene (**4**) (Scheme 4). When *R*^3^ was Ph or *p*-MeOPh, the desired compounds **8a** and **8b** were smoothly synthesized in yields of 30 and 38%, respectively. However, when *R*^3^ = *p*-NO_2_Ph, the reaction did not cease after the formation of imidazole **8c** but proceeded further to yield the cyclized product (*Z*)-**9c**. When imidazole-substituted substrates **8a** and **8b**, in which spontaneous cyclization did not occur in the preceding step, were treated with NaH in DMF at room temperature, the desired benzoxazepine derivatives were obtained in both *E* and *Z* isomeric forms, with the *Z* isomer being the predominant product (*E*/*Z* = 10:50 for **9a** and *E*/*Z* = 15:40 for **9b**). To account for this difference, the energies of *E*/*Z* isomers were compared by using density functional theory (DFT) calculations. A preference of 1.57 kcal/mol for (*Z*)-**9a** and 3.04 kcal/mol for (*Z*)-**9b** was observed over their respective (*E*) isomers in both instances (see Figure S37 in the SI).

**Scheme 5:**
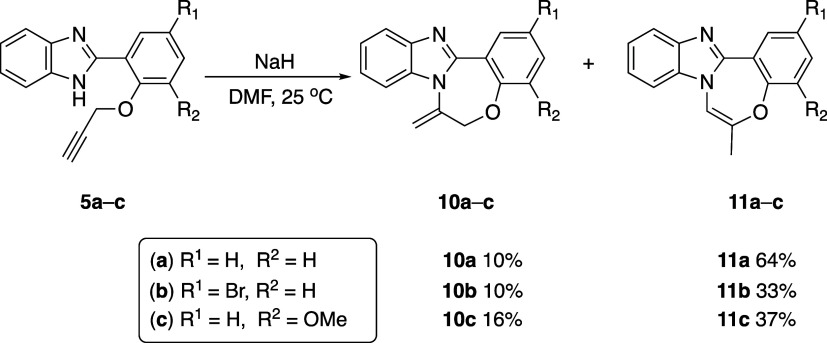
Base-Mediated Cyclization Reactions of Substrates **5a**–**c**

Following successful cyclization attempts with substrates bearing disubstituted alkynes **8a**–**c**, our focus turned to 7-*exo-dig* cyclization reactions involving substrates containing terminal alkynes **5a**–**c** (Scheme 5).

**Scheme 6:**
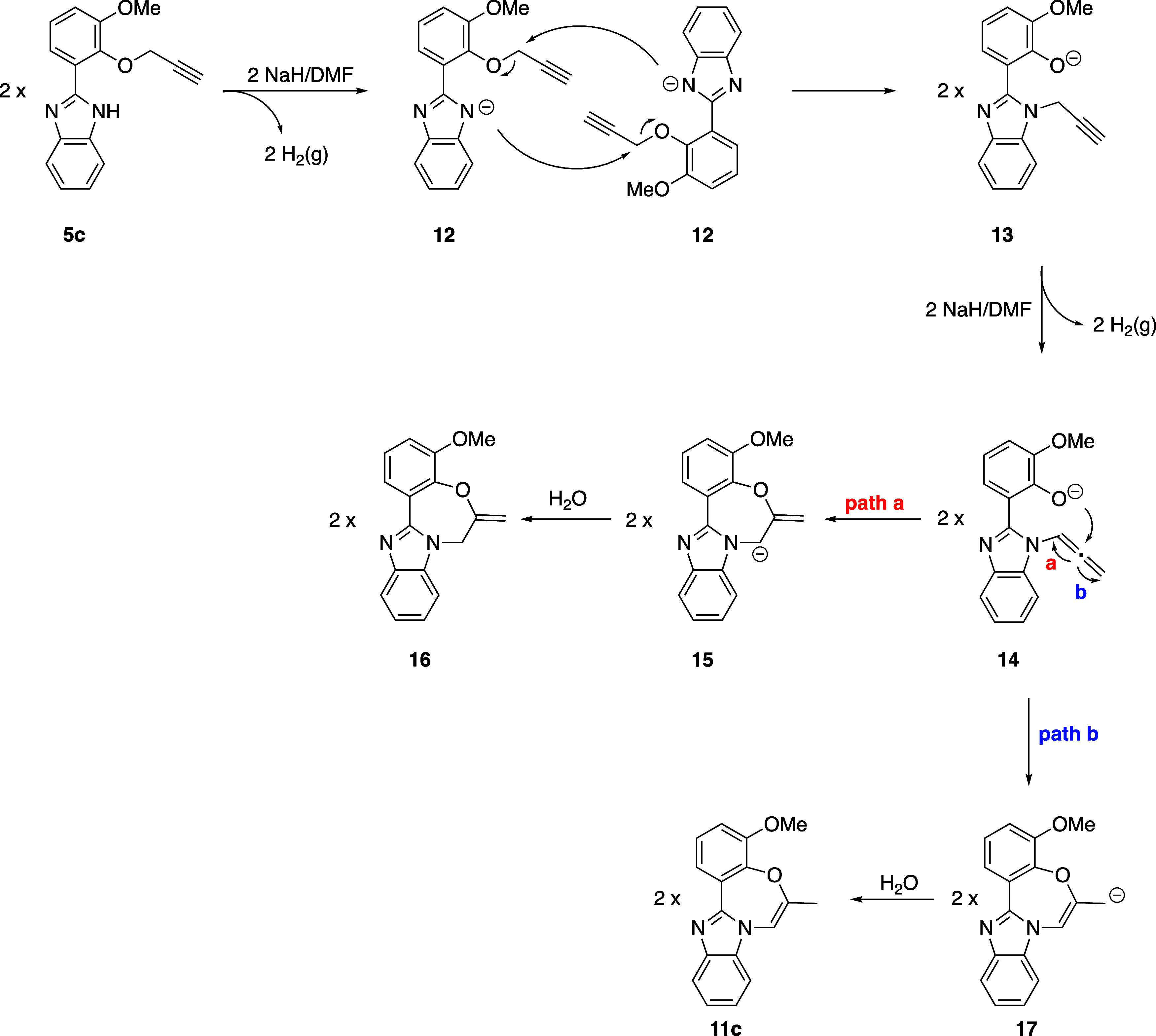
Proposed Mechanism for the Formation of **11c**

In our optimized conditions, we aimed to determine whether we could obtain the endocyclic product, which has not been reported to occur in the existing literature,^30^ alongside the exocyclic product. Treatment of terminal alkyne-containing substrates **5a**–**c** with NaH in DMF resulted in the formation of two different cyclization products. Consistent with the literature, the initial group of products **10a**–**c** were identified as the exocyclic products, resulting from a 7-*exo-dig* cyclizations, with yields ranging from 10 to 16%. Unexpectedly, the second group of products **11a**–**c** did not correspond to the anticipated endocyclic products; rather, we observed a molecular rearrangement. Although it was initially thought that **11a**–**c** could be formed by intramolecular propargyl transfer followed by 7-*exo-dig* cyclization and 1,3-H shift, the 6-*endotet* ring closure reaction required for propargyl transfer is disfavored by Baldwin’s rules. Hence, we presume that the propargyl transfer reaction occurs as an intermolecular process involving two separate substrates (Scheme 6). The proposed mechanism for the formation of the representative compound **11c** is depicted in Scheme 6.

The removal of an acidic NH proton results in the formation of anion **12**, which then undergoes an intermolecular nucleophilic substitution reaction by attacking the methylene group. This process facilitates the transfer of the propargyl group from the oxygen atom to the nitrogen atom, yielding compound **13**. Subsequently, the propargyl group is converted to an allene group^35^ and then the phenoxide ion attacks the central carbon of the allene, resulting in the formation of compounds **15** and **17**. In the last step, **11c** and **16** can be formed because of an aqueous workup process. To our knowledge, the propargyl transformation reported here is unprecedented in the existing literature.

**Figure 2:**
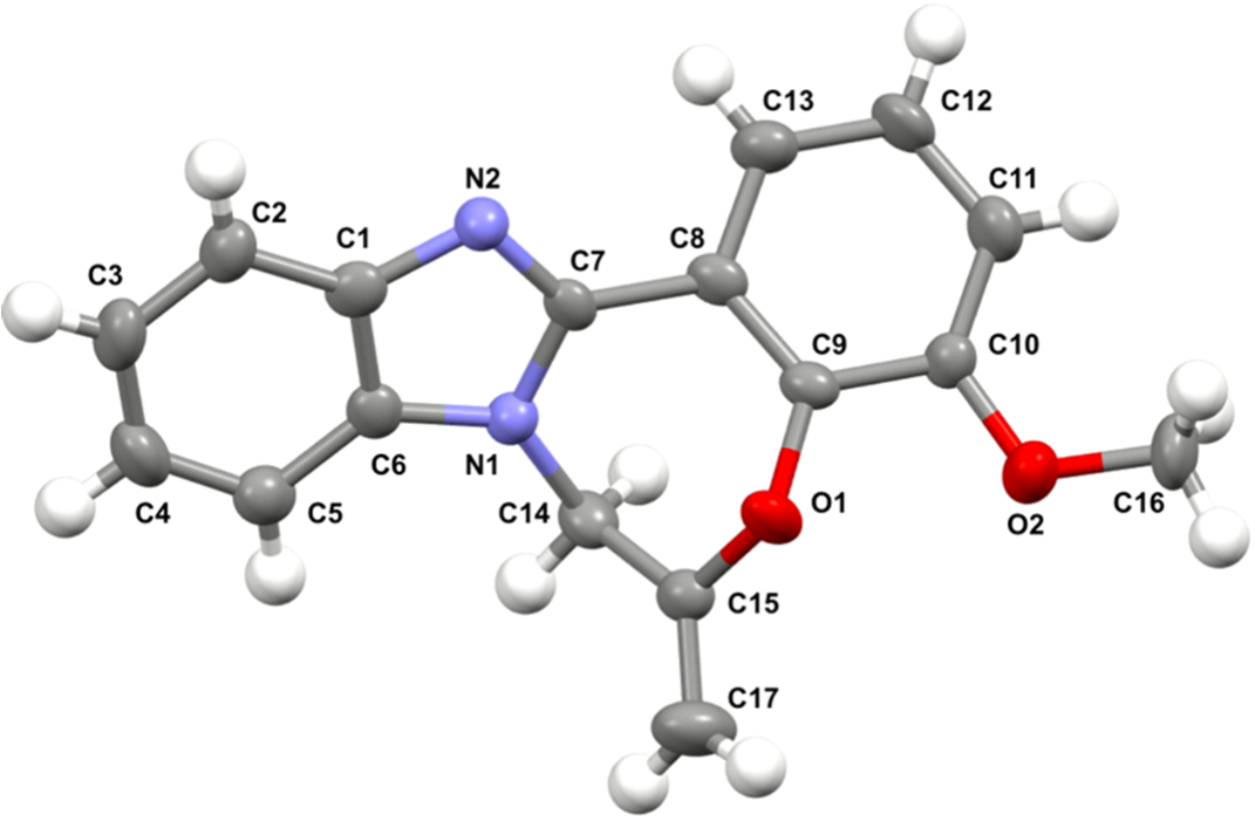
ORTEP representation of **16** with vibrational ellipsoids shown at the 50% probability level, with arbitrary numbering.

In the cyclization reactions described in Scheme 5, which were conducted over a 24-h period, no exocyclic product **16** was detected in the medium following propargyl transfer. However, with the shortening of the reaction time, product **16** was observed via ^1^H nuclear magnetic resonance (NMR) spectroscopy. Fortunately, it was possible to isolate and crystallize it using a slow vapor deposition technique (chloroform/hexane 1:1) from the crude mixture, and its structure was confirmed through X-ray analysis (Figure 2).

**Scheme 7:**
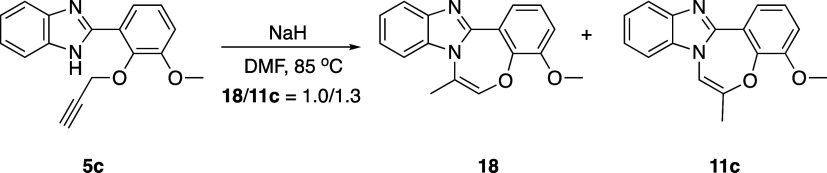
Base-Mediated Cyclization Reactions of Substrate **5c** at 85 °C

Upon careful analysis of the reaction depicted in Scheme 5, conducted at room temperature, it is interesting that one of the products exhibits an *exo* configuration, while the other displays an *endo* configuration. The reaction was repeated at 85 °C, considering that the activation energy barrier necessary for the conversion of *exo*-configured products **10a**–**c** to *endo*-configured products might not be achievable at room temperature (Scheme 7). As expected, only the formation of two distinct *endo*-configured isomers (**18**/**11c** = 1.0/1.3, based on ^1^H NMR) was observed. Unfortunately, despite all our efforts, such as column chromatography (CC) on silica gel or neutral/basic aluminum oxide and crystallizations, attempts to separate endocyclic products were unsuccessful (see Figure S27 in the Supporting Information).

**Scheme 8:**
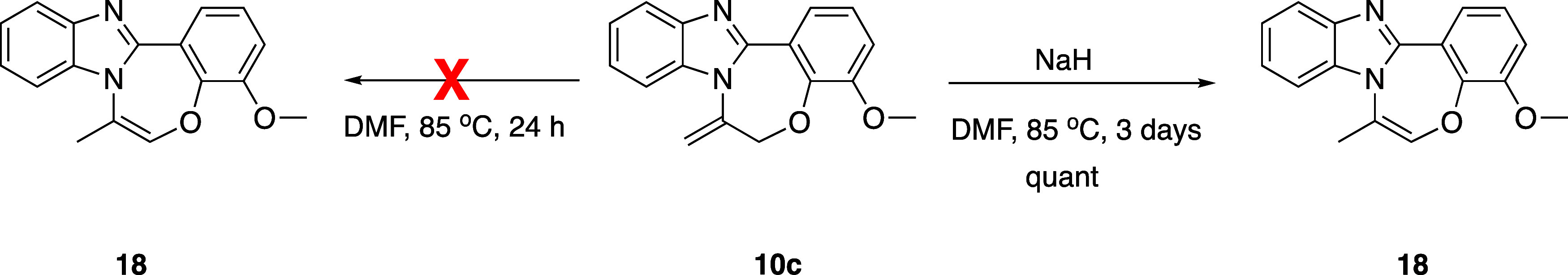
Base-Mediated Cyclization Reactions of Substrate **5c** at 85 °C

To ensure conversion between *endo* and *exo* products, pure *exo* product **10c** was kept in DMF at 85 °C for 24 h without the presence of a base, and no conversion was observed. Conducting the same experiment in the presence of NaH revealed that the *exo* isomer underwent complete conversion to the *endo* isomer within 3 days (Scheme 8).

**Figure 3:**
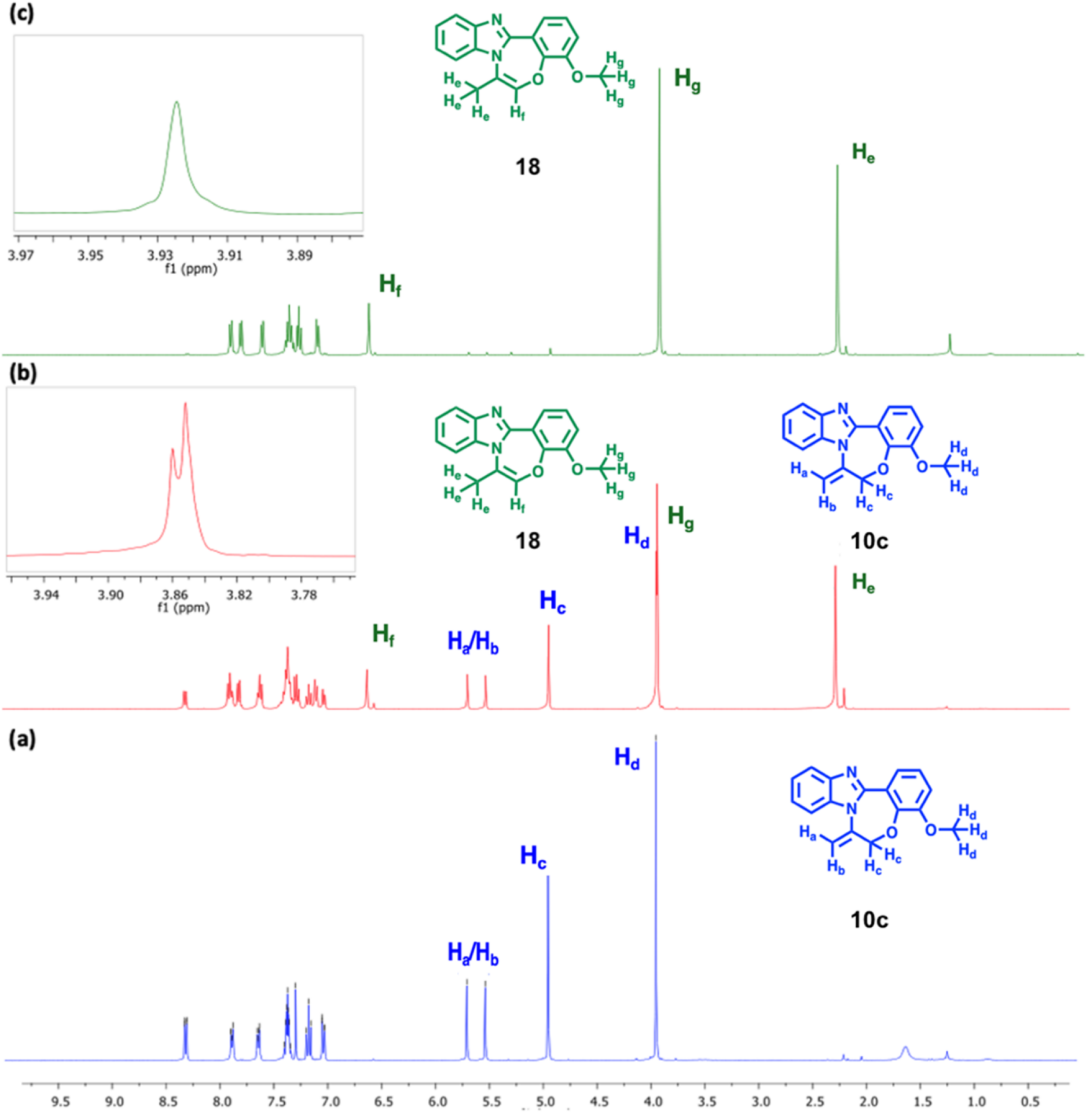
^1^H NMR (400 MHz) monitoring of the transformation process from **10c** to **18** (a) 0 h, (b) after 20 h, and (c) after 72 h.

Figure 3 illustrates the ^1^H NMR analysis depicting the transformation process from **10c** to **18**. Partial conversion to **18** was observed approximately 24 h after heating of pure **10c** in the presence of NaH in DMF (in an oil bath) (Figure 3b), while complete conversion is achieved approximately 3 days later (Figure 3c). This conversion can be easily followed by following the methoxy signals between about 3.80 and 3.90 parts per million (ppm).

### Computational Studies

The formation of cyclization products **10a** and **11a** were computationally investigated using the Gaussian 09 program package.^36^ Geometry optimizations and frequency calculations of reactants, products, transition states (TSs), and intermediates were fully optimized with M06–2X hybrid functional by using the 6–31+G(d,p) basis set in gas phase unless otherwise stated.^37,38^ To confirm that each transition state connects the corresponding reactant and the product, the Intrinsic Reaction Coordinate (IRC) path was followed.^39,40^ Single Point Energy (SPE) calculations were carried out with the Polarizable Continuum Model (PCM)^41^ at the M06–2*X*/6–311++G(d,p) level in *N*,*N*-dimethylformamide (DMF) as a solvent, since this solvent was used in the experimental studies. Gibbs free energy corrections were calculated for the gas or solvent phase stationary points and were added to single point PCM energies to compute Gibbs free energy values in the solution phase. The optimized geometry structures are illustrated by *Chemcraft.*(42)

### Mechanism for the Formation of 7-*exo-dig* Product **10a**

**Scheme 9:**
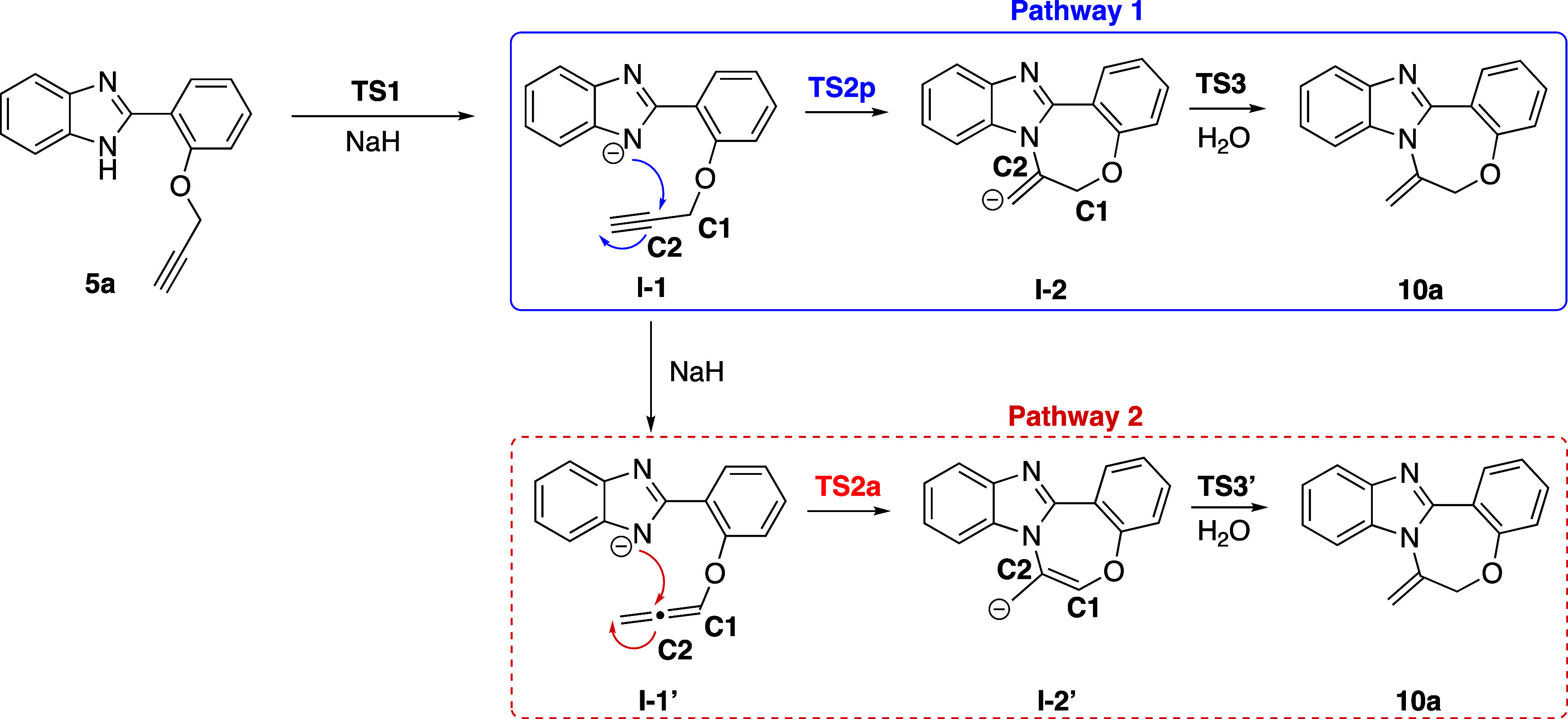
Two Potential Pathways (Blue and Red) for the Formation of Product **10a**

We began our DFT calculations to explore potential mechanisms for the formation of product **10a**, building on the research conducted by Mahdavi et al.^43^ This study thoroughly examined the 6-*exo-dig* cyclizations of substrates containing both indole and propargylamine units in the presence of KO^*t*^Bu in DMF. In this study, Mahdavi et al. suggested two distinct mechanisms: one involving the deprotonation of the indole NH group and the other the formation of an allenyl anion as the initial step. Their findings indicated that the first mechanism aligns more closely with the DFT calculations. Considering the structural similarities between the substrates in this study and those in the previously mentioned study by Mahdavi et al.,^43^ along with the fact that 7-*exo-dig* cyclizations, like 6-*exo-dig* ones, are allowed under Baldwin’s rules, two analogous pathways for the conversion of substrate **5a** to **10a** in the current study were considered (Scheme 9).

**Figure 4:**
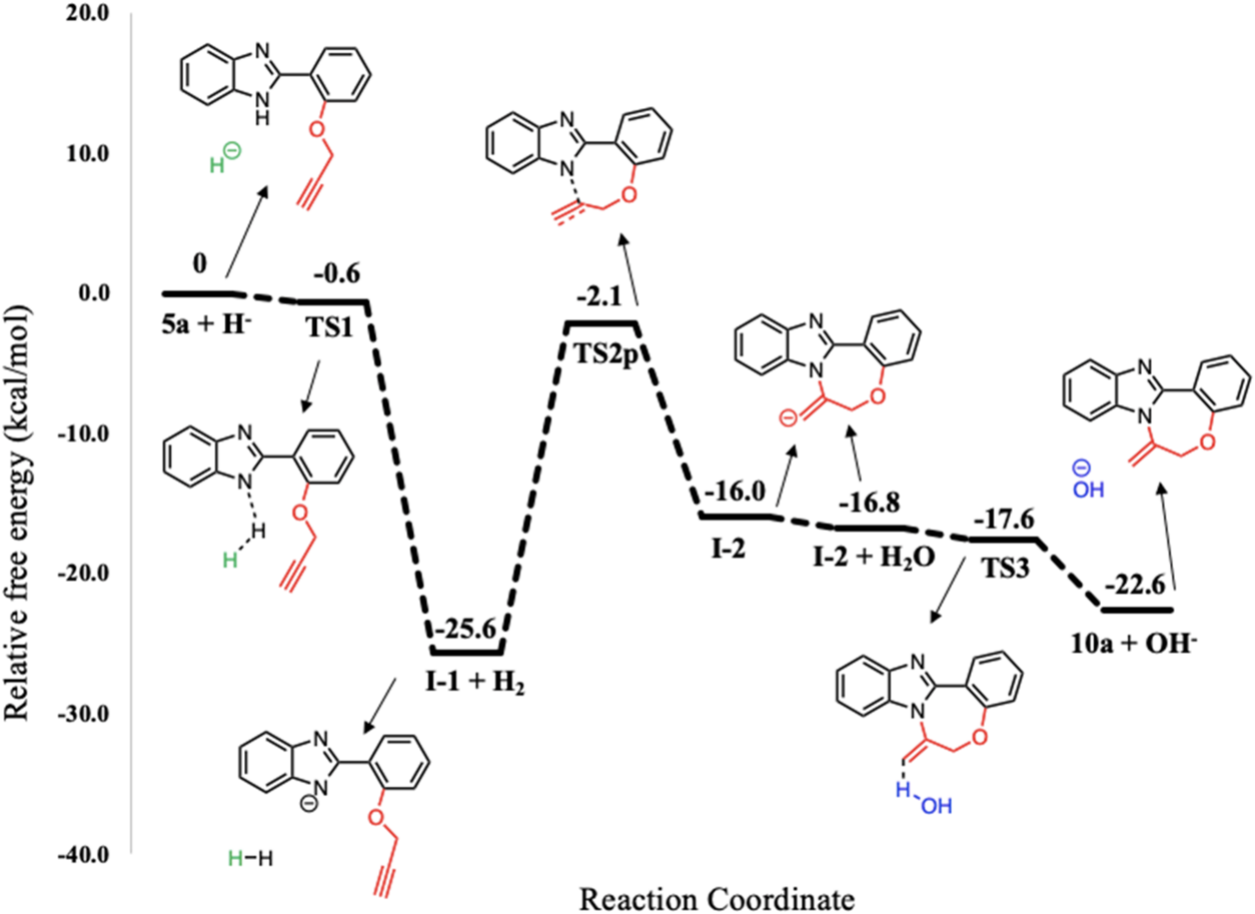
Potential energy profile related to the formation of 7-*exo-dig* product **10a** (Pathway 1) at the PCM/M06–2*X*/6–311++G(d,p)//M06–2*X*/6–31+G(d,p) level in DMF. The energies for **5a + H**^**–**^, **TS1**, and **I-1 + H**_**2**_ were initially optimized at the M06–2*X*/6–31+G(d,p) level in DMF and then SPE calculations were performed at the PCM/M06–2*X*/6–311++G(d,p) level in DMF.

The first step, which involves the formation of **I-1** through the abstraction of the N–H proton from the benzimidazole ring, is common for both suggested Pathways 1 and 2. This step follows a barrierless pathway, exhibiting a very low transition state energy that is very close to the corresponding reactant **5a** (Figure 4).^44^

**Figure 5:**
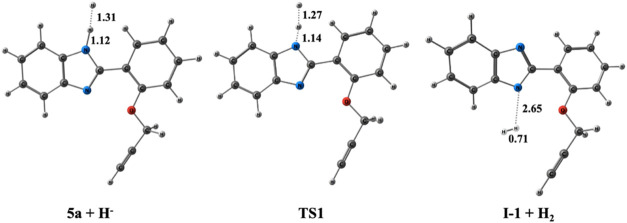
Optimized geometries for the stationary points for the formation of **I-1**. The energies were initially optimized at the M06–2*X*/6–31+G(d,p) level in DMF and then SPE calculations were performed at the PCM/M06–2*X*/6–311++G(d,p) level in DMF. Distances are given in Å.

The optimized geometries and bond lengths of each stationary point are depicted in Figure 5.

**Figure 6:**
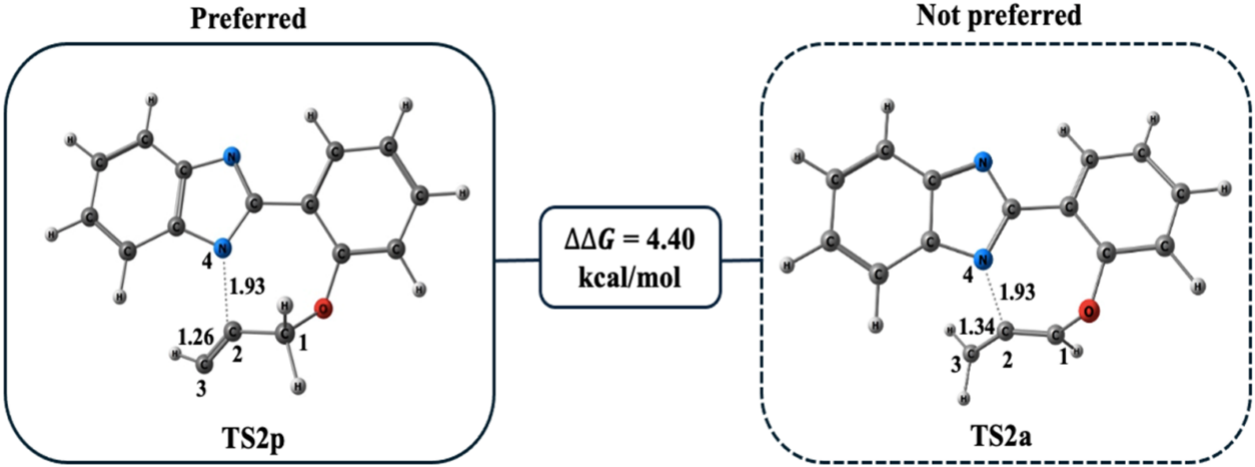
Optimized geometries for **TS 2p** and **TS2a** at the PCM/M06–2*X*/6–311++G(d,p)//M06–2*X*/6–31+G(d,p) level in DMF. Distances are given in Å.

There are two possible routes leading to cyclization: Pathway 1 and 2 (Scheme 9). In Pathway 1, cyclization proceeds through **TS 2p** (**p**:propargyl) starting from propargyl intermediate **I-1**. In Pathway 2, cyclization is initiated by the allene isomer **I-1′**, following **TS2a** (**a**:allene). Modeling of both transition states revealed a 4.40 kcal/mol preference for **TS 2p**, indicating the reaction follows Pathway 1 (Figure 6).

**Figure 7:**
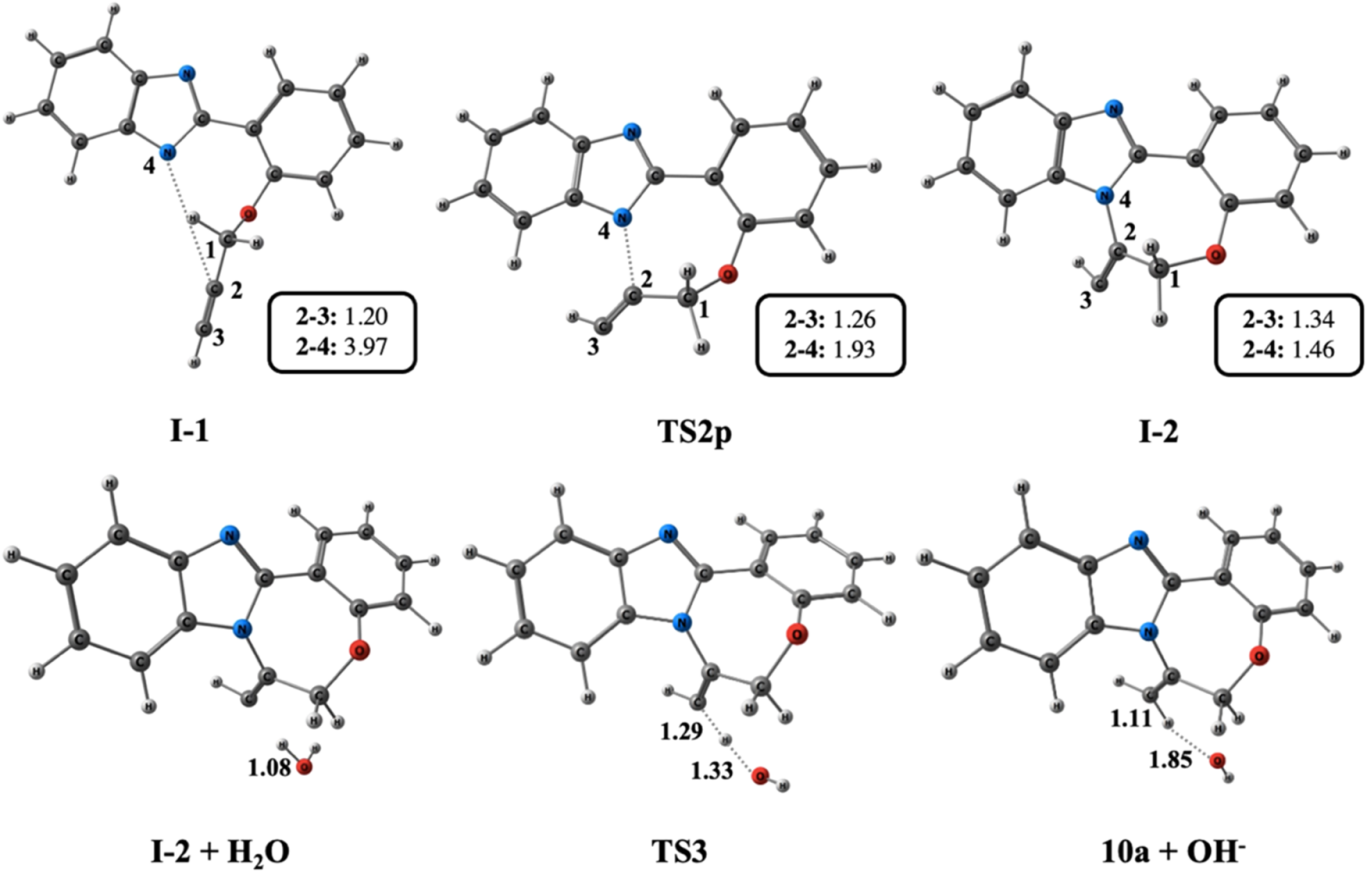
Optimized geometries for the stationary points of the formation of **10a** at the PCM/M06–2*X*/6–311++G(d,p)//M06–2*X*/6–31+G(d,p) level in DMF. Distances are given in Å.

Cyclization emerges from **TS 2p**, where the nitrogen anion performs a nucleophilic attack on propargyl **C2**. During the formation of benzimidazole nitrogen **N4** and propargyl **C2** bond, the bond distance decreases from 1.93 Å in **TS 2p** to 1.46 Å in **I-2**. Meanwhile, the triple **C2** and **C1** bond length increases from 1.26 Å in **TS 2p** to 1.34 Å in **I-2** (Figure 7). The final step occurs through a barrierless pathway, similar to the initial step, where the negative charge on **I-2** is neutralized by water (introduced during the aqueous workup process), resulting in the formation of the cyclization product **10a**. The potential energy profile for Pathway 1 is depicted in Figure 4, where the formation of **10a** is exergonic by 22.6 kcal/mol relative to **5a + H**^**–**^. The optimized geometries and bond lengths of each stationary point are illustrated in Figure 7.

Considering the activation barriers of Pathways 1 and 2, Pathway 1, which involves cyclization via the propargyl intermediate, was found to be the energetically favorable route.

### Mechanism for the Formation of Product **11a**

**Scheme 10:**
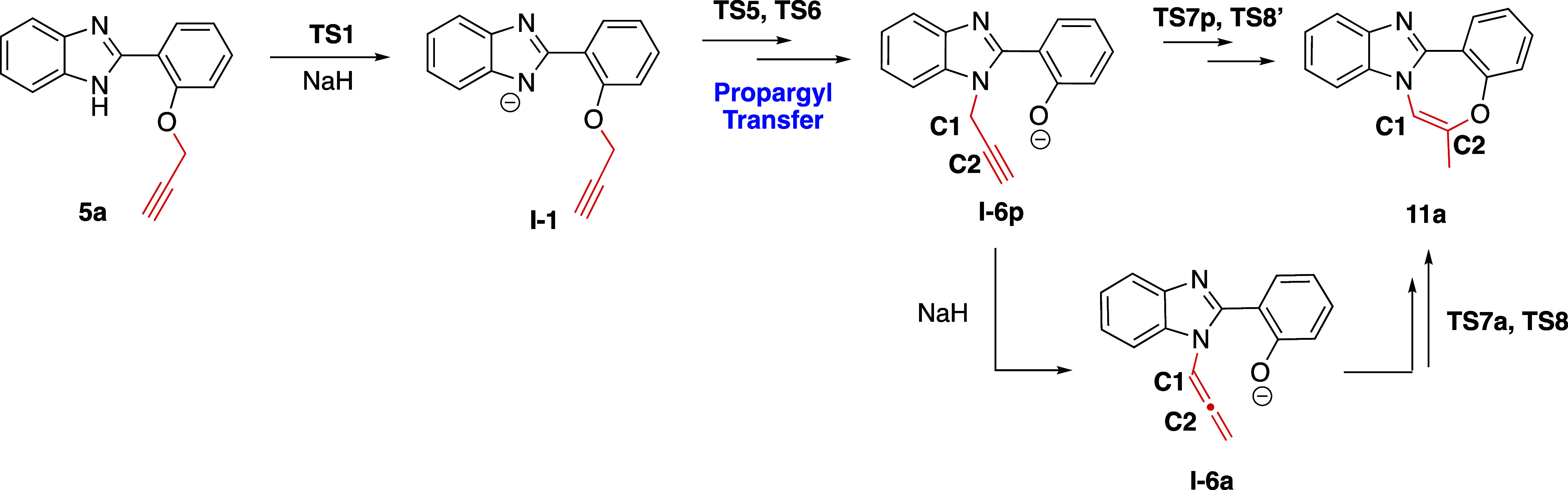
General Scheme for the Formation of **11a**

Upon the cyclization of **5a**, another cyclization product (**11a**) was isolated as well. In order to explain the cyclization mechanism, the formation of **11a** was also computationally investigated (Scheme 10).

**Scheme 11:**
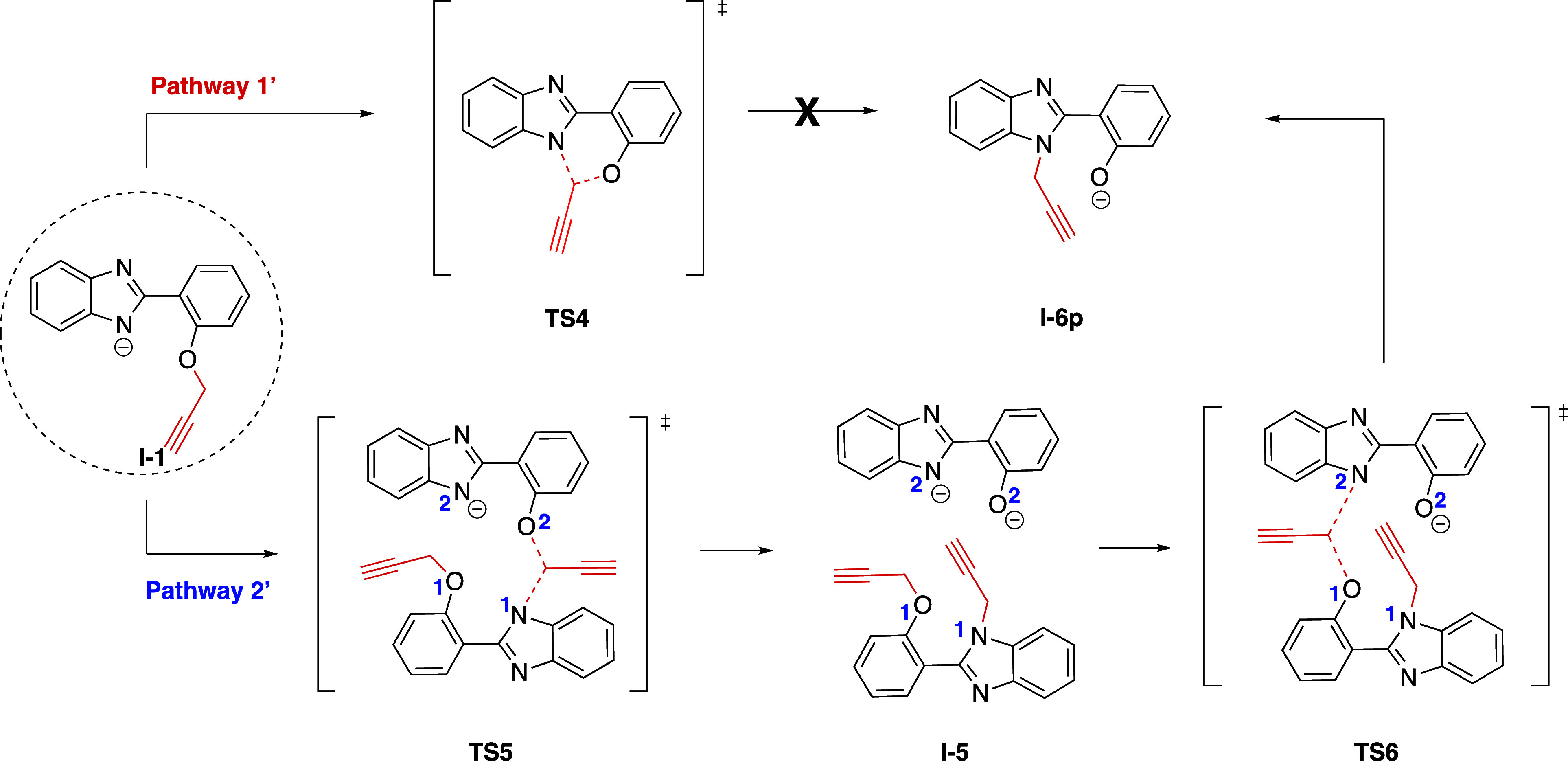
Intramolecular (Pathway 1′) and Intermolecular (Pathway 2′) Propargyl Transfers Leading to **I-6p**

The initial step for the formation of **I-1** is common to both proposed pathways, Pathway 1′ and Pathway 2′ (Scheme 11). Pathway 1′ proceeds through **TS4**, involving an intramolecular propargyl transfer. This transfer follows a 6-*endo-trig*-like transition state, which is not permitted according to Baldwin’s rules. Our computational results also indicated that reaching **TS4** requires 68.4 kcal/mol of energy in solvent (DMF), suggesting that this route is not plausible.

**Figure 8:**
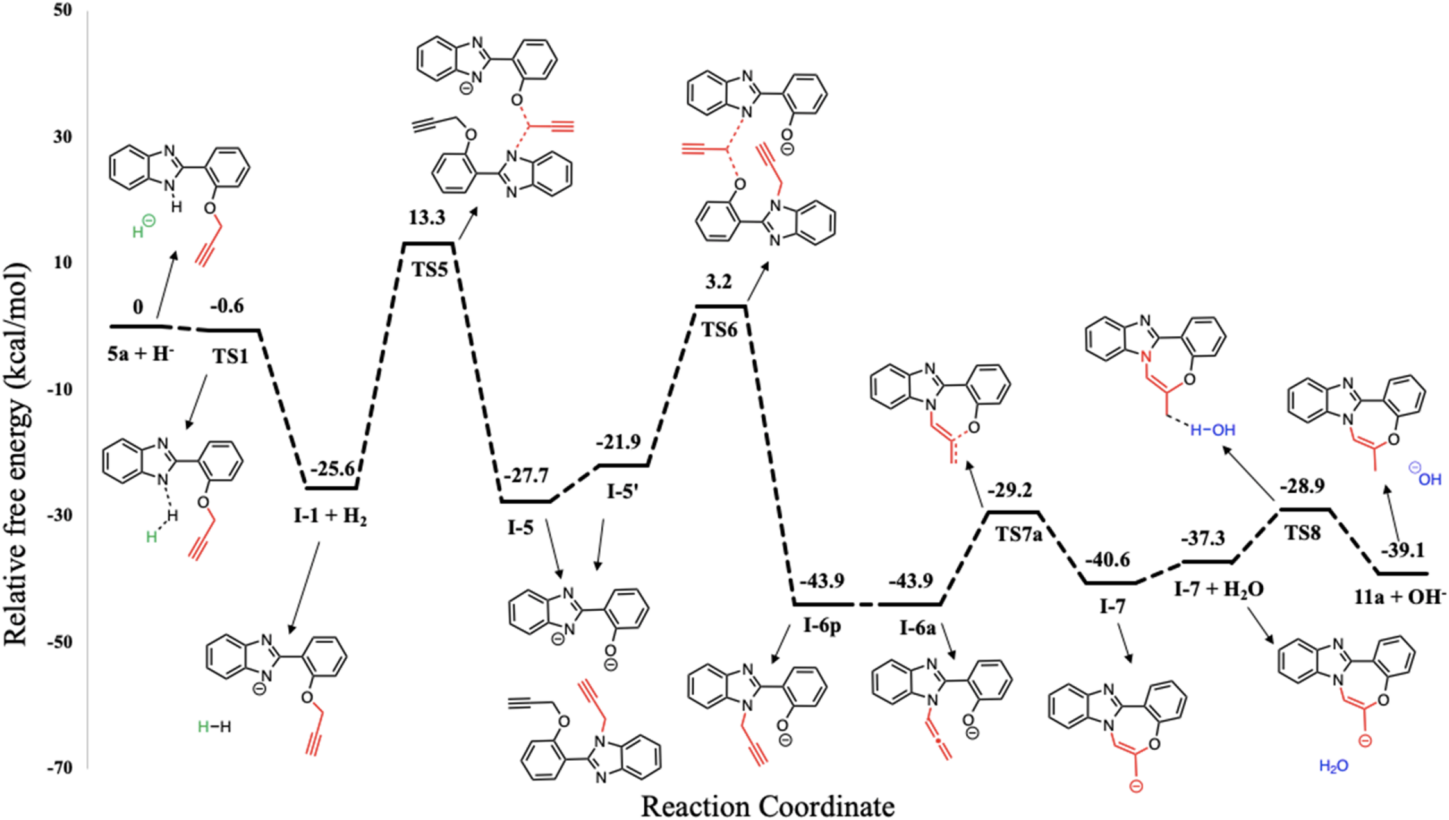
Potential energy profile related to the formation of propargyl transfer product **11a** at the PCM/M06–2*X*/6–311++G(d,p)//M06–2*X*/6–31+G(d,p) level in DMF. The energies for **5a + H**^**–**^, **TS1**, and **I-1 + H**_**2**_ were initially optimized at the M06–2*X*/6–31+G(d,p) level in DMF and then SPE calculations were performed at the PCM/M06–2*X*/6–311++G(d,p) level in DMF.

Alternatively, the mechanism might proceed through Pathway 2′, where intermolecular propargyl transfer occurs between two **I-1** intermediates. Following the abstraction of a proton from the N–H group on the benzimidazole ring, the subsequent step involves propargyl transfer from O2 to N1, with a Gibbs free energy barrier of 13.3 kcal/mol relative to **5a + H**^**–**^. The following step of Pathway 2′ involves the transfer of another propargyl from O1 to N2, passing through **TS6** and forming **I-6p**. This step requires an activation free energy of 3.2 kcal/mol relative to the initial reactant complex **5a + H**^**–**^ (Figure 8). Clearly, Pathway 2′ is energetically more favorable than Pathway 1′.

**Figure 9:**
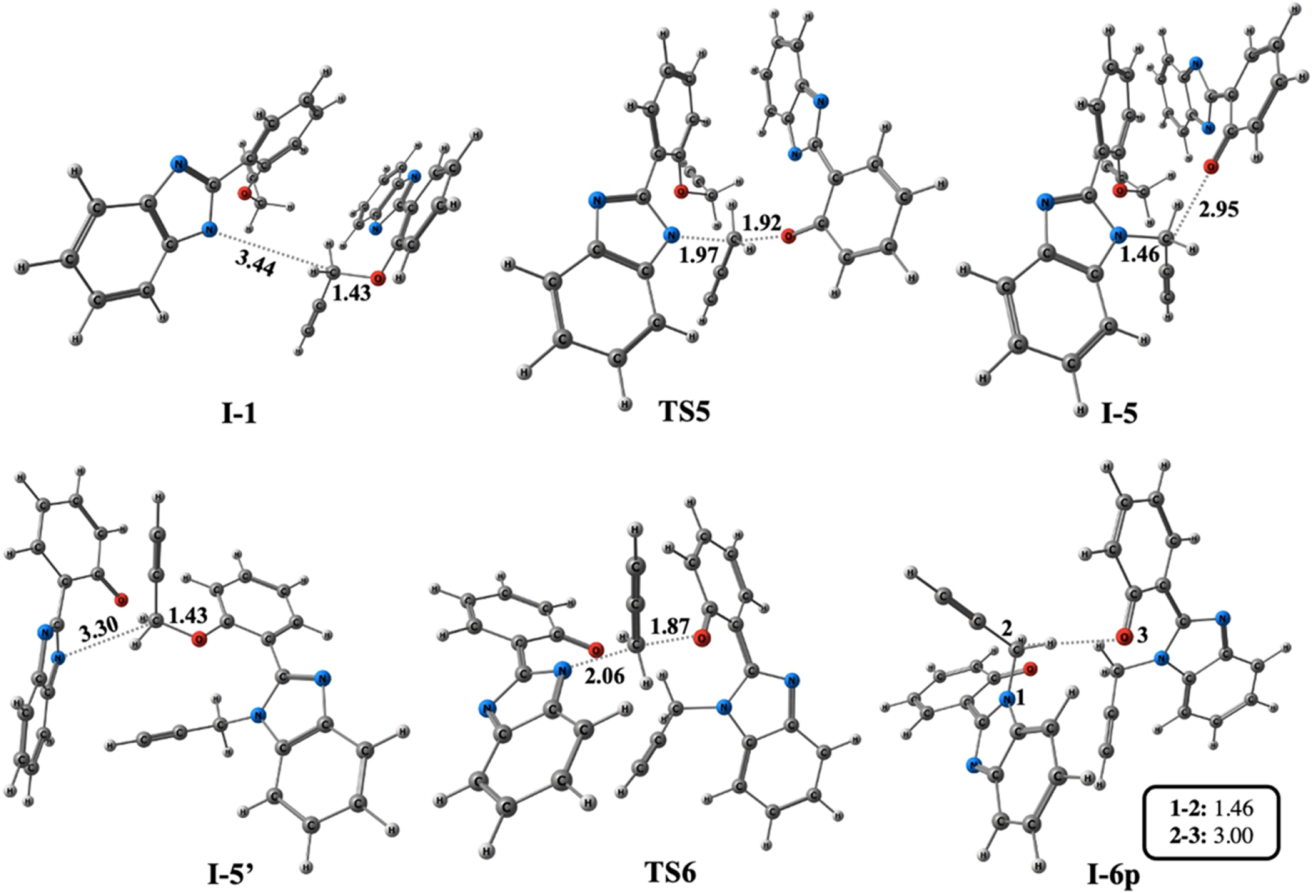
Optimized geometries for the stationary points of the formation of **I-6p** at the PCM/M06–2*X*/6–311++G(d,p)//M06–2*X*/6–31+G(d,p) level in DMF. Distances are given in Å.

The optimized geometries of **5a + H**^**–**^, **TS1**, and **I-1** were previously presented in Figure 5. The optimized geometries and bond lengths corresponding to propargyl transfer are illustrated in Figure 9.

**Figure 10:**
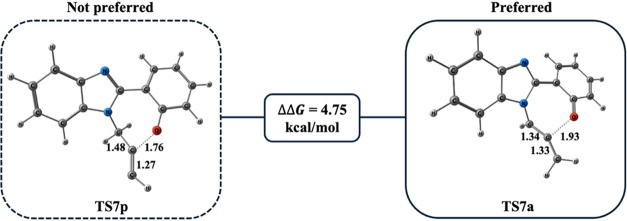
Optimized geometries for **TS7p** and **TS7a** at the PCM/M06–2*X*/6–311++G(d,p)//M06–2*X*/6–31+G(d,p) level in DMF. Distances are given in Å.

Following the propargyl transfer, intramolecular cyclization may proceed through either the propargyl intermediate **I-6p** (**p**:propargyl) or its allene isomer **I-6a** (**a**:allene) (Scheme 10). Both transition states (**TS7a** and **TS7p**) were modeled, and our calculations showed a 4.75 kcal/mol preference for **TS7a** over **TS7p** (Figure 10).

**Scheme 12:**
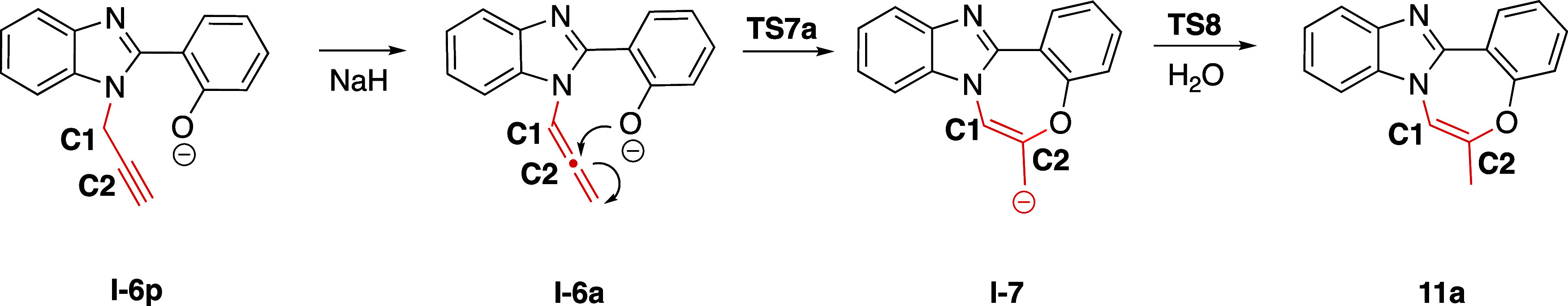
Proposed Pathway for the Formation of **11a**

Consequently, we inferred that this reaction proceeds through the allene intermediate (Scheme 12). The subsequent step involves forming the cyclization intermediate **I-7** which occurs via the nucleophilic attack of the oxygen anion on the central carbon atom of the allene moiety since it is more electropositive, as demonstrated in our previous study.^35^

**Figure 11:**
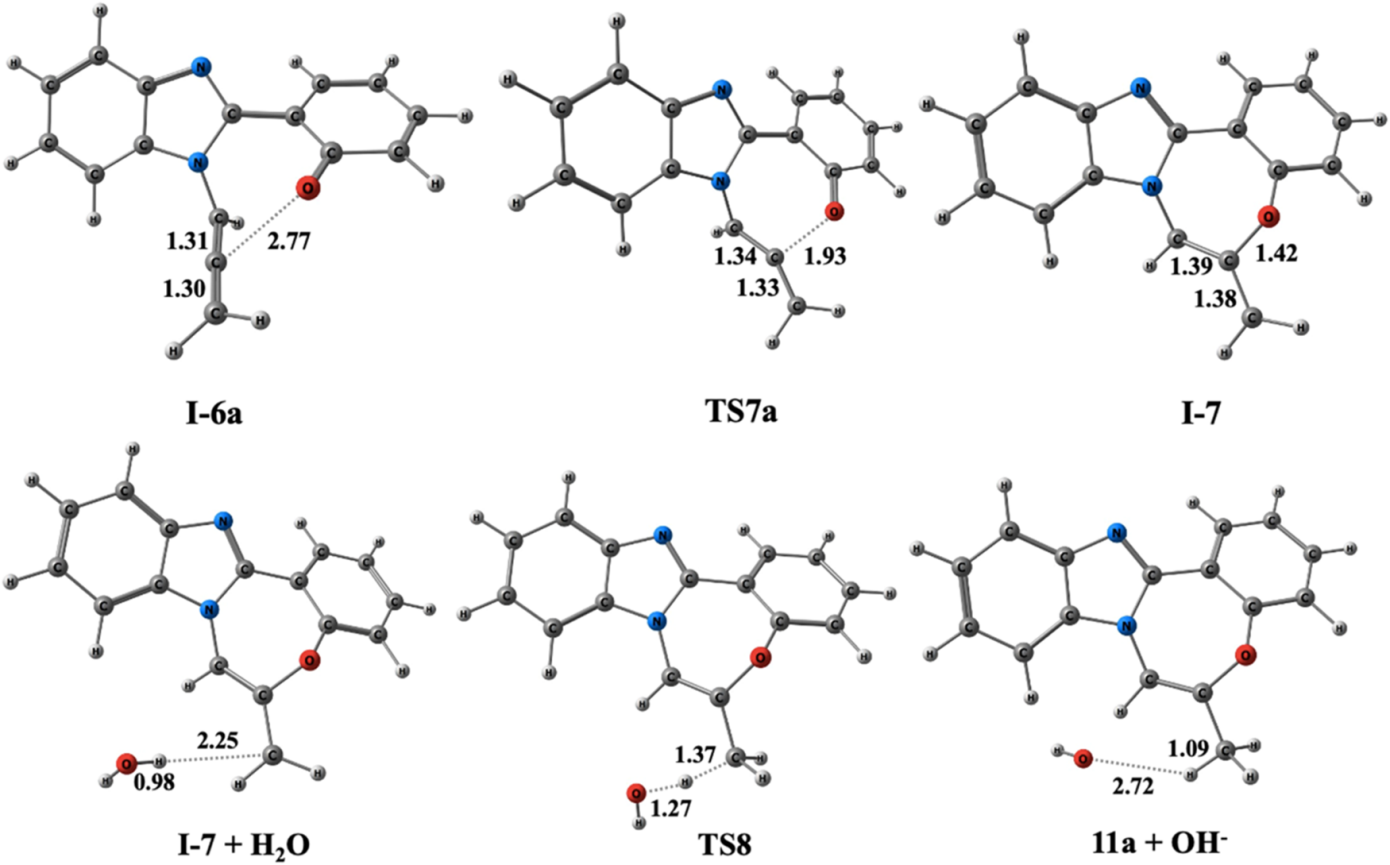
Optimized geometries for the stationary points of the formation of **11a** at the PCM/M06–2*X*/6–311++G(d,p)//M06–2*X*/6–31+G(d,p) level in DMF. Distances are given in Å.

The final step involves **TS8**, which includes proton abstraction from water to yield cyclization product **11a**. The potential energy profile for the formation of **11a** is depicted in Figure 8. The overall reaction is exergonic by 39.1 kcal/mol. Figure 11 illustrates the optimized geometries and bond lengths of each stationary point.

## Conclusions

In this work, a total of 12 new benzimidazole-fused benzoxazepines were successfully synthesized via base-mediated cyclization reactions. In the experiments with substrates containing disubstituted alkynes, two distinct cyclization pathways were observed, depending on the electronic properties of the side groups. In the substrate containing *p*-nitrophenyl group, cyclization was carried out under acidic conditions where the imidazole group was incorporated into the structure, whereas substrates containing *p*-methoxyphenyl and phenyl groups were converted into target products upon treatment with NaH in DMF. The outcomes observed in the cyclization experiments involving substrates containing terminal alkynes were rather surprising. Although our theoretical expectations pointed toward obtaining two different *exo*- and *endo*-configured products through 7-*exo-dig* cyclization reactions, along with a potential 1,3-H shift, only the *exo* product was found in minor amounts. Unexpectedly, the major product obtained exhibited an isomeric structure different from what was anticipated. This product is proposed to have been obtained via intermolecular propargyl transfer, followed by intramolecular 7-*exo-dig* cyclization/1,3-H shift cascade transformations, aligning with the Baldwin rules. To the best of our knowledge, this transformation appears to be unprecedented in the literature. Additional mechanistic studies revealed that only *endo* products were obtained when cyclization reactions were carried out at 85 °C instead of 25 °C. Furthermore, it was found that only applying heat is insufficient for the conversion of exocyclic products to endocyclic products; the presence of NaH in the medium is also essential for this transformation. In the computational part of the study, the mechanisms leading to cyclization products **10a** and **11a** were examined in detail by means of DFT calculations. In both pathways, the initial reaction step involves proton abstraction from the benzimidazole nitrogen. The 7-*exo-dig* cyclization proceeds via the propargyl intermediate, aligning with existing literature findings and Baldwin’s rules. For the formation of **11a**, the most favorable route involves propargyl-allene isomerization, following two propargyl transfers. The transition state energy of intramolecular propargyl transfer was determined to be significantly high. As a result, a cyclization mechanism starting at the allene carbon, which leads to final product **11a**, was identified as the most energetically favorable pathway. The potential energy profiles for the formation of **10a** and **11a** show a downward trend relative to the reactants, indicating that both proposed mechanisms are energetically favorable.

## Experimental Section

### General

Commercially available chemicals were used without subjecting them to additional purification steps. Compounds **3a**–**c**,^31^**5a**–**c**,^31^**7a**,^32^**7b**,^32^**7c**,^45^**8a**,^32^ (*E*)-**9a**,^31^ and **10a**–**c**^30^ were synthesized according to the existing literature. Solvents used for workup and purification steps were distilled prior to use. Sonogashira cross-coupling reactions were conducted under a nitrogen atmosphere using oven-dry glassware. Column chromatography (CC) using SiO_2_-60 mesh was used for the purifications of the target structures. Analytical thin-layer chromatography (TLC) was performed on aluminum sheets coated with 0.2 mm silica gel 60 F254, and visualization was achieved by using a UV lamp (254 or 366 nm). The solvents were evaporated under vacuum at temperatures ranging from 25 to 60 °C and pressures between 900 and 10 mbar. ^1^H and ^13^C{^1^H} nuclear magnetic resonance (NMR) spectra were acquired at frequencies of 400 MHz for ^1^H and 100 MHz for ^13^C{^1^H}, respectively. Chemical shifts (δ) are expressed in parts per million (ppm) relative to tetramethylsilane (TMS), utilizing the residual deuterated solvent signal as an internal reference (CDCl_3_: δ_H_ = 7.26 ppm, δ_C_ = 77.0 ppm). In ^1^H NMR spectroscopy, resonance multiplicity is denoted as s (singlet), d (doublet), t (triplet), q (quartet), quint (quintet), sext (sextet), sept (septet), m (multiplet), and br. (broad). Coupling constants (*J*) are provided in Hertz (Hz). Additionally, all of the spectra were acquired at room temperature. High-resolution mass spectrometry (HRMS) analysis using ESI-TOF was conducted by the mass spectrometry service at the Central Laboratory of Middle East Technical University, Turkey. Masses are presented in units of mass-to-charge ratio (*m*/*z*) as the molecular ion, represented as [M + H]^+^. For the crystal structure determination, a single crystal of molecule **16** was used for data collection on a four-circle Rigaku R-AXIS RAPID-S diffractometer (equipped with a two-dimensional area IP detector). Graphite-monochromated Mo–Kα radiation (λ = 0.71073 Å) and oscillation scans technique with Δ*w* = 5° for one image were used for data collection. The lattice parameters were determined by the least-squares methods based on all reflections with *F*^2^ > 2σ(*F*^2^). Integration of the intensities, correction for Lorentz and polarization effects and cell refinement was performed using CrystalClear (Rigaku/MSC Inc., 2005) software.^46^ The structures were solved by direct methods using SHELXS-2013,^47^ which allowed location of most of the heaviest atoms, with the remaining non-hydrogen atoms being located from difference Fourier maps calculated from successive full-matrix least-squares refinement cycles on *F*^2^ using SHELXL-2013,^47^ All non-hydrogen atoms were refined using anisotropic displacement parameters. The hydrogen atoms were assigned common isotropic displacement factors and included in the final refinement by using geometrical restraints. The final difference Fourier maps showed no peaks of chemical significance. *Crystal data for***16**: C_17_H_14_N_2_O_2_, H_2_O_,_ crystal system, space group: monoclinic, *P*2_1_/*c*; (no:14); unit cell dimensions: *a* = 16.848(2), *b* = 11.6048(15), *c* = 15.483(2) Å, α= 90, β = 102.252(4), γ = 90°; volume; 2958.2(6) Å^3^; calculated density: 1.331 g/cm^3^; absorption coefficient: 0.093 mm^–1^; *F*(000): 1248; θ-range for data collection 3.0–28.4°; refinement method: full-matrix least-squares on *F*^2^; data/parameters: 4433/403; goodness-of-fit on *F*^2^: 1.143; Data completeness; 0.99, final *R*-indices [*I* > 2σ (I)]: *R*_1_ = 0.073, *wR*_2_ = 0.233; largest diff. peak and hole: 0.301 and −0.345 eÅ^–3^. CCDC-2388469 (**16**) contains the supplementary crystallographic data for the structure. These data are provided free of charge via the joint CCDC/FIZ Karlsruhe deposition service www.ccdc.cam.ac.uk/structures.

#### Synthesis of 2-(2-((3-(4-Methoxyphenyl)prop-2-yn-1-yl)oxy)phenyl)-1*H*-benzo[*d*]imidazole (**8b**)

Aldehyde **7b** (708 mg, 2.66 mmol) was dissolved in DMF (8 mL). To this solution were added *o*-phenylenediamine (**4**) (288 mg, 2.66 mmol) and *p*-TsOH (92 mg, 0.53 mmol) at room temperature, and the resulting mixture was heated at 80 °C in an oil bath for 2–3 h. The reaction was monitored by TLC. After the completion of the reaction, Na_2_CO_3_ (56 mg, 0.53 mmol) was dissolved in water (100 mL) and was added to this solution dropwise. EtOAc (20 mL) was added and then extracted with water (2 × 20 mL). The combined organic extracts were dried over MgSO_4_. Removal of the solvent gave the crude product, which was then purified by column chromatography eluting with hexane/EtOAc (3:1) to give the target product **8b**. A brown solid; 358 mg, 38% yield; CC: (SiO_2_; 1:10 EtOAc: hexanes); *R*_f_ = 0.47 (SiO_2_; 1:1 EtOAc: hexanes); mp 154–156 °C; ^1^H NMR (400 MHz, CDCl_3_, 298 K) δ = 8.62 (dd, *J* = 7.8 Hz, 1.7 Hz, 1H), 7.50 (br. s, 2H), 7.48–7.41 (m, 1H), 7.38 (quasi d, *J* = 8.8 Hz, 2H), 7.30–7.21 (m, 4H), 7.19 (t, *J* = 7.6 Hz, 1H), 6.85 (quasi d, *J* = 8.8 Hz, 2H), 5.14 (s, 2H), 3.81 ppm (s, 3H); ^13^C{^1^H} NMR (100 MHz, CDCl_3_, 298 K): δ = 160.3, 155.3, 149.7, 133.5, 131.3, 130.5, 122.8, 122.6, 118.6, 114.2, 113.8, 113.3, 88.7, 81.6, 58.1, 55.4 ppm (16 out of 18 signals expected); HRMS (ESI-TOF) *m*/*z*: [M + H]^+^ calcd for C_23_H_19_N_2_O_2_^+^ 355.1447; found: 355.1450.

#### General Procedure of Cyclization with NaH

To a solution of benzimidazole derivative (1.51 mmol) in DMF (10 mL) was added NaH (60% suspension in oil) (3.02 mmol), and the resulting mixture was stirred at room temperature for 20 h. After completion of the reaction (monitored by TLC), water (10 mL) was added to the reaction medium. The mixture was extracted with EtOAc (2 × 10 mL), and organic extracts were washed with water (5 × 20 mL) and dried over MgSO_4_ and evaporated. The residue was purified by column chromatography.

#### (*Z*)-7-Benzylidene-6,7-dihydrobenzo[*f*]benzo[4,5]imidazo[1,2-*d*][1,4]oxazepane (*Z*)-**9a**

A yellow solid; 245 mg, 50%; CC: (SiO_2_; 1:10 EtOAc: hexanes); *R*_f_ = 0.52 (SiO_2_; 1:4 EtOAc: hexanes); mp 119–121 °C; ^1^H NMR (400 MHz, CDCl_3_, 298 K) δ = 8.72 (d, *J* = 8.1 Hz, 1H), 7.93–7.87 (m, 1H), 7.74–7.65 (m, 1H), 7.54–7.45 (m, 4H), 7.40 (dd, *J* = 10.7, 4.5 Hz, 2H), 7.38–7.33 (m, 2H), 7.23–7.15 (m, 3H), 4.99 ppm (s, 2H); ^13^C{^1^H} NMR (100 MHz, CDCl_3_, 298 K) δ = 156.1, 150.4, 143.5, 134.6, 133.9, 132.0, 131.8, 131.7, 129.1, 128.9, 128.7, 127.3, 126.5, 123.4, 122.7, 120.8, 120.1, 117.5, 110.8, 68.8 ppm; HRMS (ESI-TOF) *m*/*z*: [M + H]^+^ calcd for C_22_H_17_N_2_O^+^ 325.1341; found: 325.1342.

#### (*E*)-7-Benzylidene-6,7-dihydrobenzo[*f*]benzo[4,5]imidazo[1,2-*d*][1,4]oxazepane (*E*)-**9a**

A white solid; 49 mg, 10%; CC: (SiO_2_; 1:10 EtOAc: hexanes); *R*_f_ = 0.42 (SiO_2_; 1:4 EtOAc: hexanes); mp 130–132 °C; ^1^H NMR (400 MHz, CDCl_3_, 298 K) δ = 8.65 (dd, *J* = 8.0, 1.6 Hz, 1H), 7.84 (d, *J* = 8.0 Hz, 1H), 7.47–7.38 (m, 1H), 7.25–7.18 (m, 2H), 7.16–7.06 (m, 4H), 7.02–6.94 (m, 3H), 6.83 (s, 1H), 6.66 (d, *J* = 8.1 Hz, 1H), 5.11 (br. s, 1H), 4.67 ppm (br. s, 1H); ^13^C{^1^H} NMR (100 MHz, CDCl_3_, 298 K): δ = 155.6, 153.8, 150.5, 133.2, 132.5, 132.2, 132.1, 129.4, 129.1, 129.0, 128.6, 127.7, 123.3, 123.0, 122.5, 121.0, 119.8, 116.8, 112.4, 74.8 ppm.

#### (*Z*)-7-(4-Methoxybenzylidene)-6,7-dihydrobenzo[*f*]benzo[4,5]imidazo[1,2-*d*][1,4]oxazepane (*Z*)-**9b**

A pale yellow solid; 214 mg, 40%; CC: (SiO_2_; 1:10 EtOAc: hexanes); *R*_f_ = 0.42 (SiO_2_; 1:4 EtOAc: hexanes); mp 133–135 °C; ^1^H NMR (400 MHz, CDCl_3_, 298 K) δ = 8.69 (d, *J* = 8.0 Hz, 1H), 7.93–7.86 (m, 1H), 7.71–7.64 (m, 1H), 7.47 (quasi d, *J* = 8.7 Hz, 2H), 7.44–7.39 (m, 1H), 7.38–7.33 (m, 2H), 7.23–7.13 (m, 3H), 7.02 (quasi d, *J* = 8.7 Hz, 2H), 5.02 (s, 2H), 3.88 ppm (s, 3H); ^13^C{^1^H} NMR (100 MHz, CDCl_3_, 298 K) δ = 160.2, 156.2, 150.7, 143.4, 134.7, 132.0, 131.9, 130.7, 130.3, 127.6, 126.3, 123.4, 123.3, 122.9, 120.9, 120.0, 117.6, 114.5, 110.9, 69.2, 55.5 ppm; HRMS (ESI-TOF) *m*/*z*: [M + H]^+^ calcd for C_23_H_19_N_2_O_2_^+^ 355.1447; found: 355.1448.

#### (*E*)-7-(4-Methoxybenzylidene)-6,7-dihydrobenzo[*f*]benzo[4,5]imidazo[1,2-*d*][1,4]oxazepane (*E*)-**9b**

A yellow solid; 80 mg, 15%; CC: (SiO_2_; 1:10 EtOAc: hexanes); *R*_f_ = 0.35 (SiO_2_; 1:4 EtOAc: hexanes); mp 186–188 °C; ^1^H NMR (400 MHz, CDCl_3_, 298 K) δ = 8.54 (d, *J* = 8.0 Hz, 1H); 7.76 (d, *J* = 8.1 Hz, 1H), 7.33 (t, *J* = 7.6 Hz, 1H), 7.17 (t, *J* = 8.3 Hz, 1H), 7.10 (t, *J* = 7.6 Hz, 1H), 7.03 (d, *J* = 8.3 Hz, 1H), 6.93 (t, *J* = 7.6 Hz, 1H), 6.85 (quasi d, *J* = 8.7 Hz, 2H), 6.68 (s, 1H), 6.64 (d, *J* = 8.2 Hz, 1H), 6.54 (quasi d, *J* = 8.7 Hz, 2H), 5.00 (br. s, 1H), 4.55 (br. s, 1H), 3.62 ppm (s, 3H); ^13^C{^1^H} NMR (100 MHz, CDCl_3_, 298 K) δ = 160.0, 155.5, 150.7, 143.7, 132.7, 132.2, 132.0, 130.8, 127.5, 127.4, 125.6, 123.2, 123.0, 122.3, 120.9, 119.8, 116.8, 114.0, 112.5, 74.9, 55.3 ppm; HRMS (ESI-TOF) *m*/*z*: [M + H]^+^ calcd for C_23_H_19_N_2_O_2_^+^ 355.1447, found: 355.1444.

#### Synthesis of 7-(4-Nitrobenzylidene)-6,7-dihydrobenzo[*f*]benzo[4,5]imidazo [1,2-*d*][1,4]oxazepine (*Z*)-**9c**

Aldehyde (748 mg, 2.66 mmol) was dissolved in DMF (8 mL). To this solution, *o*-phenylenediamine (288 mg, 2.66 mmol) and *p*-TsOH (92 mg, 0.53 mmol) were added at room temperature, and the resulting mixture was heated at 80 °C in an oil bath for 2–3 h. The reaction was monitored by TLC. After the completion of the reaction, Na_2_CO_3_ (56 mg, 0.53 mmol) was dissolved in water (100 mL) and was added to this solution dropwise. EtOAc (20 mL) was added and then extracted with water (2 × 20 mL). The combined organic extracts were dried over MgSO_4_. Removal of the solvent gave the crude product, which was then purified by column chromatography eluting with hexane/EtOAc (10:1) to give product (*E*)*-***9c**. A pale yellow solid; 295 mg, 30%; CC: (SiO_2_; 1:10 EtOAc: hexanes); *R*_f_ = 0.37 (SiO_2_; 1:4 EtOAc: hexanes); mp 195–197 °C; ^1^H NMR (400 MHz, CDCl_3_) δ = 8.74 (dd, *J* = 8.1, 1.4 Hz, 1H), 8.35 (quasi d, *J* = 8.7 Hz, 2H), 7.89 (dd, *J* = 6.1, 2.8 Hz, 1H), 7.80–7.65 (m, 3H), 7.44 (t, *J* = 7.7 Hz, 1H), 7.41–7.36 (m, 2H), 7.28–7.15 (m, 3H), 4.98 ppm (s, 2H); ^13^C{^1^H} NMR (100 MHz, CDCl_3_, 298 K) δ = 156.1, 151.0, 150.2, 147.7, 143.5, 140.6, 134.7, 134.3, 132.2, 130.1, 124.3, 124.2, 123.9, 123.8, 123.2, 120.7, 120.4, 117.2, 110.7, 68.4 ppm; HRMS (ESI-TOF) *m*/*z*: [M + H]^+^ calcd for C_22_H_16_N_3_O_3_^+^ 370.1192, found: 370.1189.

#### 7-Methylene-6,7-dihydrobenzo[*f*]benzo[4,5]imidazo[1,2-*d*][1,4]oxazepane (**10a**)

A white solid; 37 mg, 10%; CC: (SiO_2_; 1:10 EtOAc: hexanes); *R*_f_ = 0.49 (SiO_2_; 1:4 EtOAc: hexanes); mp 80–82 °C; ^1^H NMR (400 MHz, CDCl_3_, 298 K) δ = 8.77 (d, *J* = 8.1 Hz, 1H), 7.85 (d, *J* = 7.5 Hz, 1H), 7.61 (d, *J* = 7.4 Hz, 1H), 7.41–7.29 (m, 3H), 7.18 (t, *J* = 7.6 Hz, 1H), 7.10 (d, *J* = 8.2 Hz, 1H), 5.71 (s, 1H), 5.52 (s, 1H), 4.80 ppm (s, 2H); ^13^C{^1^H} NMR (100 MHz, CDCl_3_, 298 K) δ = 156.5, 149.8, 143.6, 138.7, 134.7, 132.1, 131.9, 123.6, 123.0, 120.7, 120.2, 117.7, 111.4, 110.2, 72.8 ppm (15 out of 16 expected signals).

#### 6-Methylbenzo[*f*]benzo[4,5]imidazo[1,2-*d*][1,4]oxazepane (**11a**)

A white solid; 240 mg, 64%; CC: (SiO_2_; 1:10 EtOAc: hexanes); *R*_f_ = 0.38 (SiO_2_; 1:4 EtOAc: hexanes); mp 100–102 °C; ^1^H NMR (400 MHz, CDCl_3_, 298 K) δ = 8.22 (d, *J* = 7.3 Hz, 1H), 7.83 (d, *J* = 8.3 Hz, 1H), 7.45 (t, *J* = 7.7 Hz, 1H), 7.39 (d, *J* = 8.1 Hz, 1H), 7.36–7.27 (m, 3H), 7.13 (d, *J* = 8.1 Hz, 1H), 6.50 (s, 1H), 2.15 ppm (s, 3H); ^13^C{^1^H} NMR (100 MHz, CDCl_3_, 298 K) δ = 158.0, 150.0, 149.0, 142.7, 134.5, 132.4, 130.9, 125.7, 123.6, 123.5, 123.0, 121.4, 120.1, 109.6, 108.5, 18.6 ppm; HRMS (ESI-TOF) *m*/*z*: [M + H]^+^ calcd for C_16_H_13_N_2_O^+^ 249.1028; found: 249.1028.

#### 2-Bromo-7-methylene-6,7-dihydrobenzo[*f*]benzo[4,5]imidazo[1,2-*d*][1,4]oxazepane (**10b**)

A white solid; 49 mg, 10%; CC: (SiO_2_; 1:15 EtOAc: hexanes); *R*_f_ = 0.57 (SiO_2_; 1:4 EtOAc: hexanes); mp 175–177 °C; ^1^H NMR (400 MHz, CDCl_3_, 298 K) δ = 8.93 (d, *J* = 2.4 Hz, 1H), 7.84 (dd, *J* = 6.3, 2.4 Hz, 1H), 7.60 (dd, *J* = 6.3, 1.9 Hz, 1H), 7.44 (dd, *J* = 8.7, 2.4 Hz, 1H), 7.38–7.28 (m, 2H), 6.97 (d, *J* = 8.7 Hz, 1H), 5.74 (s, 1H), 5.54 (s, 1H), 4.77 ppm (s, 2H); ^13^C{^1^H} NMR (100 MHz, CDCl_3_, 298 K): δ = 155.5, 148.3, 143.4, 138.2, 134.7, 134.5, 134.1, 124.0, 123.9, 122.5, 120.3, 119.2, 115.4, 111.4, 110.8, 72.7 ppm.

#### 2-Bromo-6-methylbenzo[*f*]benzo[4,5]imidazo[1,2-*d*][1,4]oxazepine (**11b**)

A white solid; 163 mg, 33%; CC: (SiO_2_; 1:15 EtOAc: hexanes); *R*_f_ = 0.53 (SiO_2_; 1:4 EtOAc: hexanes); mp 135–137 °C; ^1^H NMR (400 MHz, CDCl_3_, 298 K) δ = 8.37 (s, 1H); 7.84 (d, *J* = 5.6 Hz, 1H), 7.57–7.51 (m, 1H), 7.42–7.30 (m, 3H), 7.02 (d, *J* = 8.6 Hz, 1H), 6.50 (s, 1H), 2.15 ppm (s, 3H); ^13^C{^1^H} NMR (100 MHz, CDCl_3_, 298 K) δ = 156.8, 148.7, 148.5, 142.6, 134.9, 134.5, 133.1, 124.6, 124.0, 123.7, 123.1, 120.3, 118.4, 109.7, 108.4, 18.6 ppm; HRMS (ESI-TOF) *m*/*z*: [M + H]^+^ calcd for C_16_H_12_BrN_2_O^+^ 327.0133; found: 327.0132.

#### 4-Methoxy-7-methylene-6,7-dihydrobenzo[*f*]benzo[4,5]imidazo[1,2-*d*][1,4]oxazepane (**10c**)

A pale yellow solid; 67 mg,16%; CC: (SiO_2_; 1:15 EtOAc: hexanes); *R*_f_ = 0.24 (SiO_2_; 1:4 EtOAc: hexanes); mp 161–163 °C; ^1^H NMR (400 MHz, CDCl_3_, 298 K) δ = 8.27 (dd, *J* = 8.3, 1.5 Hz, 1H), 7.88–7.80 (m, 1H), 7.63–7.56 (m, 1H), 7.37–7.29 (m, 2H), 7.14 (t, *J* = 8.1 Hz, 1H), 7.00 (dd, *J* = 7.9, 1.2 Hz, 1H), 5.68 (s, 1H), 5.51 (s, 1H), 4.93 (s, 2H), 3.94 ppm (s, 3H); ^13^C{^1^H} NMR (100 MHz, CDCl_3_, 298 K) δ = 151.0, 149.8, 146.4, 143.6, 138.6, 134.8, 123.7, 123.6, 123.3, 123.0, 120.2, 119.6, 113.3, 111.3, 110.3, 73.7, 56.4 ppm.

#### 4-Methoxy-6-methylbenzo[*f*]benzo[4,5]imidazo[1,2-*d*][1,4]oxazepane (**11c**)

A white solid; 155 mg, 37%; CC: (SiO_2_; 1:15 EtOAc: hexanes); *R*_f_ = 0.18 (SiO_2_; 1:4 EtOAc: hexanes); mp 135–137 °C; ^1^H NMR (400 MHz, CDCl_3_, 298 K) δ = 7.86–7.80 (m, 1H), 7.76 (dd, *J* = 7.9, 1.3 Hz, 1H), 7.42–7.36 (m, 1H), 7.36–7.28 (m, 2H), 7.22 (t, *J* = 8.0 Hz, 1H), 7.07 (d, *J* = 8.2 Hz, 1H), 6.53 (s, 1H), 3.92 (s, 3H), 2.20 ppm (s, 3H); ^13^C{^1^H} NMR (100 MHz, CDCl_3_, 298 K) δ = 151.5, 150.2, 150.0, 147.4, 142.5, 134.4, 125.7, 124.4, 123.6, 123.5, 122.0, 120.1, 114.7, 109.7, 109.2, 56.4, 18.6 ppm; HRMS (ESI-TOF) *m*/*z*: [M + H]^+^ calcd for C_17_H_15_N_2_O_2_^+^ 279.1134; found: 279.1134.

#### Synthesis of 4-Methoxy-7-methylbenzo[*f*]benzo[4,5]imidazo[1,2-*d*][1,4]oxazepane (**18**)

To a solution of **10c** (20 mg, 0.072 mmol) in DMF (5 mL) was added NaH (60% suspension in oil) (3 mg, 0.144 mmol) and the resulting mixture was heated to 85 °C in an oil bath and stirred for 3 days to afford compound **18**. A yellow solid; 20 mg, 100%; CC: (SiO_2_; 1:1 EtOAc: hexanes); *R*_f_ = 0.18 (SiO_2_; 1:4 EtOAc: hexanes); mp 95–97 °C; ^1^H NMR (400 MHz, CDCl_3_, 298 K) δ = 7.86 (t, *J* = 7.8 Hz, 1H), 7.78 (dd, *J* = 7.9, 1.1 Hz, 1H), 7.58 (d, *J* = 7.5 Hz, 1H), 7.38–7.29 (m, 2H), 7.25 (t, *J* = 8.0 Hz, 1H), 7.07 (d, *J* = 8.1 Hz, 1H), 6.60 (d, *J* = 1.2 Hz, 1H), 3.92 (s, 3H), 2.29 ppm (s, 3H); ^13^C{^1^H} NMR (100 MHz, CDCl_3_, 298 K) δ = 150.8, 150.4, 149.2, 143.2, 137.4, 134.1, 125.8, 125.6, 124.4, 123.5, 123.4, 122.2, 120.4, 114.6, 113.0, 56.4, 15.1 ppm; HRMS (ESI-TOF) *m*/*z*: [M + H]^+^ calcd for C_17_H_15_N_2_O_2_^+^ 279.1134, found: 279.1133.

## Data Availability

The data underlying this study are available in the published article and its Supporting Information.

## Supporting Information Available

Theoretical calculations, HRMS data, copies of ^1^H NMR and ^13^C{^1^H} NMR spectra, and Crystal Information Checkcif Report (PDF)
