# Supplementary material for: Base-Mediated Synthesis of Imidazole-Fused 1,4-Benzoxazepines via 7-exo-dig Cyclizations: Propargyl Group Transformation
Source: J Org Chem. 2025 Mar 26;90(13):4675–89. doi: 10.1021/acs.joc.5c00106 (PMC11976854; doi:10.1021/acs.joc.5c00106)
Supplement: Supplementary file 1 — jo5c00106_si_001.pdf [file jo5c00106_si_001.pdf]

Supporting Information

The Journal of Organic Chemistry

Base-mediated Synthesis of Imidazole-fused 1,4-Benzoxazepines *via* 7-*exo-dig* Cyclizations: Propargyl Group Transformation

Nalan Korkmaz Cokol,[a,†] Fevzi Can Inyurt,[a,†] İpek Öktem,[a] Ertan Sahin,[b] Ozlem Sari,[c] Cagatay Dengiz,[a,*] Metin Balci[a,*]

[a] *Department of Chemistry, Middle East Technical University, 06800 Ankara, Turkey.*

[b] *Atatürk University, Department of Chemistry, 25240 Erzurum, Turkey*

[c] *Network Technologies Department, TÜBİTAK ULAKBİM, TR-06800 Ankara, Turkey*

Corresponding authors.

E-mail address: mbalci@metu.edu.tr (M. Balci); dengizc@metu.edu.tr (C. Dengiz)

Table of Contents

1. ^{1}H and $^{13}C\{1H\}$ NMR spectra **S3**

2. High-Resolution Mass Spectrometry (HR-MS) Data **S17**

3. Theoretical Calculations **S22**

1. ^{1}H and ^{13}C{1H} NMR spectra

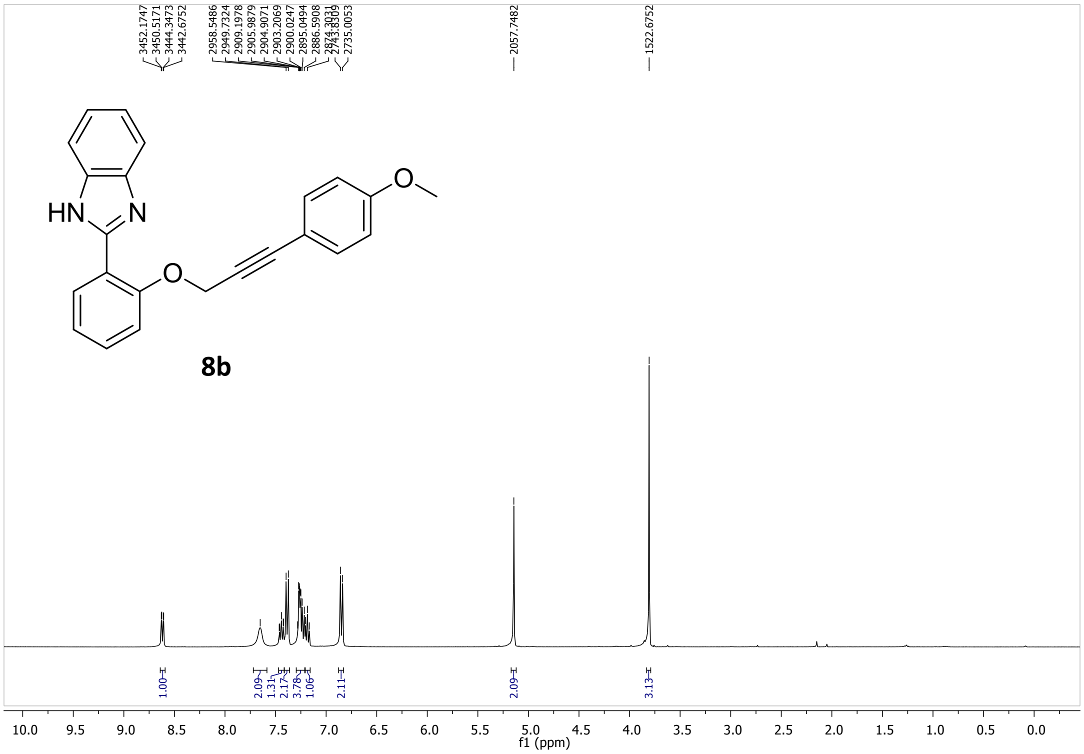

Figure S1. ^{1}H NMR spectrum of **8b** in $CDCl_3$ solution (400 MHz).

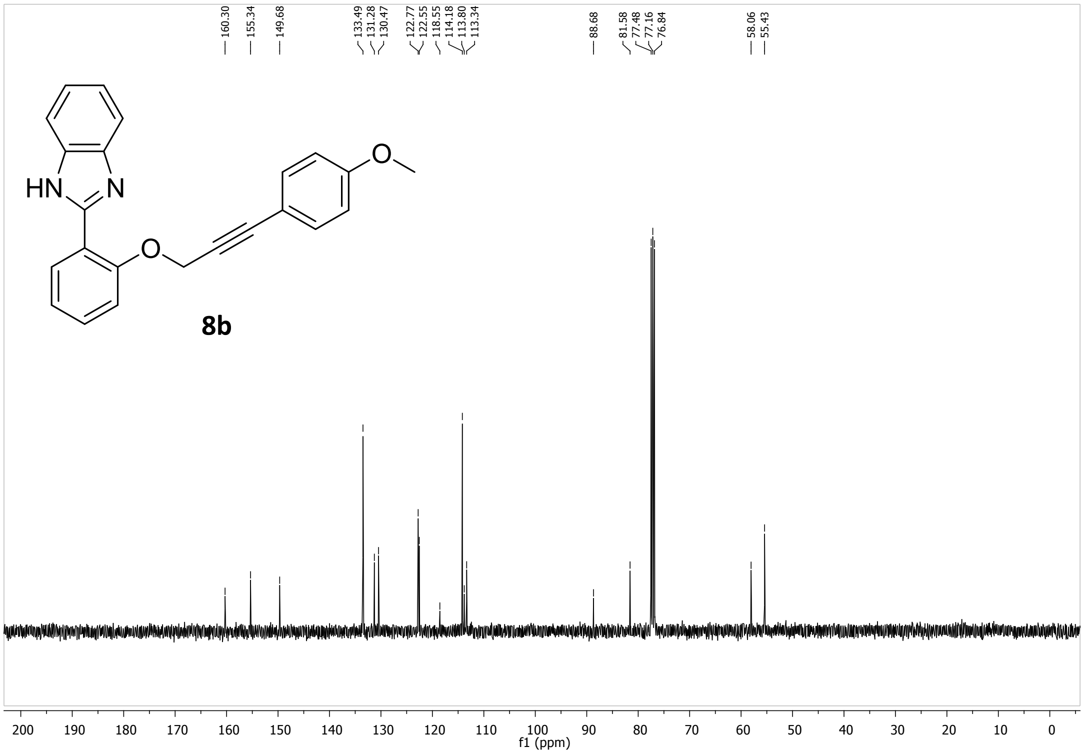

Figure S2. ^{13}C{^{1}H} NMR spectrum of **8b** in $CDCl_3$ solution (100 MHz).

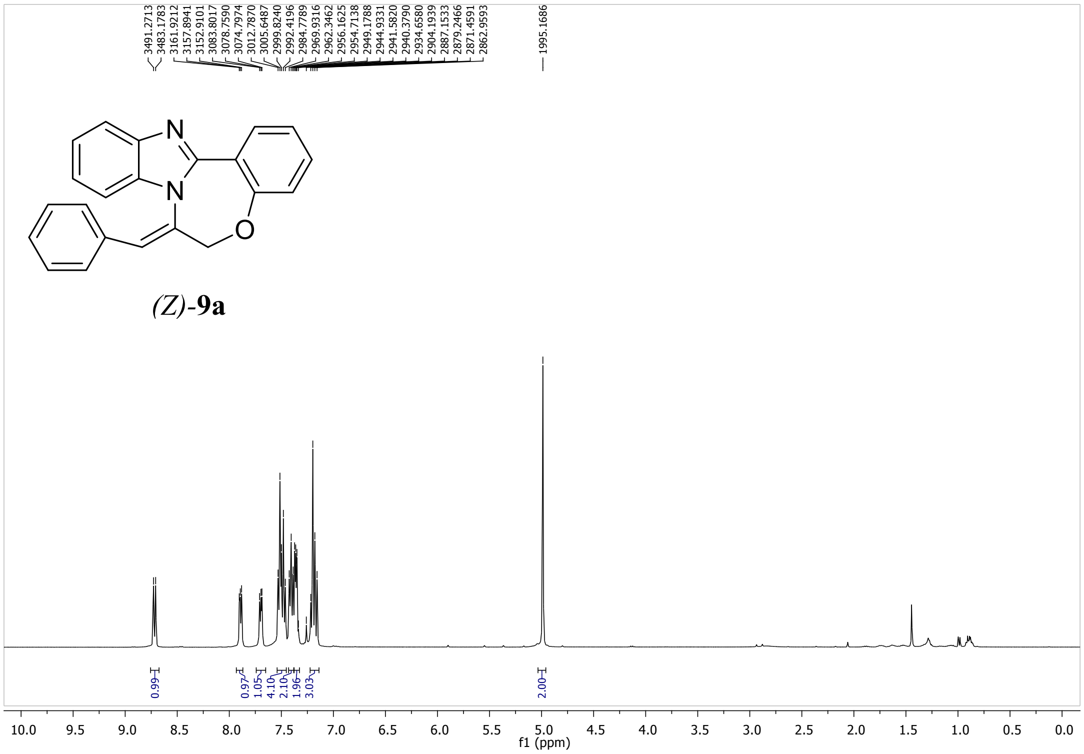

Figure S3. ^{1}H NMR spectrum of *(Z)*-**9a** in $CDCl_3$ solution (400 MHz).

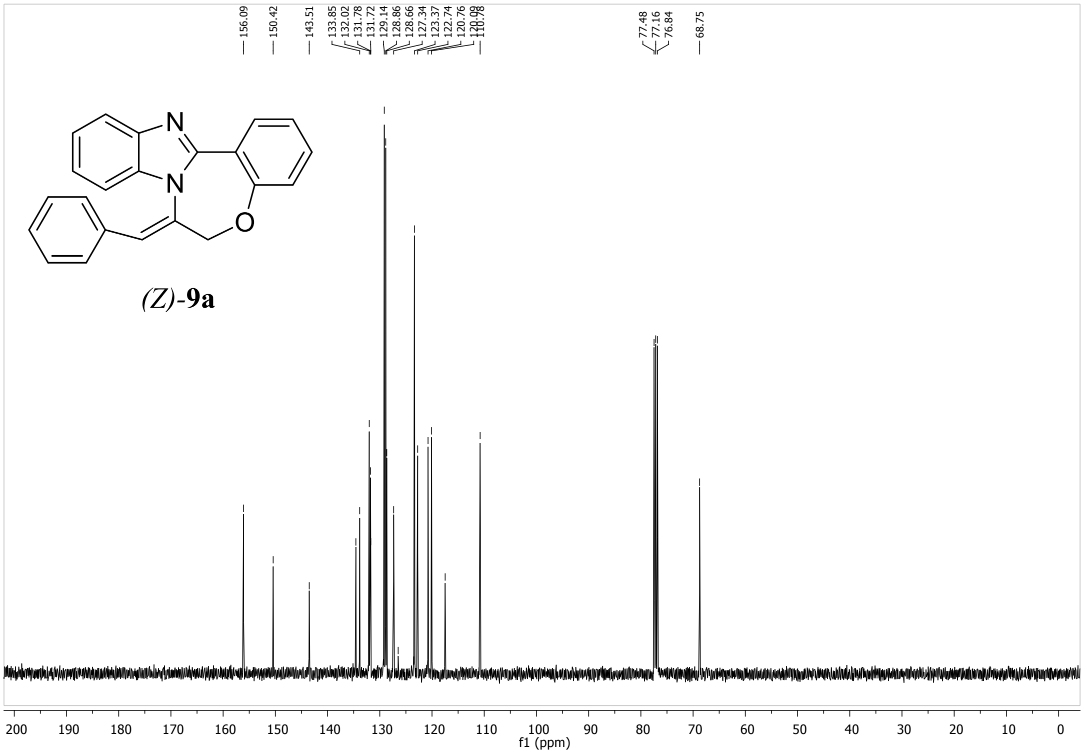

Figure S4. $^{13}C\{^{1}H\}$ NMR spectrum of *(Z)*-**9a** in $CDCl_3$ solution (100 MHz).

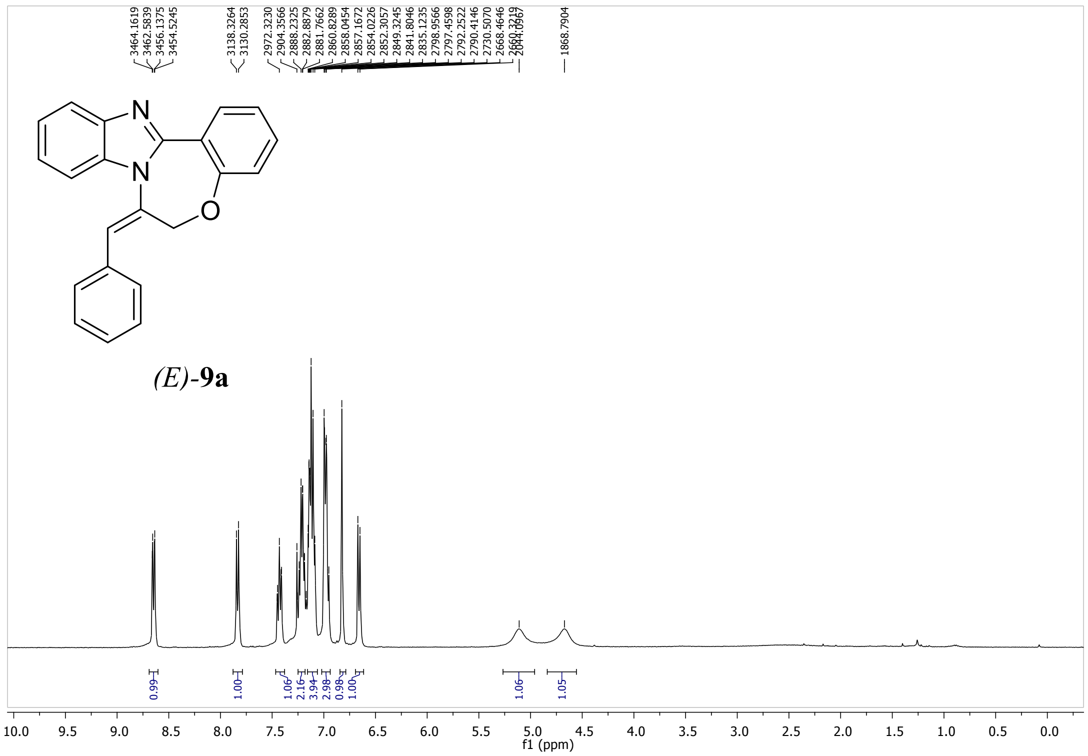

(E)-9a

Figure S5. ^{1}H NMR spectrum of *(E)*-**9a** in $CDCl_3$ solution (400 MHz).

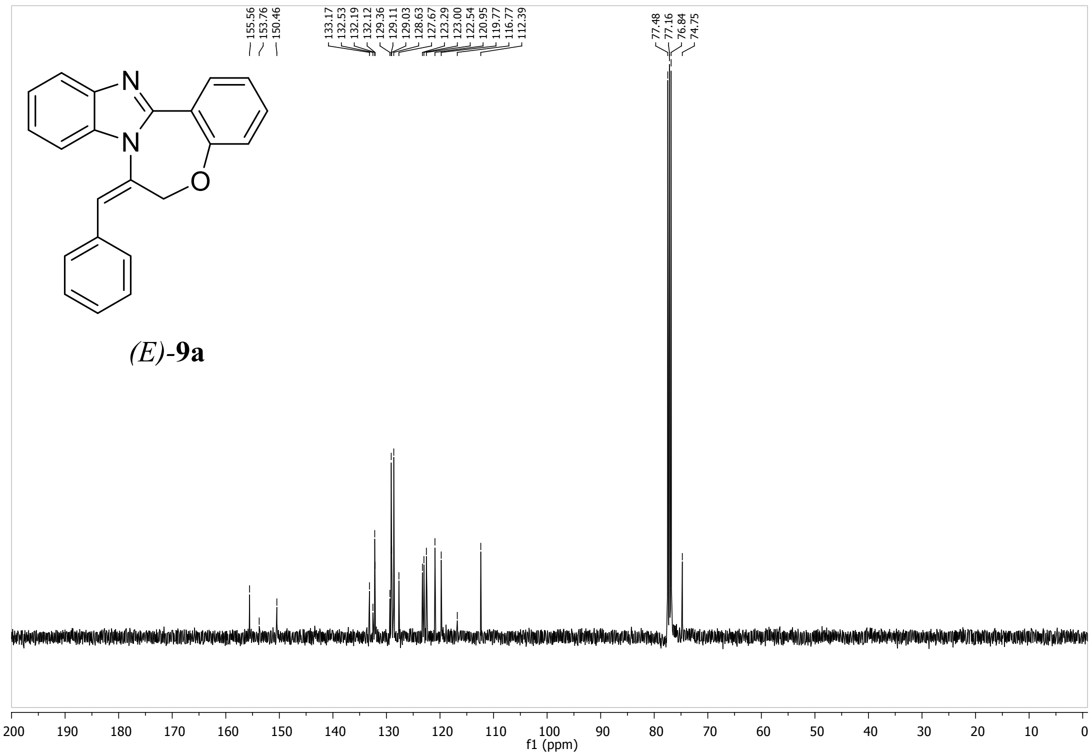

(E)-9a

Figure S6. $^{13}C\{^{1}H\}$ NMR spectrum of *(E)*-**9a** in $CDCl_3$ solution (100 MHz).

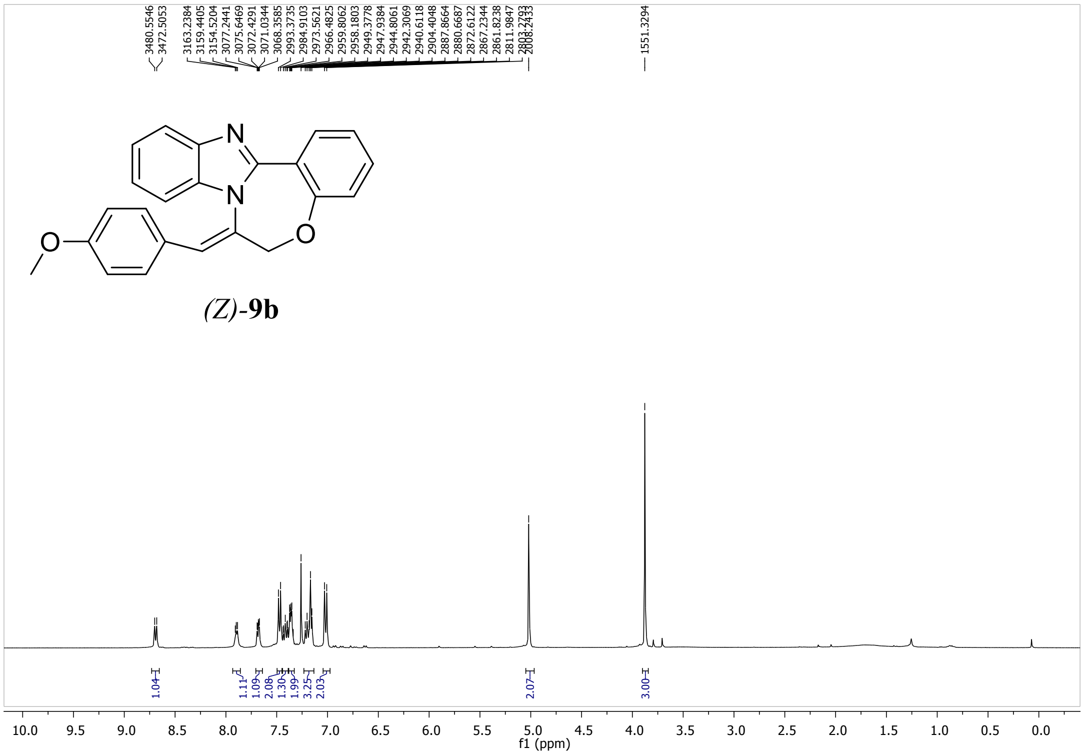

Figure S7. ^{1}H NMR spectrum of *(Z)*-**9b** in $CDCl_3$ solution (400 MHz).

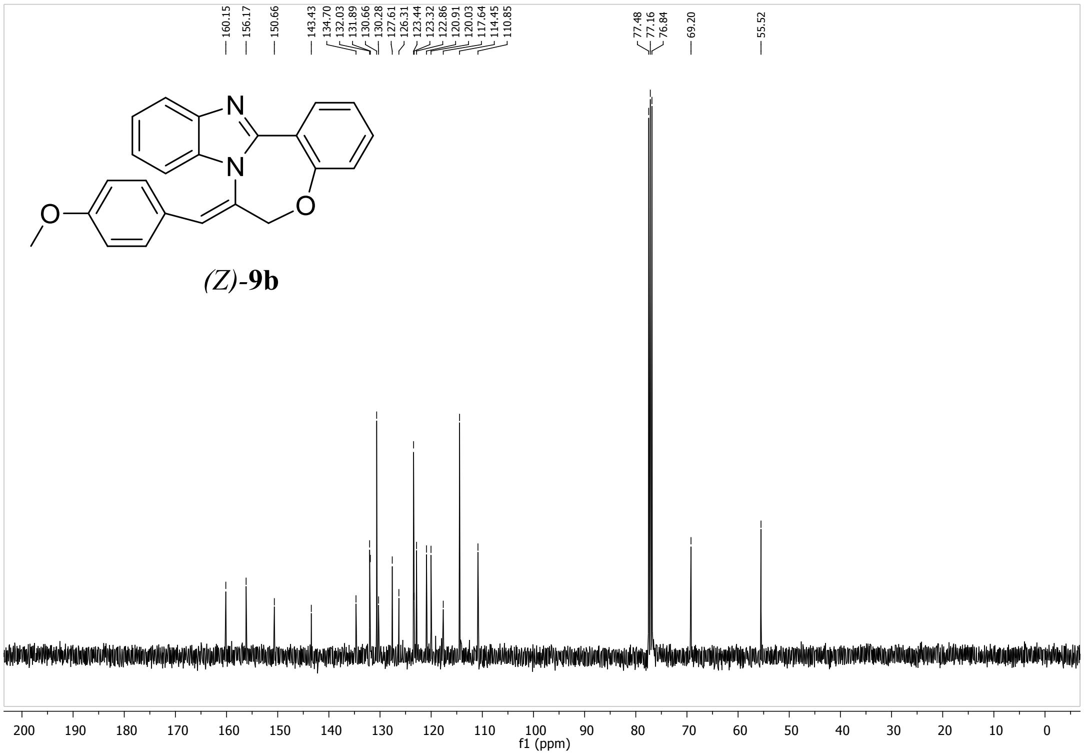

Figure S8. $^{13}C\{^{1}H\}$ NMR spectrum of *(Z)*-**9b** in $CDCl_3$ solution (100 MHz).

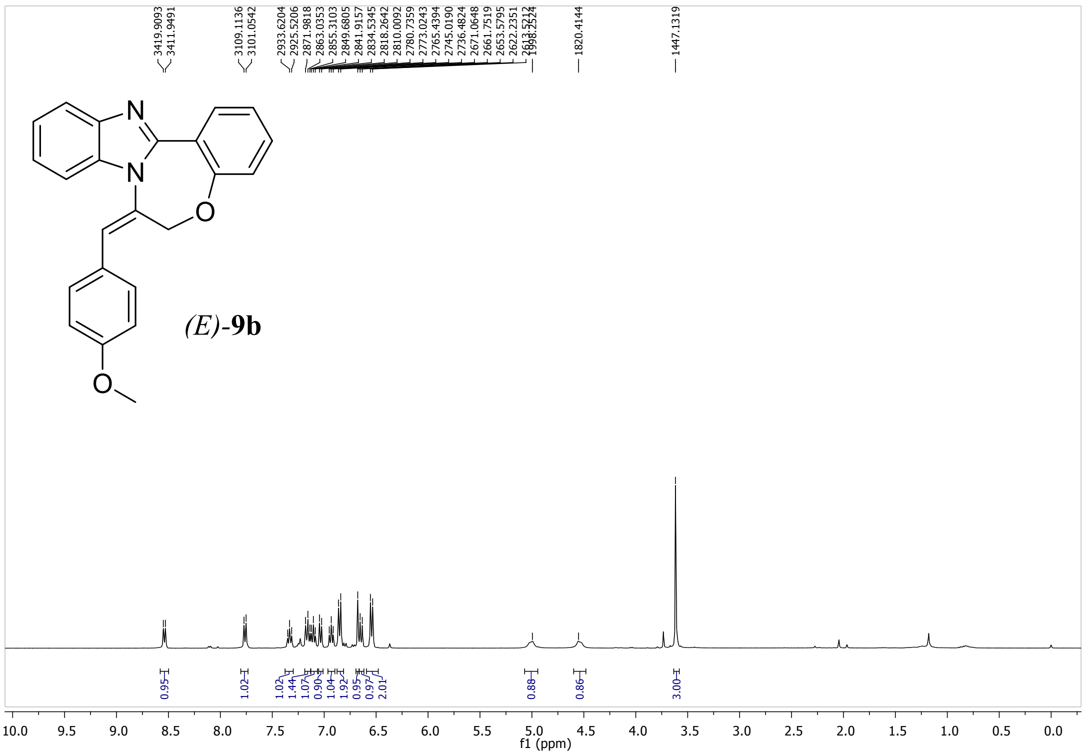

Figure S9. ^{1}H NMR spectrum of *(E)*-**9b** in $CDCl_3$ solution (400 MHz).

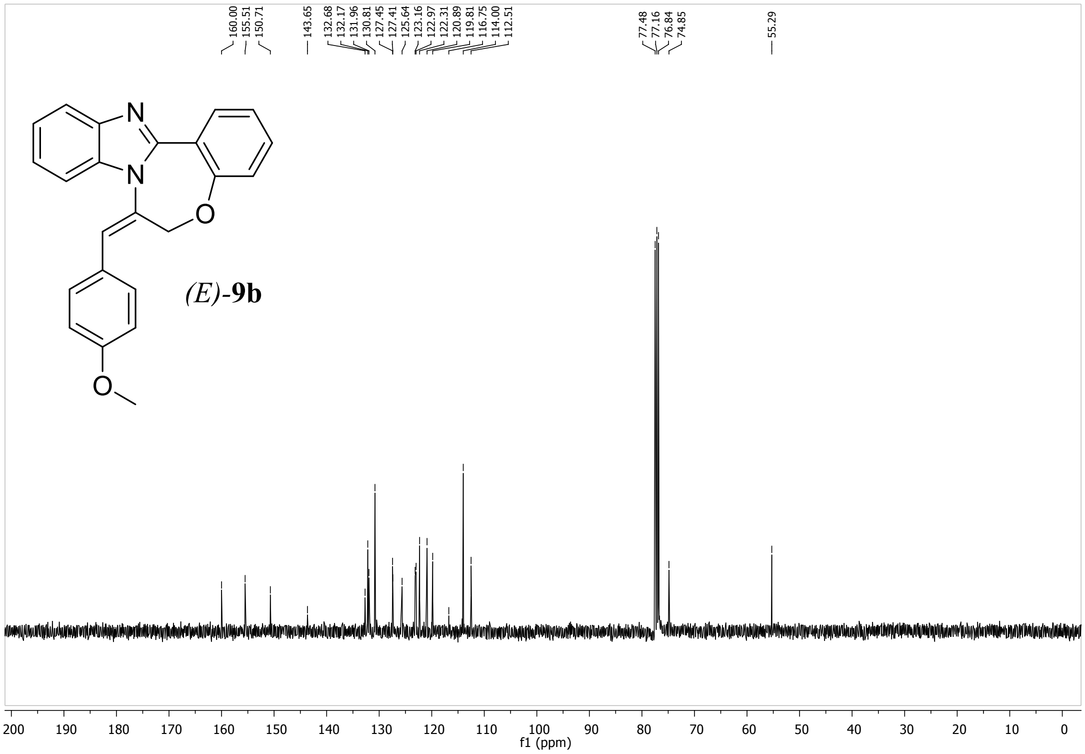

Figure S10. $^{13}C\{^{1}H\}$ NMR spectrum of *(E)*-**9b** in $CDCl_3$ solution (100 MHz).

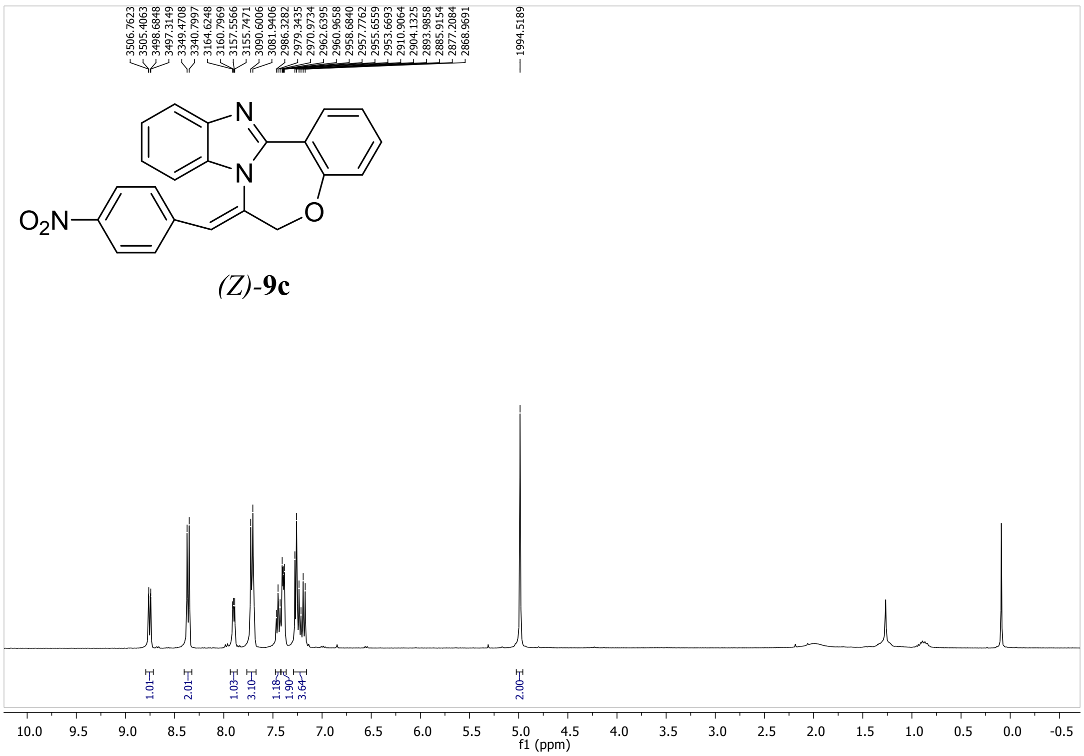

Figure S11. ^{1}H NMR spectrum of *(Z)*-**9c** in $CDCl_3$ solution (400 MHz).

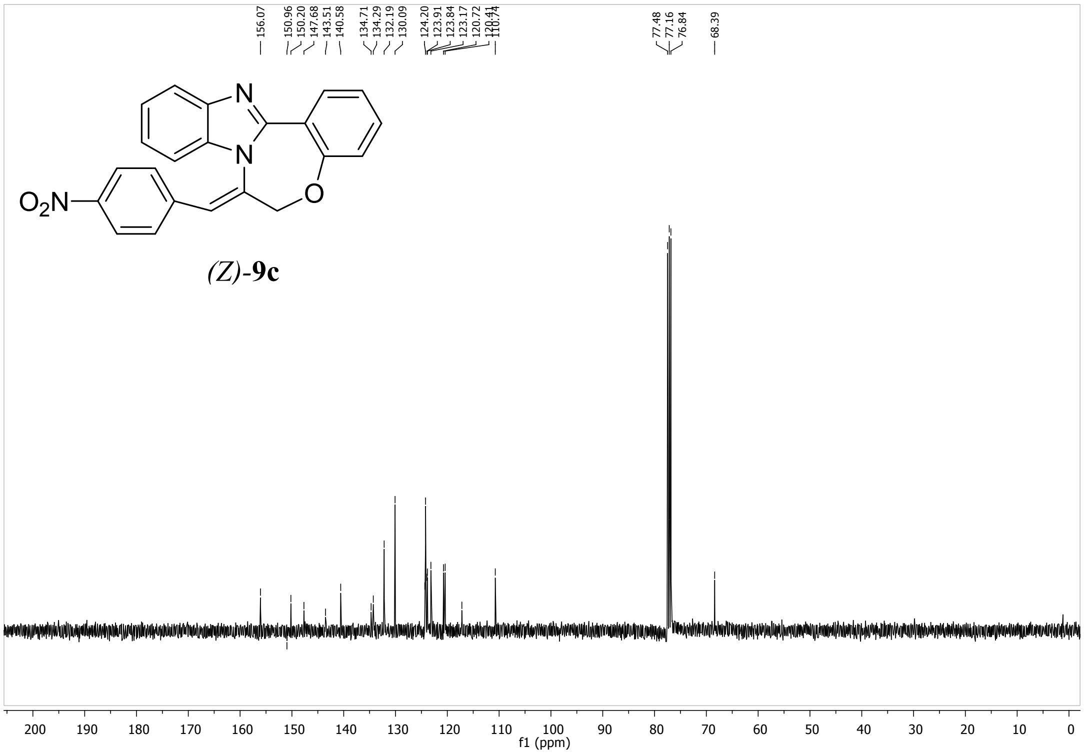

Figure S12. $^{13}C\{^{1}H\}$ NMR spectrum of *(Z)*-**9c** in $CDCl_3$ solution (100 MHz).

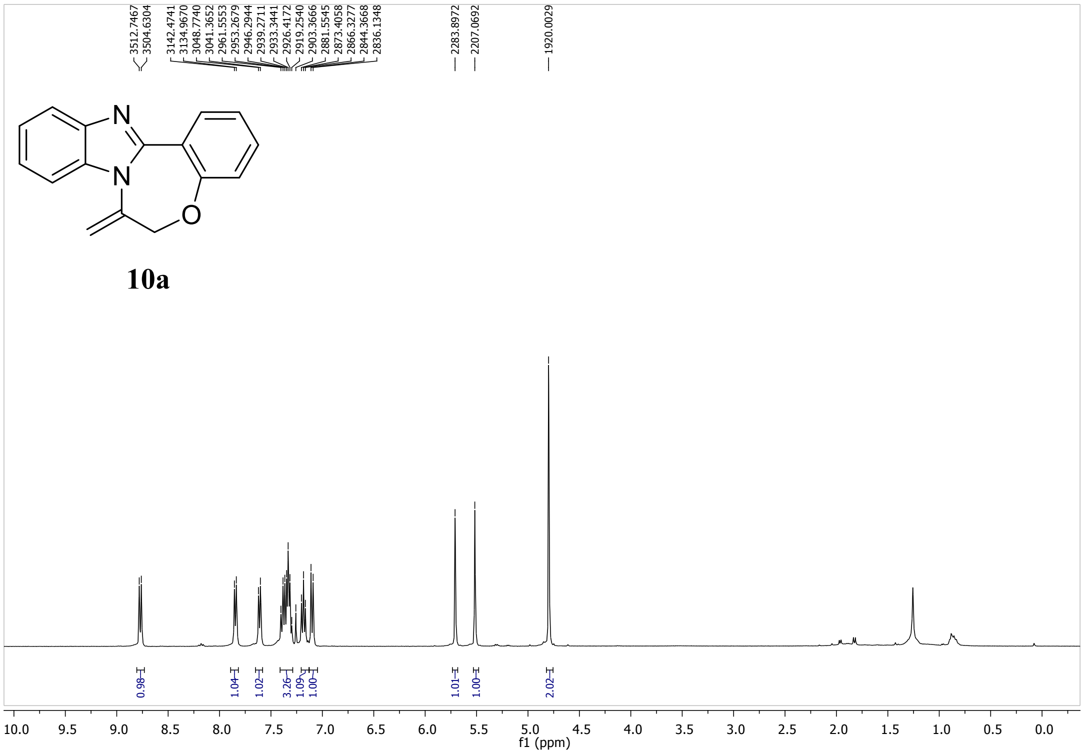

Figure S13. ^{1}H NMR spectrum of **10a** in $CDCl_3$ solution (400 MHz).

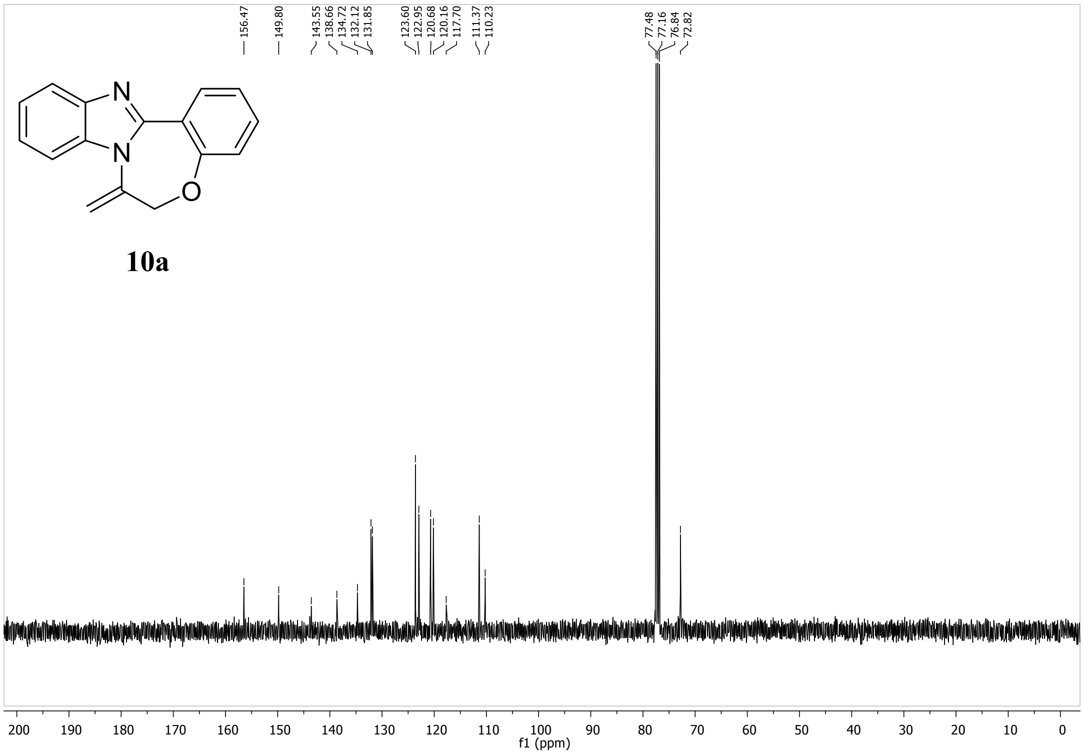

Figure S14. $^{13}C\{^{1}H\}$ NMR spectrum of **10a** in $CDCl_3$ solution (100 MHz).

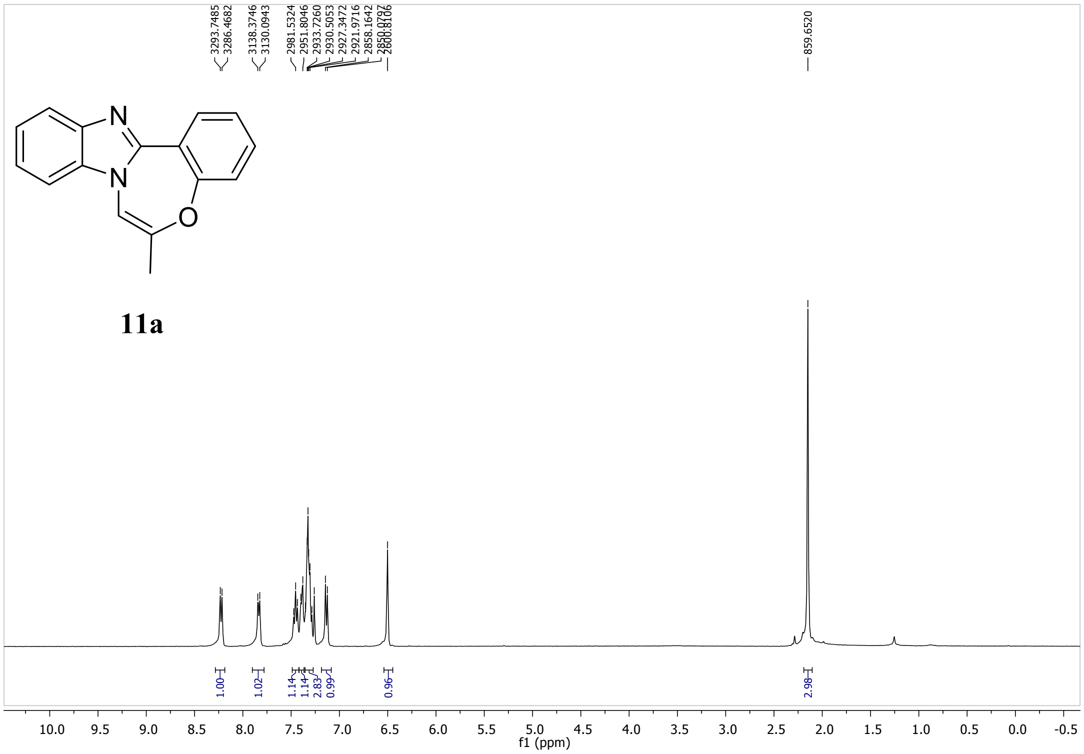

Figure S15. ^{1}H NMR spectrum of **11a** in $CDCl_3$ solution (400 MHz).

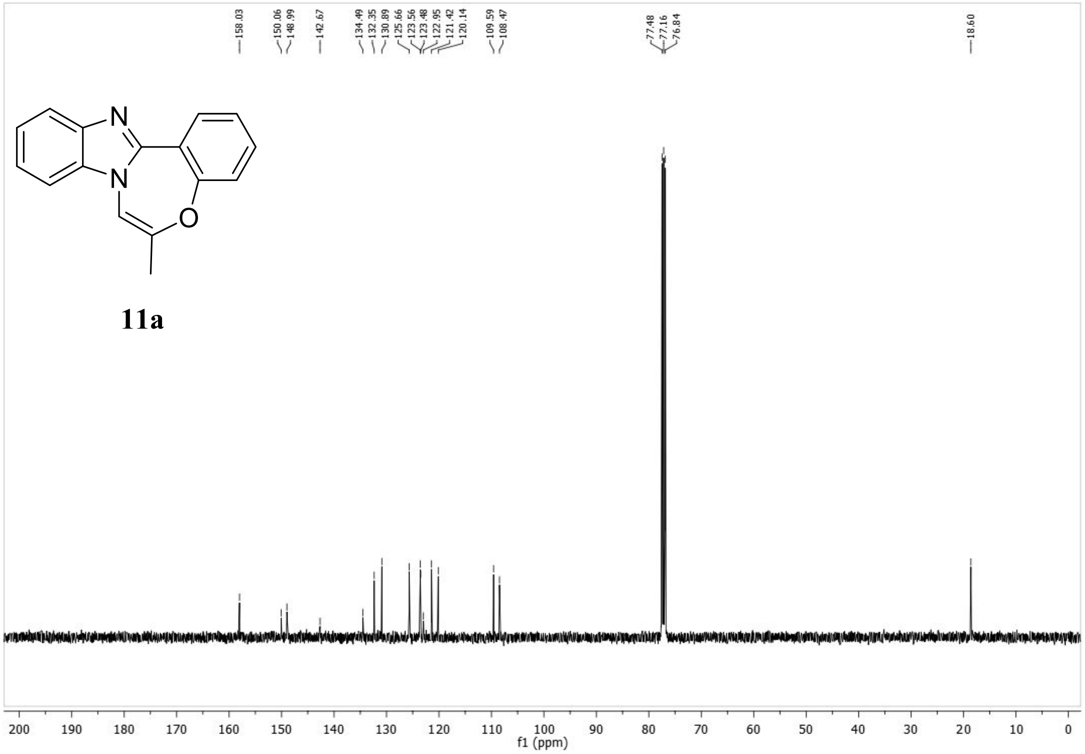

Figure S16. $^{13}C\{^{1}H\}$ NMR spectrum of **11a** in $CDCl_3$ solution (100 MHz).

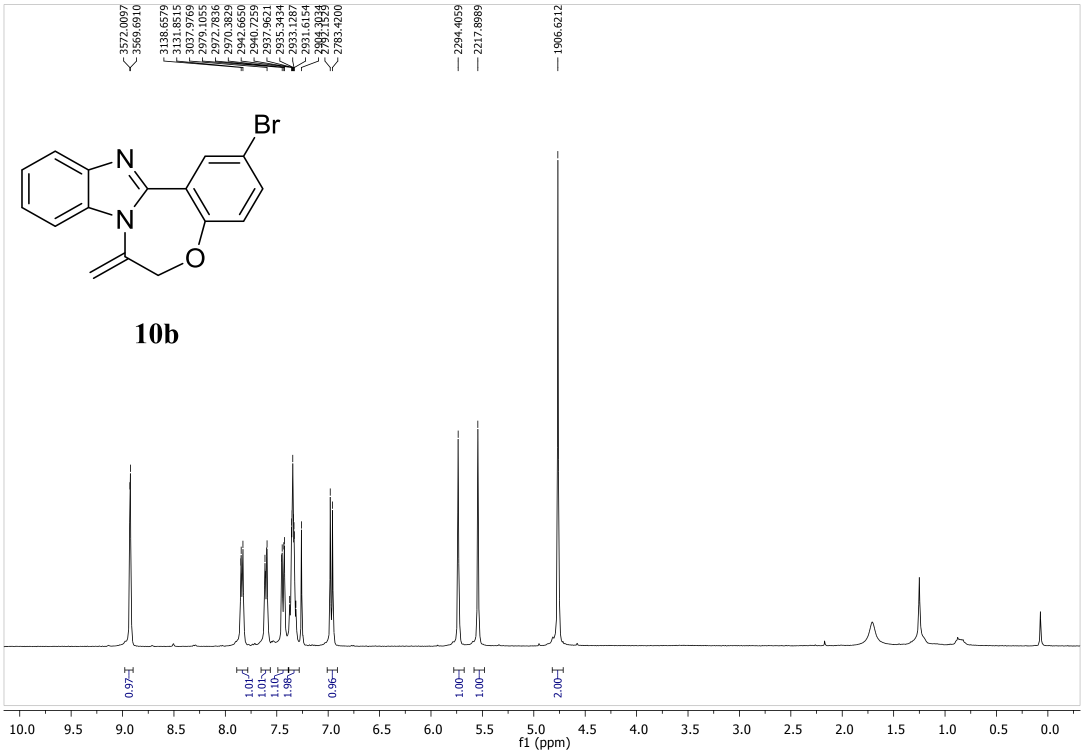

Figure S17. ^{1}H NMR spectrum of **10b** in $CDCl_3$ solution (400 MHz).

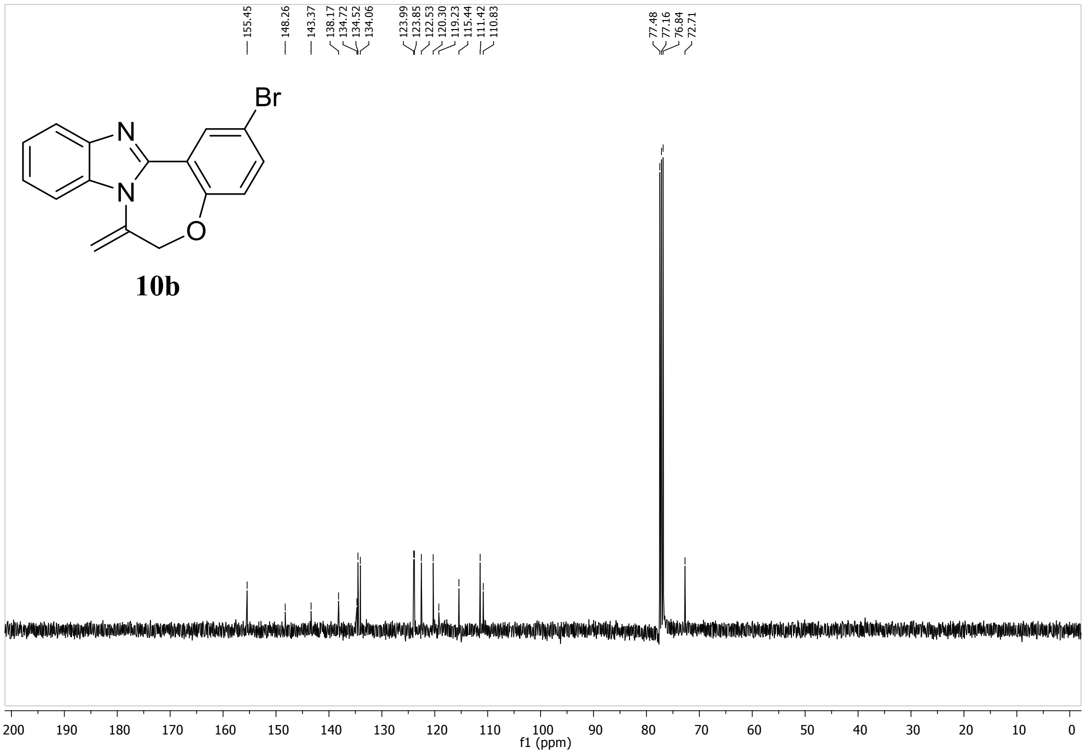

Figure S18. $^{13}C\{^1H\}$ NMR spectrum of **10b** in $CDCl_3$ solution (100 MHz).

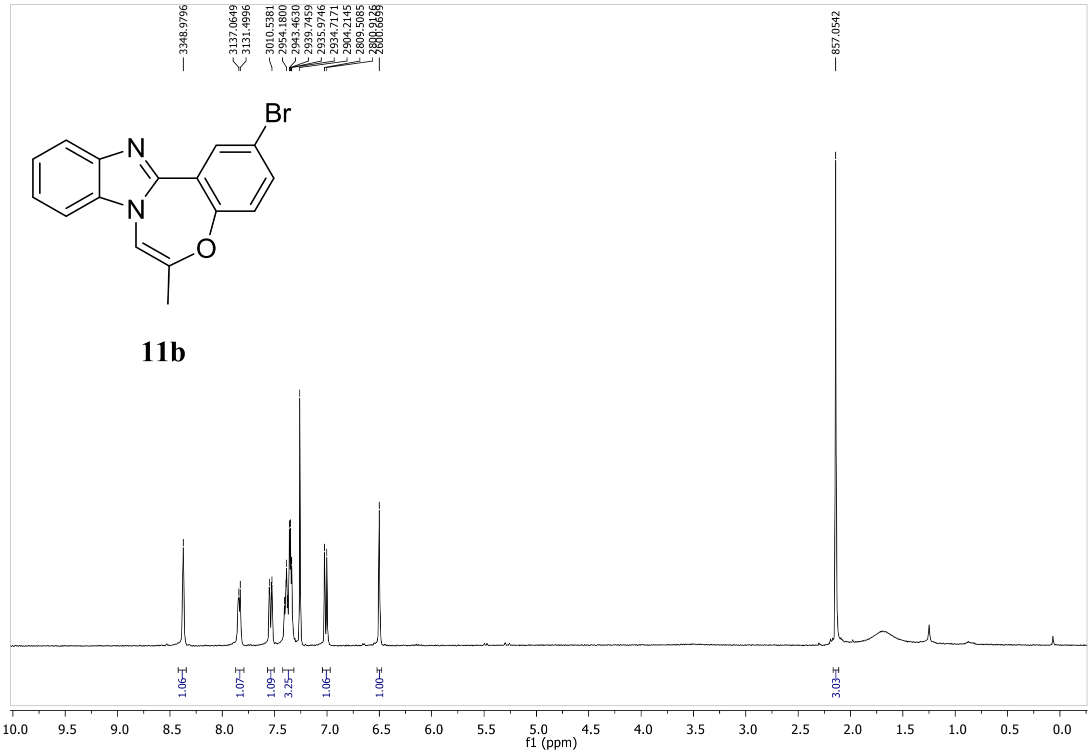

Figure S19. ^{1}H NMR spectrum of **11b** in $CDCl_3$ solution (400 MHz).

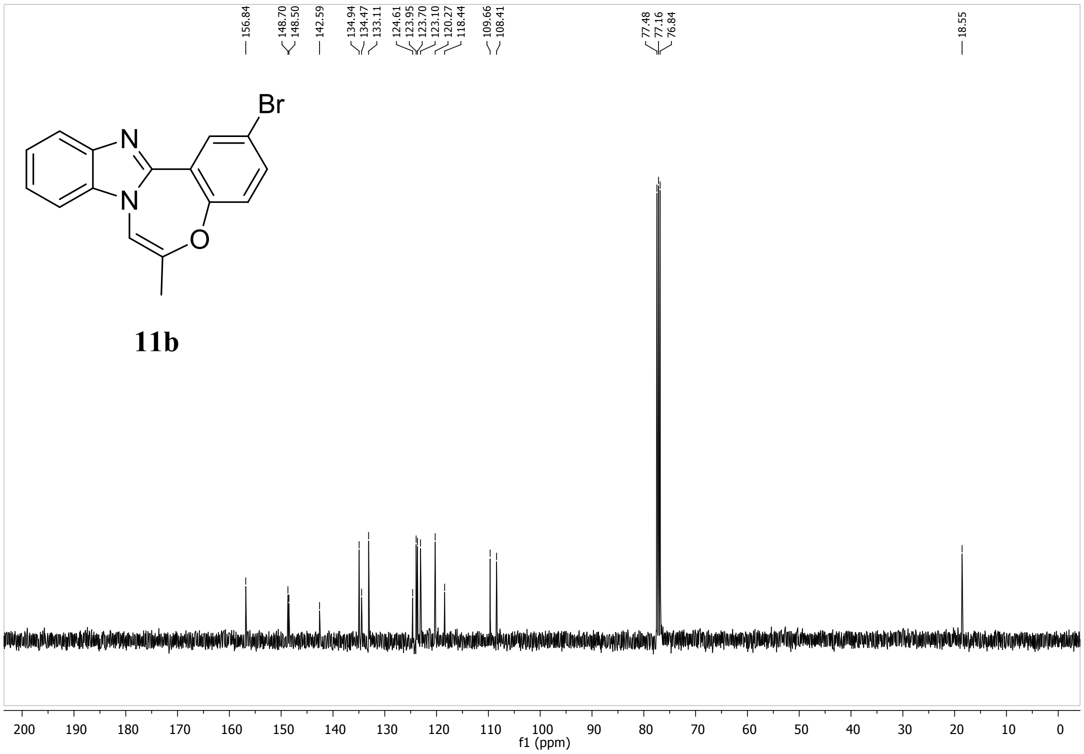

Figure S20. $^{13}C\{^{1}H\}$ NMR spectrum of **11b** in $CDCl_3$ solution (100 MHz).

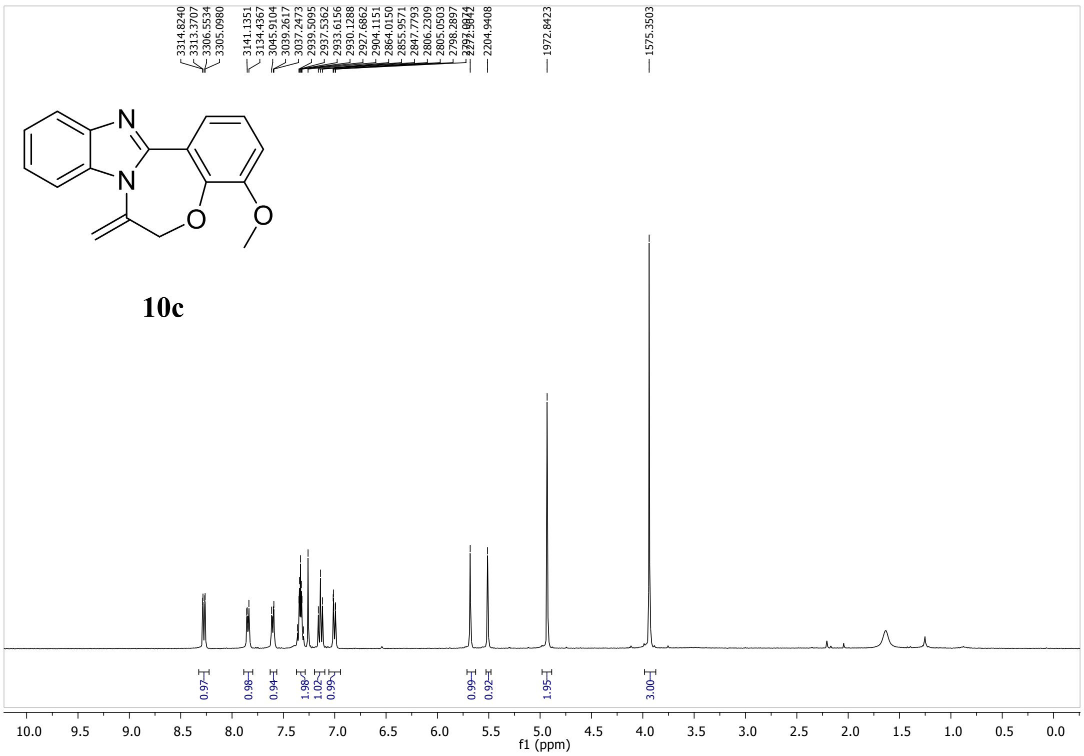

Figure S21. ^{1}H NMR spectrum of **10c** in $CDCl_3$ solution (400 MHz).

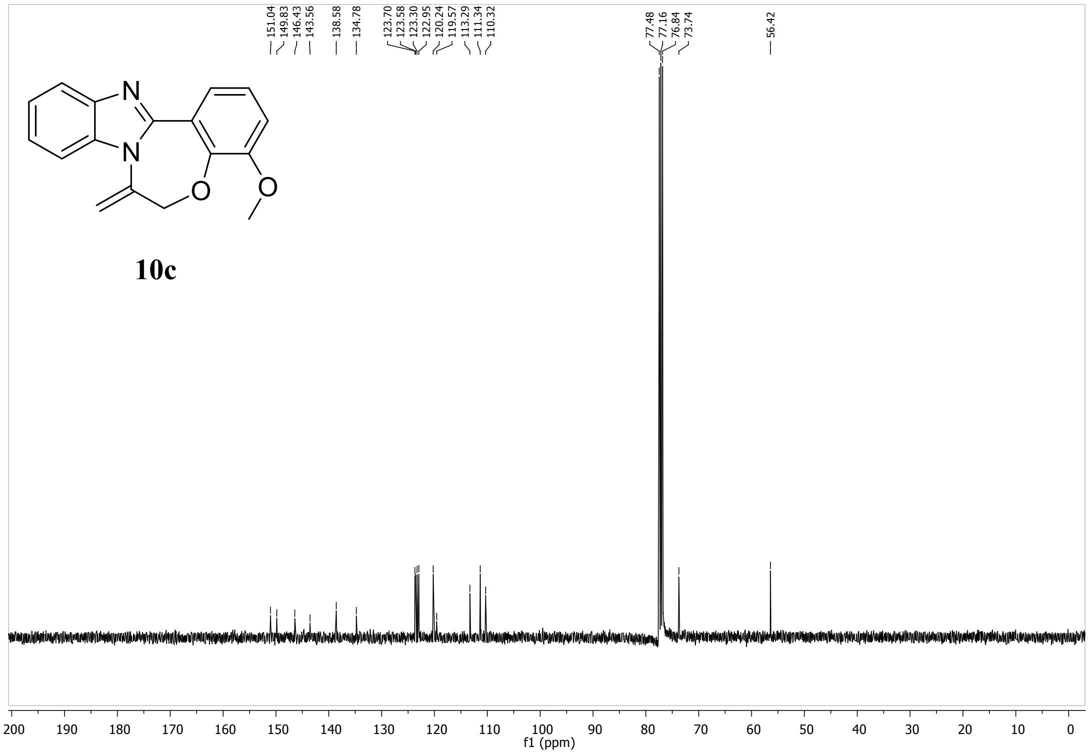

Figure S22. $^{13}C\{^{1}H\}$ NMR spectrum of **10c** in $CDCl_3$ solution (100 MHz).

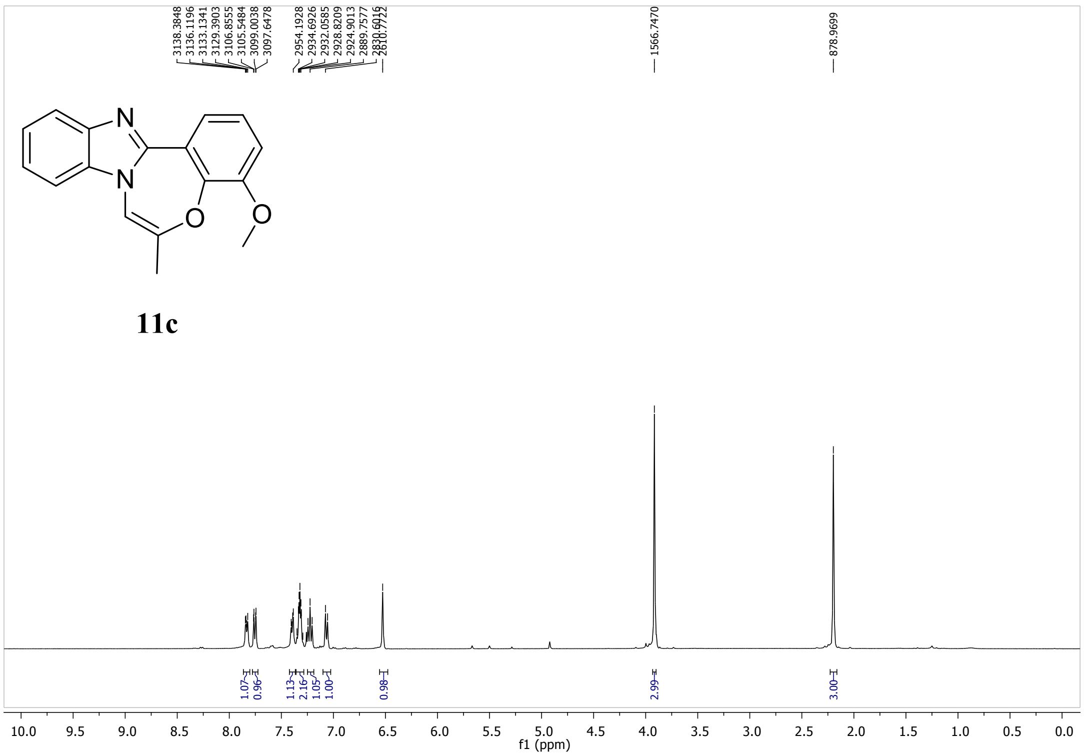

Figure S23. ^{1}H NMR spectrum of **11c** in $CDCl_3$ solution (400 MHz).

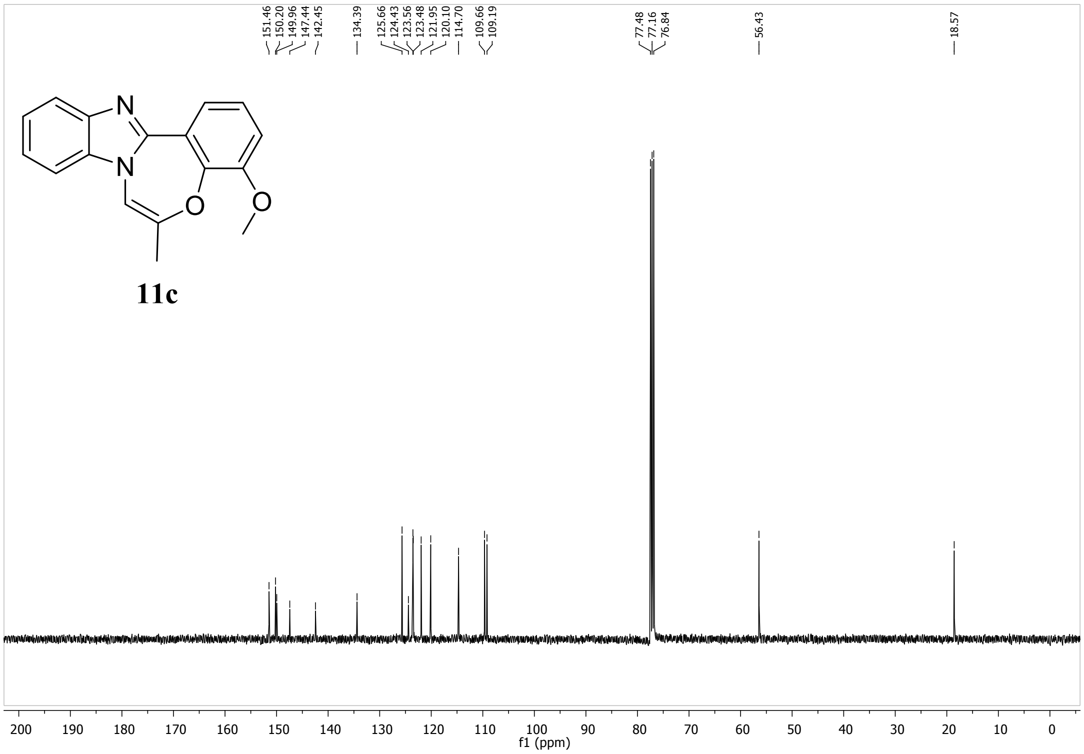

Figure S24. $^{13}C\{^{1}H\}$ NMR spectrum of **11c** in $CDCl_3$ solution (100 MHz).

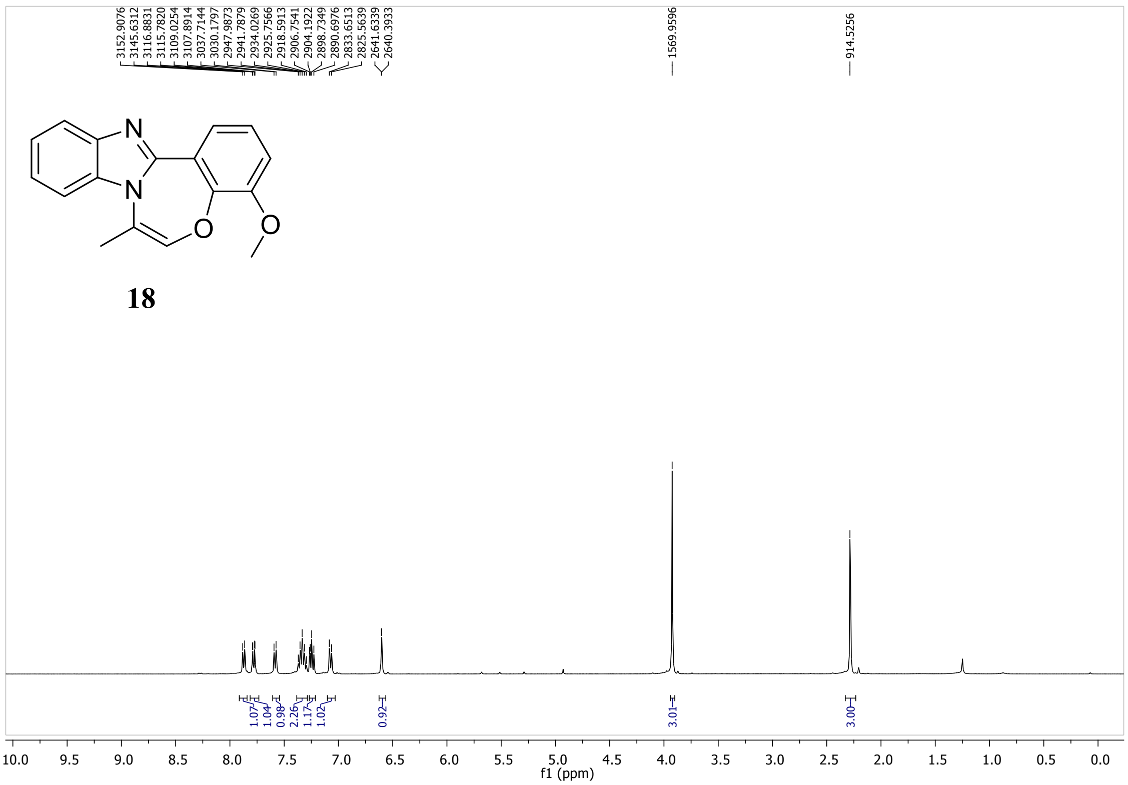

Figure S25. ^{1}H NMR spectrum of **18** in $CDCl_3$ solution (400 MHz).

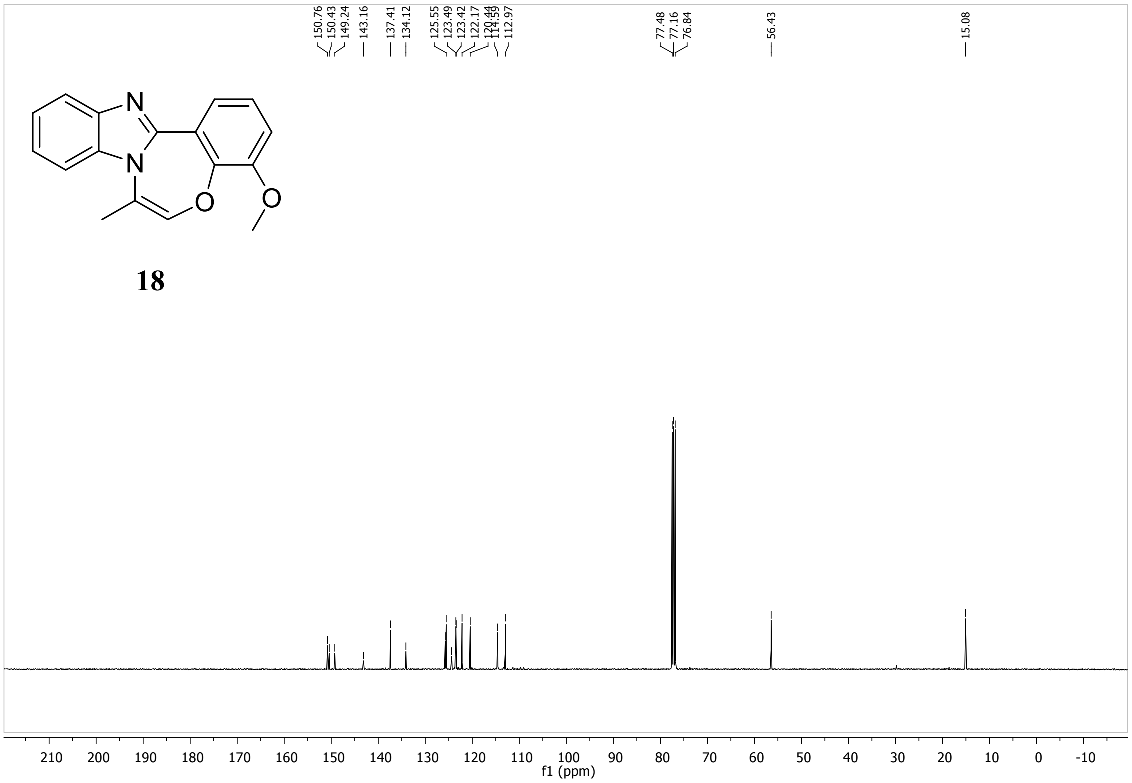

Figure S26. $^{13}C\{^1H\}$ NMR spectrum of **18** in $CDCl_3$ solution (100 MHz).

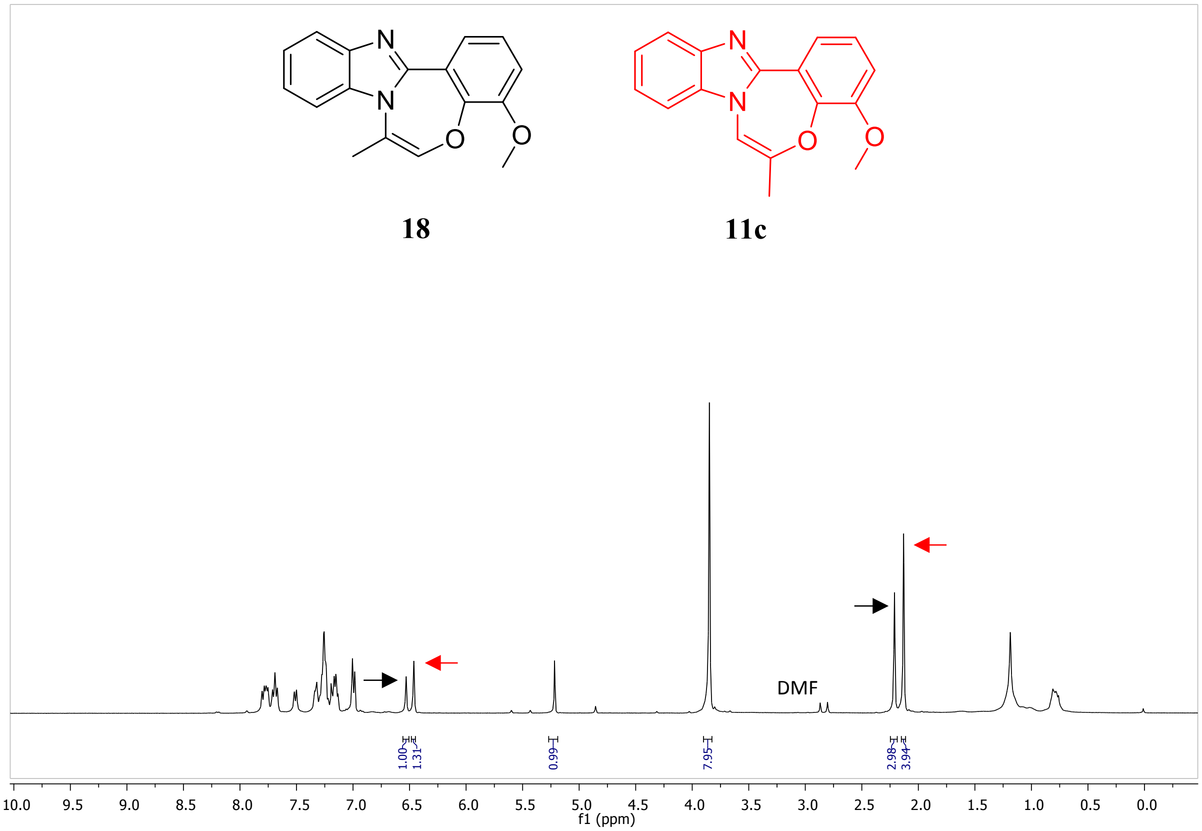

Figure S27. Crude ^{1}H NMR spectrum of mixture of **18** and **11c** from the heated reaction in $CDCl_3$ solution (400 MHz).

2. High-Resolution Mass Spectrometry (HR-MS) Data

Elemental Composition Report **Page 1**

Single Mass Analysis
Tolerance = 1000.0 PPM / DBE: min = -5.5, max = 1000.0
Element prediction: Off
Number of isotope peaks used for i-FIT = 9

Monoisotopic Mass, Even Electron Ions
2 formula(e) evaluated with 1 results within limits (all results (up to 1000) for each mass)
Elements Used:
C: 23-23 H: 18-19 N: 2-3 O: 1-3
Fevzi Can Inyurt
42870_20240314_05-04 8 (0.328) Cm (4:15) 1: TOF MS ES+ 9.55e+003

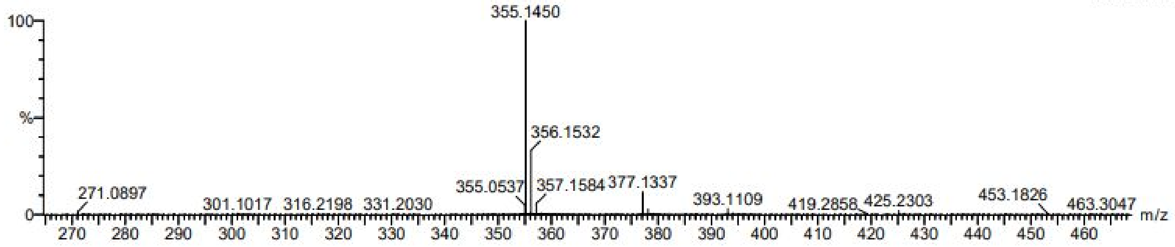

Minimum:				-5.5			
Maximum:		1000.0	1000.0	1000.0			
Mass	Calc. Mass	mDa	PPM	DBE	i-FIT	i-FIT (Norm)	Formula
355.1450	355.1447	0.3	0.8	15.5	636.3	0.0	C23 H19 N2 O2

Figure S28. HR-MS spectrum of **8b**

Elemental Composition Report **Page 1**

Single Mass Analysis
Tolerance = 10000.0 PPM / DBE: min = -5.5, max = 1000.0
Element prediction: Off
Number of isotope peaks used for i-FIT = 9

Monoisotopic Mass, Even Electron Ions
6 formula(e) evaluated with 1 results within limits (all results (up to 1000) for each mass)
Elements Used:
C: 22-22 H: 16-17 N: 1-2 O: 1-5
Fevzi Can Inyurt
41179_20230925_03-07 9 (0.362) Cm (9:15) 1: TOF MS ES+ 5.67e+006

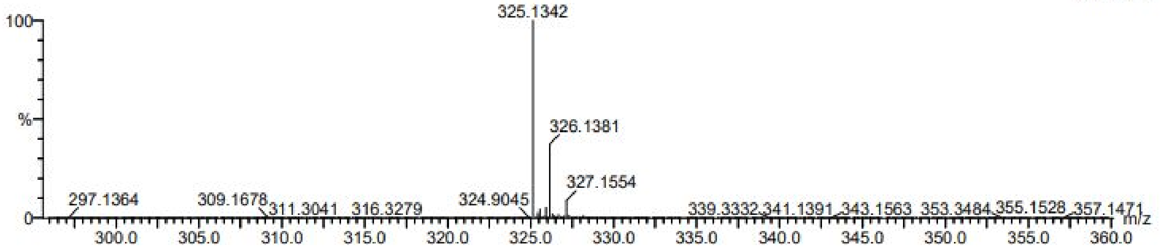

Minimum:				-5.5			
Maximum:		1000.0	10000.0	1000.0			
Mass	Calc. Mass	mDa	PPM	DBE	i-FIT	i-FIT (Norm)	Formula
325.1342	325.1341	0.1	0.3	15.5	1530.9	0.0	C22 H17 N2 O

Figure S29. HR-MS spectrum of *(Z)*-**9a**

Elemental Composition Report

Page 1

Single Mass Analysis

Tolerance = 10000.0 PPM / DBE: min = -5.5, max = 1000.0
Element prediction: Off
Number of isotope peaks used for i-FIT = 9

Monoisotopic Mass, Even Electron Ions
4 formula(e) evaluated with 1 results within limits (all results (up to 1000) for each mass)
Elements Used:
C: 23-23 H: 18-19 N: 1-2 O: 1-5
Fevzi Can Inyurt
41179_20230925_02-01 6 (0.260) Cm (5:11)

1: TOF MS ES+
4.60e+004

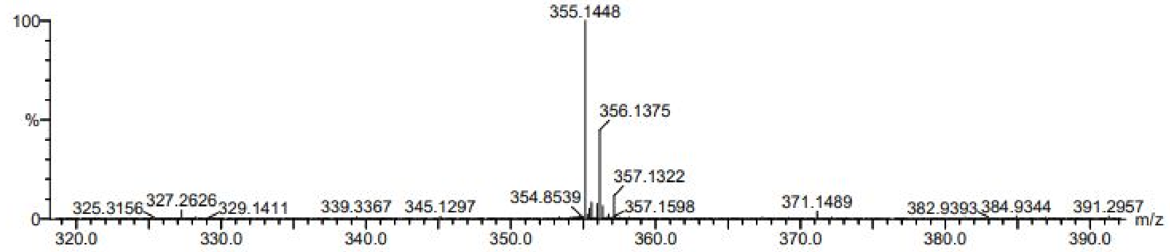

Minimum:				-5.5			
Maximum:		1000.0	10000.0	1000.0			
Mass	Calc. Mass	mDa	PPM	DBE	i-FIT	i-FIT (Norm)	Formula
355.1448	355.1447	0.1	0.3	15.5	1023.6	0.0	C23 H19 N2 O2

Figure S30. HR-MS spectrum of *(Z)*-**9b**

Elemental Composition Report

Page 1

Single Mass Analysis

Tolerance = 10000.0 PPM / DBE: min = -5.5, max = 1000.0
Element prediction: Off
Number of isotope peaks used for i-FIT = 9

Monoisotopic Mass, Odd and Even Electron Ions
4 formula(e) evaluated with 1 results within limits (all results (up to 1000) for each mass)
Elements Used:
C: 23-23 H: 18-19 N: 1-2 O: 1-5
Fevzi Can Inyurt
41179_20230925_01-03 9 (0.362) Cm (1:13)

1: TOF MS ES+
1.25e+005

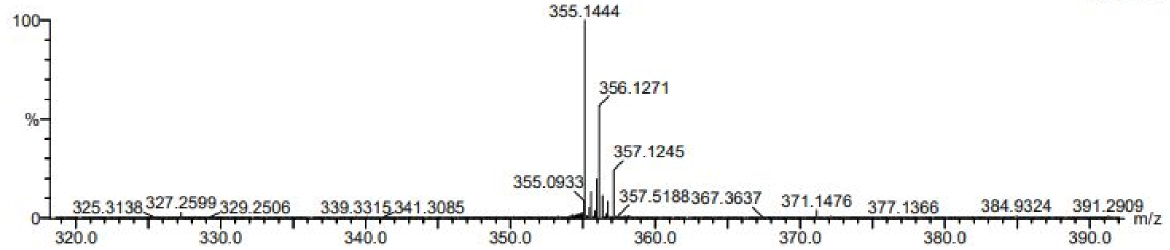

Minimum:				-5.5			
Maximum:		1000.0	10000.0	1000.0			
Mass	Calc. Mass	mDa	PPM	DBE	i-FIT	i-FIT (Norm)	Formula
355.1444	355.1447	-0.3	-0.8	15.5	1069.4	0.0	C23 H19 N2 O2

Figure S31. HR-MS spectrum of *(E)*-**9b**

Elemental Composition Report **Page 1**

Single Mass Analysis
Tolerance = 1000.0 PPM / DBE: min = -5.5, max = 1000.0
Element prediction: Off
Number of isotope peaks used for i-FIT = 9

Monoisotopic Mass, Odd and Even Electron Ions
1 formula(e) evaluated with 1 results within limits (all results (up to 1000) for each mass)
Elements Used:
C: 22-22 H: 15-16 N: 2-3 O: 1-3
Fevzi Can Inyurt
42870_20240314_04-05 13 (0.518) Cm (13:16) 1: TOF MS ES+ 2.02e+004

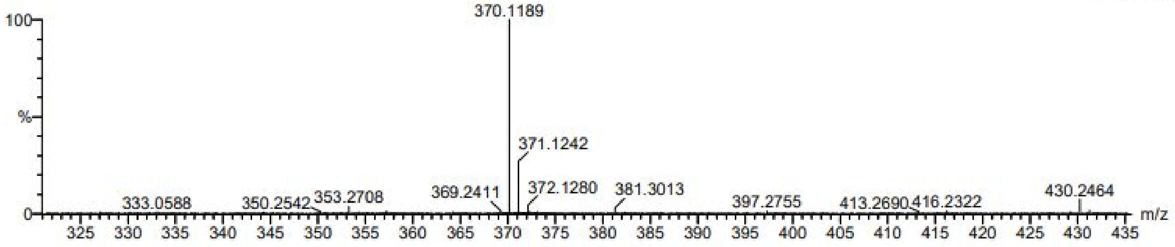

Minimum:				-5.5			
Maximum:		1000.0	1000.0	1000.0			
Mass	Calc. Mass	mDa	PPM	DBE	i-FIT	i-FIT (Norm)	Formula
370.1189	370.1192	-0.3	-0.8	16.5	618.6	0.0	C22 H16 N3 O3

Figure S32. HR-MS spectrum of *(E)*-**9c**

Elemental Composition Report **Page 1**

Single Mass Analysis
Tolerance = 1000.0 PPM / DBE: min = -5.5, max = 1000.0
Element prediction: Off
Number of isotope peaks used for i-FIT = 9

Monoisotopic Mass, Odd and Even Electron Ions
4 formula(e) evaluated with 1 results within limits (all results (up to 1000) for each mass)
Elements Used:
C: 16-16 H: 12-13 N: 2-3 O: 1-3
Fevzi Can Inyurt
42870_20240314_01-06 4 (0.172) Cm (1:13) 1: TOF MS ES+ 2.10e+004

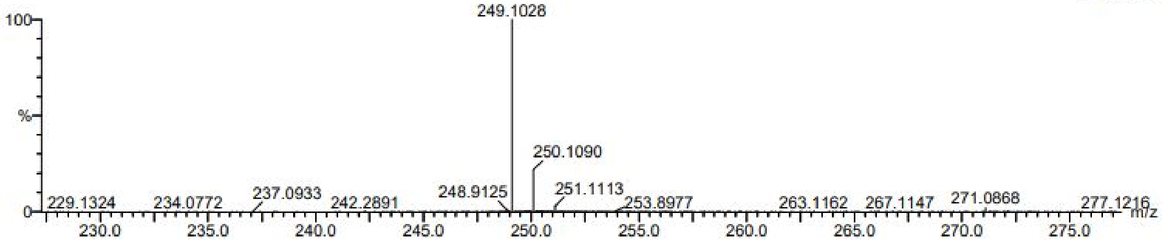

Minimum:				-5.5			
Maximum:		1000.0	1000.0	1000.0			
Mass	Calc. Mass	mDa	PPM	DBE	i-FIT	i-FIT (Norm)	Formula
249.1028	249.1028	0.0	0.0	11.5	744.6	0.0	C16 H13 N2 O

Figure S33. HR-MS spectrum of **11a**

Elemental Composition Report

Page 1

Single Mass Analysis

Tolerance = 1000.0 PPM / DBE: min = -5.5, max = 1000.0
Element prediction: Off
Number of isotope peaks used for i-FIT = 9

Monoisotopic Mass, Odd and Even Electron Ions
40 formula(e) evaluated with 1 results within limits (all results (up to 1000) for each mass)
Elements Used:
C: 16-16 H: 12-13 N: 2-3 O: 1-3 79Br: 0-2 81Br: 0-2

Fevzi Can Inyurt
42870_20240314_02-02 3 (0.138) Cm (2:14)

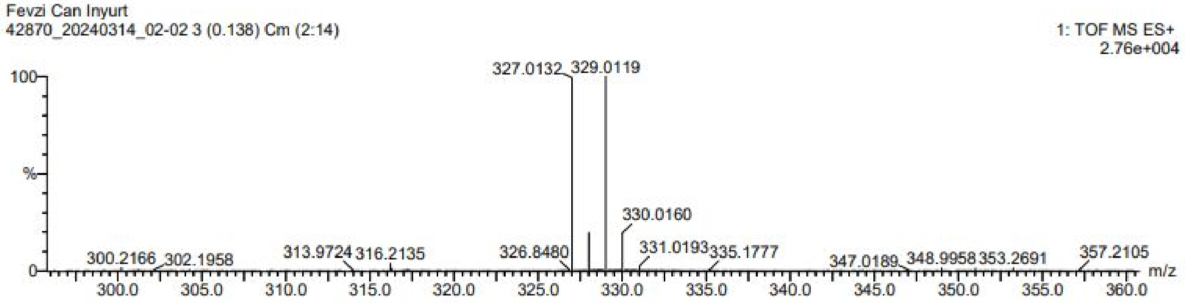

Minimum:				-5.5			
Maximum:		1000.0	1000.0	1000.0			
Mass	Calc. Mass	mDa	PPM	DBE	i-FIT	i-FIT (Norm)	Formula
327.0132	327.0133	-0.1	-0.3	11.5	1122.2	0.0	C16 H12 N2 O 79Br

Figure S34. HR-MS spectrum of **11b**

Elemental Composition Report

Page 1

Single Mass Analysis

Tolerance = 1000.0 PPM / DBE: min = -5.5, max = 1000.0
Element prediction: Off
Number of isotope peaks used for i-FIT = 9

Monoisotopic Mass, Even Electron Ions
2 formula(e) evaluated with 1 results within limits (all results (up to 1000) for each mass)
Elements Used:
C: 17-17 H: 12-15 N: 2-3 O: 1-3
Fevzi Can Inyurt
42870_20240314_03-04 8 (0.328) Cm (1:21)

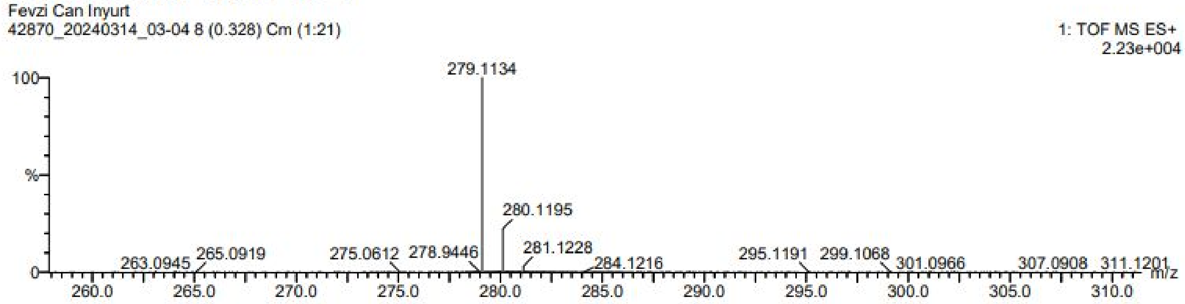

Minimum:				-5.5			
Maximum:		1000.0	1000.0	1000.0			
Mass	Calc. Mass	mDa	PPM	DBE	i-FIT	i-FIT (Norm)	Formula
279.1134	279.1134	0.0	0.0	11.5	795.3	0.0	C17 H15 N2 O2

Figure S35. HR-MS spectrum of **11c**

Elemental Composition Report **Page 1**

Single Mass Analysis
Tolerance = 1000.0 PPM / DBE: min = -5.5, max = 1000.0
Element prediction: Off
Number of isotope peaks used for i-FIT = 9

Monoisotopic Mass, Odd and Even Electron Ions
2 formula(e) evaluated with 1 results within limits (all results (up to 1000) for each mass)
Elements Used:
C: 17-17 H: 14-15 N: 2-3 O: 1-3
Fevzi Can Inyurt
42870_20240314_06-03 14 (0.552) Cm (12:16) 1: TOF MS ES+ 4.26e+004

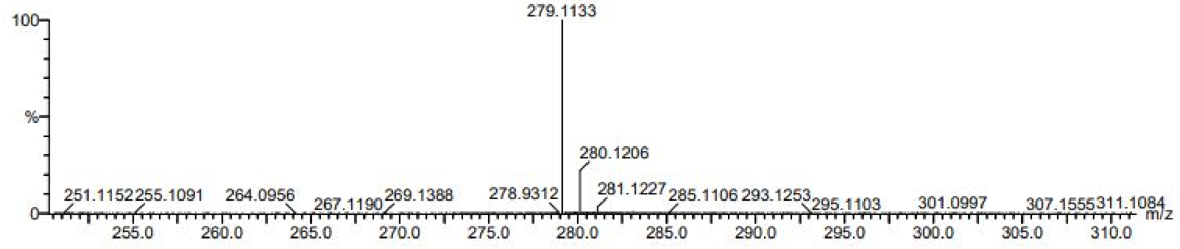

Minimum:				-5.5			
Maximum:		1000.0	1000.0	1000.0			
Mass	Calc. Mass	mDa	PPM	DBE	i-FIT	i-FIT (Norm)	Formula
279.1133	279.1134	-0.1	-0.4	11.5	908.1	0.0	C17 H15 N2 O2

Figure S36. HR-MS spectrum of **18**

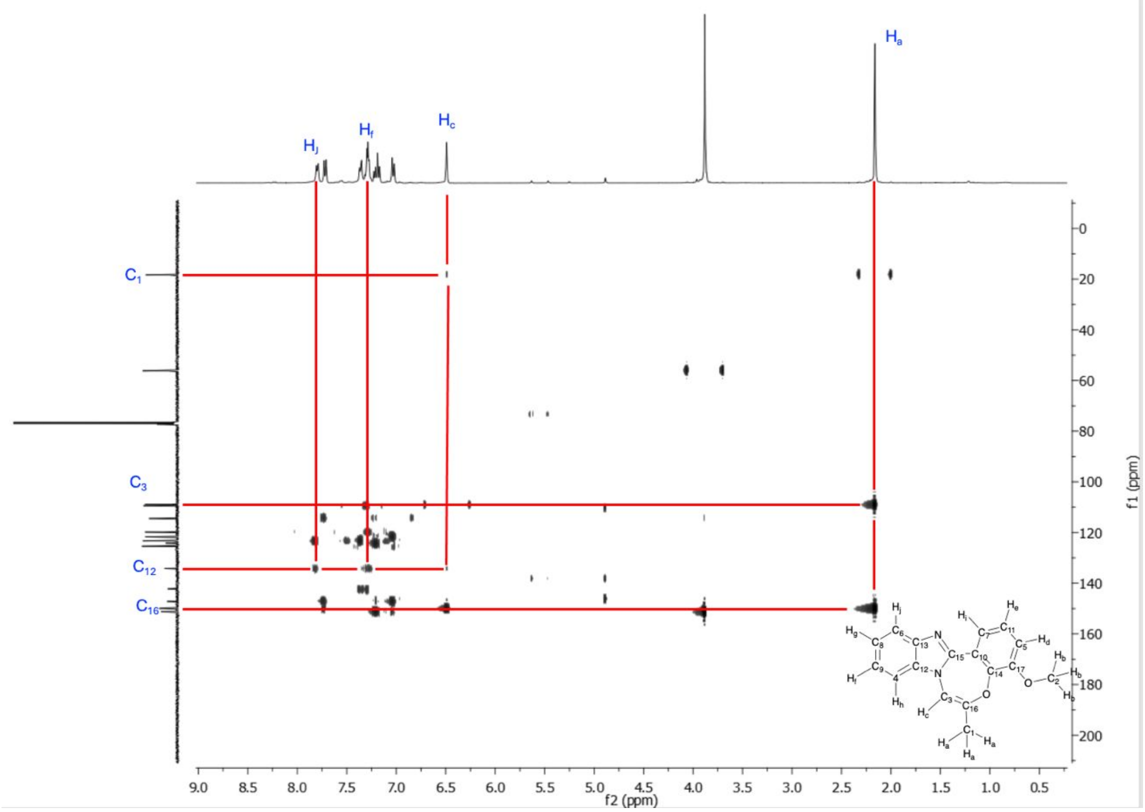

Figure S37. HMBC spectrum of **11c**.

3. Theoretical Calculations

Absolute Energies of the Structures

Energies are given in terms of ZPE-corrected total energy (Eel+ZPE), enthalpy (Eel+H) and Gibbs free-energy (Eel+G) as extracted from Gaussian output of each structure.

Table S1. Absolute energies of optimized structures in DMF (PCM/M06-2X/631+G(d,p)).

Compound No	Eel[a]+ZPE[b] (au)	Eel+H[c] (au)	Eel+G[d] (au)	Imaginary Frequency (*i*)
**5a + H$^-$**	-801.722754	-801.705826	-801.767219	-
**TS1**	-801.723922	-801.707314	-801.768348	-294.0
**I-1 + H$_2$**	-801.762696	-801.744804	-801.808587	-

[a]Eel = Total electronic energy

[b]ZPE = Zero-point energy correction

[c]H = Enthalpy correction

[d]G = Gibbs free energy correction

Table S2. Absolute energies of optimized structures in gas phase (M06-2X/631+G(d,p)).

Compound No	Eel[a]+ZPE[b] (au)	Eel+H[c] (au)	Eel+G[d] (au)	Imaginary Frequency (*i*)
**TS2p**	-800.497695	-800.483068	-800.538959	-364.7
**I-2**	-800.516686	-800.502352	-800.556873	-
**I-2 + H$_2$O**	-876.915605	-876.898126	-876.960628	-
**TS3**	-876.912489	-876.895614	-876.955947	-1003.8
**10a + OH$^-$**	-876.911535	-876.894188	-876.955384	-

Table S3. Single point energies in DMF (PCM/M06-2X/6311++G(d,p)).

Compound No	Eel[a]	Eel+G[b] (au)
**5a + H^-**	-802.139984	-801.940596
**TS1**	-802.139776	-801.941520
**I-1 + H_2**	-802.180566	-801.981396
**TS2p**	-800.973184	-800.785117
**I-2**	-800.999051	-800.807289
**I-2 + H_2O**	-877.444084	-877.233308
**TS3**	-877.443153	-877.234581
**10a + OH^-**	-877.455050	-877.242528

[a]Eel = Total electronic energy

[b]G = Gibbs free energy correction extracted from the corresponding gas phase frequency calculations and added to single point energy Eel.

Table S4. Absolute energies of optimized structures in gas phase (M06-2X/631+G(d,p)).

Compound No	Eel[a]+ZPE[b] (au)	Eel+H[c] (au)	Eel+G[d] (au)	Imaginary Frequency (*i*)
**TS5**	-1600.945822	-1600.914081	-1601.009293	-655.3
**I-5**	-1601.004524	-1600.972465	-1601.068331	-
**I-5'**	-1601.584714	-1601.552782	-1601.648215	-
**TS6**	-1600.960861	-1600.928975	-1601.024793	-632.8
**I-6p**	-1601.039845	-1601.007252	-1601.105852	-
**I-6a**	-800.540386	-800.524456	-800.583340	-
**TS7a**	-800.526472	-800.511630	-800.567293	-652.7
**I-7**	-800.545816	-800.531032	-800.586387	-
**I-7 + H_2O**	-876.937185	-876.919285	-876.98222	-
**TS8**	-876.918160	-876.901169	-876.961813	-1136.8
**11a + OH^-**	-876.931122	-876.913664	-876.974888	-

Table S5. Single point energies in DMF (PCM/M06-2X/6311++G(d,p)).

Compound No	Eel[a]	Eel+G[b] (au)
**TS5**	-1601.974864	-1601.580374
**I-5**	-1602.041069	-1601.645692
**I-5'**	-1601.648215	-1601.648215
**TS6**	-1601.990264	-1601.596423
**I-6p**	-1602.064798	-1601.671453
**I-6a**	-801.0216090	-800.8365300
**TS7a**	-801.0003265	-800.8132345
**I-7**	-801.0206918	-800.8313468
**I-7 + H_2O**	-877.4605245	-877.2507225
**TS8**	-877.4444360	-877.2374750
**11a + OH^-**	-877.4645407	-877.2535987

Table S6. Absolute energies of optimized structures in gas phase (M06-2X/631+G(d,p)).

Compound No	Eel[a]+ZPE[b] (au)	Eel+H[c] (au)	Eel+G[d] (au)	Imaginary Frequency (*i*)
**TS2a**	-800.491515	-800.476866	-800.531940	-354.8
**TS4**	-800.425637	-800.410371	-800.467891	-705.0
**TS7p**	-800.509759	-800.495276	-800.550196	-342.8

Table S7. Single point energies in DMF (PCM/M06-2X/6311++G(d,p)).

Compound No	Eel[a]	Eel+G[b] (au)
**TS2a**	-800.978123	-800.790187
**TS4**	-800.901978	-800.715908
**TS7p**	-796.246296	-796.057241

Table S8. Absolute energies of optimized structures in gas phase (M06-2X/631+G(d,p)).

Compound No	Eel[a]+ZPE[b] (au)	Eel+H[c] (au)	Eel+G[d] (au)	Imaginary Frequency (*i*)
**(*Z*)-9a**	-1032.003729	-1031.984711	-1032.051092	-
**(*E*)-9a**	-1032.002310	-1031.983354	-1032.049448	-
**(*Z*)-9b**	-1146.454417	-1146.432852	-1146.505032	-
**(*E*)-9b**	-1146.449850	-1146.428249	-1146.500747	-

Table S9. Single point energies in DMF (PCM/M06-2X/6311++G(d,p)).

Compound No	Eel[a]	Eel+G[b] (au)
**(*Z*)-9a**	-1032.557128	-1032.275797
**(*E*)-9a**	-1032.555229	-1032.273291
**(*Z*)-9b**	-1147.073026	-1146.762022
**(*E*)-9b**	-1147.067913	-1146.757177

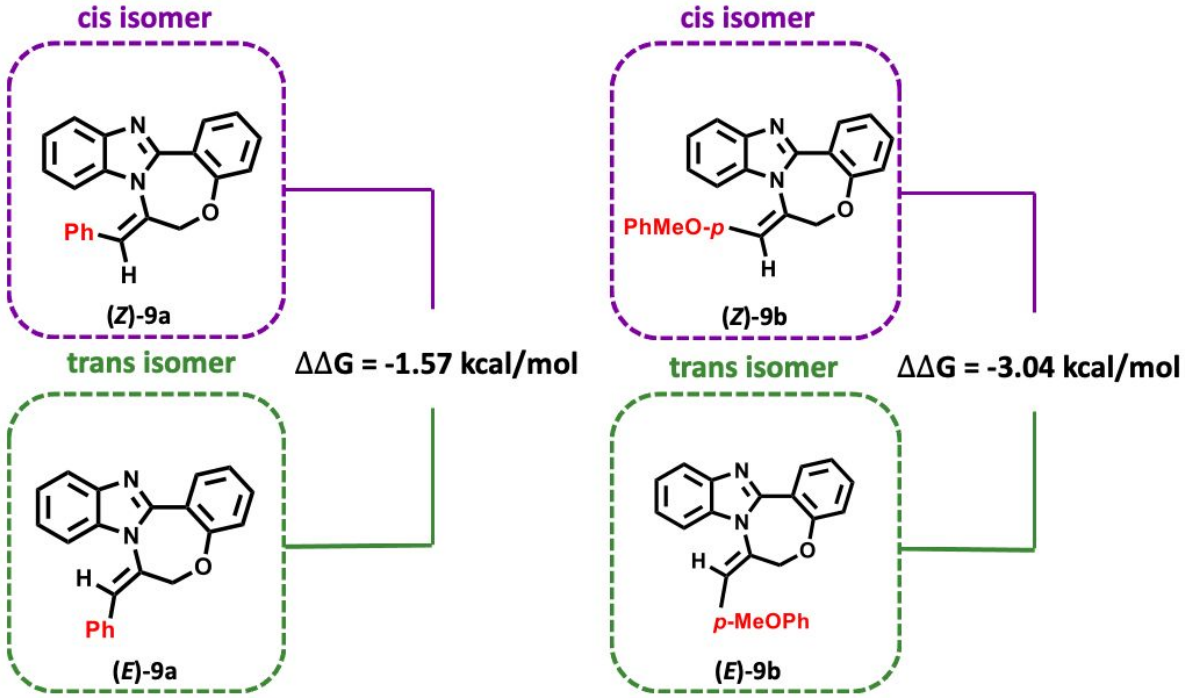

Figure S37. Energy differences of E/Z isomers of **9a** and **9b**.

5a + H

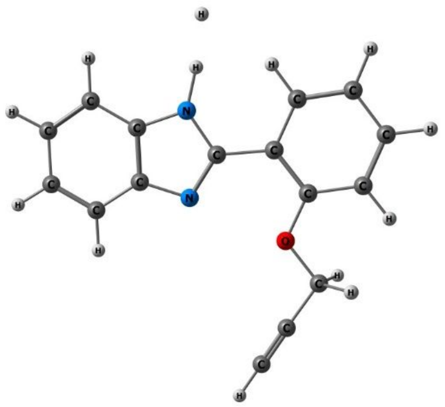

Atom type, (x,y,z) coordinates

C	-2.600180	-0.730963	-0.000258
C	-2.162711	0.612860	-0.000023
C	-3.096623	1.660151	0.000182
C	-4.444799	1.328809	0.000118
C	-4.869449	-0.018698	-0.000105
C	-3.960859	-1.068662	-0.000279
H	-2.767705	2.695345	0.000361
H	-5.191269	2.117382	0.000249
H	-5.933178	-0.236779	-0.000149
H	-4.289911	-2.102547	-0.000479
C	0.976928	-1.134340	-0.000100
C	2.125190	-0.306765	-0.000132
C	1.171443	-2.522550	0.000060
C	3.402075	-0.878550	0.000013
C	2.441384	-3.090887	0.000169
H	0.304977	-3.178686	0.000081
C	3.558540	-2.263065	0.000164
H	4.286539	-0.254387	-0.000001
H	2.551735	-4.170081	0.000282
H	4.559520	-2.682982	0.000264
C	-0.408678	-0.620560	-0.000186
N	-1.464365	-1.497063	-0.000622
N	-0.785164	0.652186	0.000018
O	1.931239	1.038524	-0.000287
C	3.080794	1.872184	0.000124
C	2.626668	3.264853	0.000290
C	2.276457	4.419176	0.000427
H	1.962226	5.441201	0.000449
H	3.693270	1.682652	0.890323
H	3.693681	1.683004	-0.889867
H	-1.526155	-2.614215	-0.000128
H	-1.744010	-3.911907	0.002352

TS1

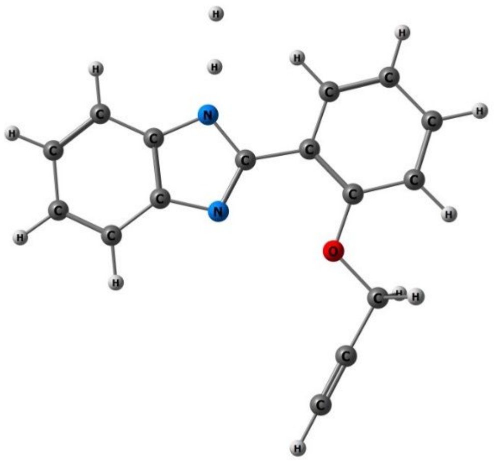

Atom type, (x,y,z) coordinates

C	-2.598913	-0.729996	0.002883
C	-2.162147	0.614665	-0.003122
C	-3.096249	1.662002	-0.006553
C	-4.444489	1.330903	-0.003993
C	-4.869114	-0.016723	0.002001
C	-3.960351	-1.066518	0.005610
H	-2.767444	2.697293	-0.011180
H	-5.190917	2.119571	-0.006771
H	-5.932880	-0.234817	0.003762
H	-4.290190	-2.100184	0.010120
C	0.975481	-1.134091	-0.001041
C	2.124553	-0.307784	-0.000638
C	1.169512	-2.522367	-0.001712
C	3.401134	-0.880590	-0.001104
C	2.438864	-3.092025	-0.002424
H	0.302119	-3.176831	-0.002294
C	3.556799	-2.265145	-0.001969
H	4.286028	-0.257029	-0.000722
H	2.548357	-4.171315	-0.003189
H	4.557440	-2.685869	-0.002382
C	-0.410745	-0.620847	-0.000348
N	-1.464750	-1.498383	0.004743
N	-0.785086	0.653647	-0.004990
O	1.932250	1.037949	0.000370
C	3.082748	1.870089	0.001946
C	2.630488	3.263399	0.003186
C	2.281660	4.418143	0.004118
H	1.968910	5.440618	0.004933
H	3.694788	1.679054	0.892144
H	3.695800	1.681066	-0.887987
H	-1.538638	-2.636799	0.008558
H	-1.757913	-3.883884	0.012726

I-1 + H_2

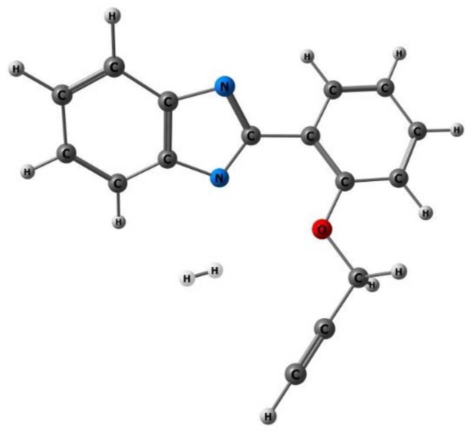

Atom type, (x,y,z) coordinates

C	-2.474547	-0.657357	-0.473299	H	4.286252	-0.146173	0.069331
C	-2.182857	0.413795	0.422666	H	2.672905	-4.116871	0.117981
C	-3.206936	1.266094	0.873254	H	4.629318	-2.568476	0.146063
C	-4.500097	1.035723	0.420729	C	-0.397322	-0.650831	-0.016016
C	-4.788178	-0.025156	-0.470043	N	-1.313383	-1.332227	-0.739409
C	-3.788251	-0.876026	-0.923895	N	-0.840846	0.400633	0.700961
H	-2.990162	2.082219	1.558540	O	1.873071	1.067137	-0.056326
H	-5.308928	1.679880	0.754620	C	2.989439	1.939108	-0.128830
H	-5.812085	-0.174414	-0.801692	C	2.486917	3.310640	-0.244679
H	-4.013476	-1.692320	-1.606252	C	2.091287	4.446197	-0.340137
C	1.005918	-1.130335	0.025479	H	1.736178	5.451069	-0.424331
C	2.125872	-0.271784	0.024721	H	3.608937	1.850011	0.772500
C	1.239051	-2.508566	0.050794	H	3.611303	1.697922	-1.000215
C	3.420620	-0.797084	0.071036	H	-1.686016	-2.213984	1.737015
C	2.526755	-3.042008	0.092783	H	-1.998433	-2.342885	2.397252
H	0.372931	-3.162975	0.039892				
C	3.616713	-2.178900	0.108612				

TS2p

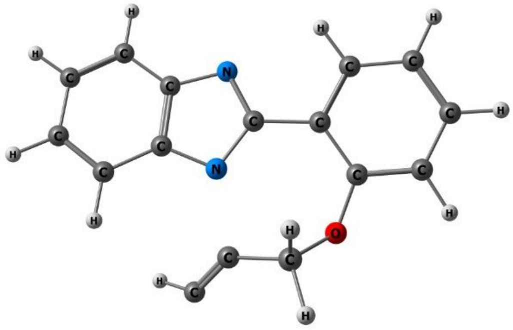

Atom type, (x,y,z) coordinates

C	1.921597	-1.288003	-0.071894
C	1.956555	0.131585	0.027341
C	3.180069	0.813478	0.141925
C	4.345308	0.059315	0.120224
C	4.315079	-1.348191	-0.005921
C	3.112422	-2.032327	-0.095589
H	3.210801	1.889463	0.268037
H	5.305126	0.561554	0.210868
H	5.251799	-1.899727	-0.019584
H	3.076748	-3.115747	-0.172462
N	0.622835	-1.708303	-0.105738
N	0.659138	0.566313	0.037272
C	-1.560427	-0.626678	-0.007512
C	-2.143223	-1.903938	0.093332
C	-2.435948	0.473135	-0.104725
C	-3.516568	-2.101300	0.075265
H	-1.460167	-2.743308	0.166064
C	-3.822410	0.270707	-0.127819
C	-4.368615	-1.001862	-0.044694
H	-3.919835	-3.107025	0.149451
H	-4.447695	1.153729	-0.216104
H	-5.447010	-1.133330	-0.065514
C	-0.080134	-0.570057	-0.034072
O	-2.053188	1.771312	-0.253437
C	-1.083588	2.330874	0.645745
C	0.241719	2.454767	0.008393
C	1.007492	3.327754	-0.495785
H	1.957364	3.227933	-0.997610
H	-1.447566	3.333714	0.883081
H	-1.043844	1.720622	1.555241

I-2

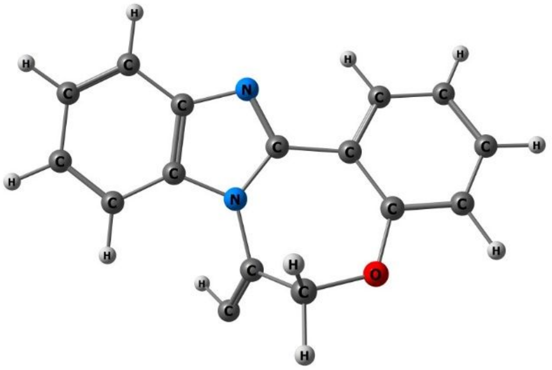

Atom type, (x,y,z) coordinates

C	-1.946771	-1.202793	-0.111819
C	-1.986252	0.198184	0.074086
C	-3.186983	0.912236	0.137611
C	-4.359108	0.176935	0.023926
C	-4.339440	-1.225421	-0.144919
C	-3.144254	-1.926026	-0.217983
H	-3.184228	1.989485	0.266770
H	-5.314973	0.691433	0.068167
H	-5.281595	-1.761271	-0.223674
H	-3.119965	-3.002496	-0.359892
N	-0.645571	-1.631682	-0.181091
N	-0.671834	0.600431	0.128923
C	1.558718	-0.567694	-0.007077
C	2.109954	-1.843419	0.237190
C	2.456870	0.511002	-0.176376
C	3.470817	-2.068100	0.341938
H	1.403913	-2.658784	0.350159
C	3.841507	0.271800	-0.057266
C	4.347801	-0.987172	0.200336
H	3.845455	-3.067295	0.540791
H	4.493250	1.130221	-0.184149
H	5.421735	-1.129210	0.287173
C	0.080195	-0.528302	-0.041106
O	2.177022	1.795504	-0.457982
C	0.845141	2.223115	-0.824391
C	-0.196238	1.984100	0.220573
C	-0.653908	2.931425	1.060063
H	-1.400891	2.472995	1.738922
H	0.953265	3.297376	-0.963295
H	0.601407	1.733042	-1.780665

I-2 + H_2O

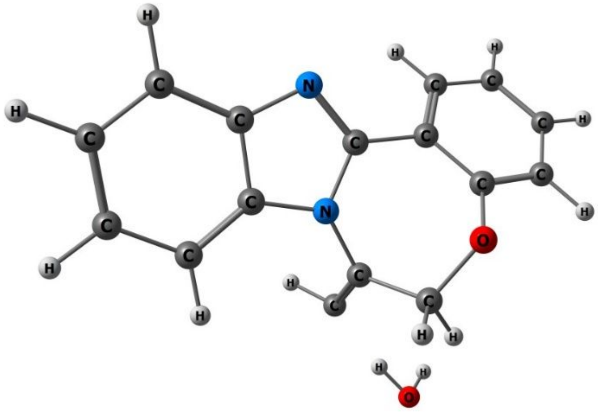

Atom type, (x,y,z) coordinates

C	-3.549592	-0.598352	0.589625
C	-2.173575	-0.380032	0.680836
C	-1.290683	-1.200809	-0.044855
C	-1.806645	-2.238708	-0.824702
C	-3.178293	-2.457887	-0.906377
C	-4.050805	-1.628515	-0.200861
H	-4.204237	0.046633	1.166983
H	-1.102619	-2.868569	-1.360158
H	-3.564212	-3.265883	-1.520405
H	-5.124110	-1.786162	-0.262010
C	0.161159	-0.986664	0.015684
C	2.273845	-1.251983	-0.016376
N	0.670979	0.281381	0.085931
C	3.071245	1.080260	0.080411
C	3.591360	-1.729989	-0.046585
C	4.367030	0.583658	0.048307
H	2.852114	2.143102	0.110619
C	4.624379	-0.803624	-0.012769
H	3.783098	-2.797610	-0.098654
H	5.203451	1.276499	0.064401
H	5.654373	-1.148513	-0.038649
O	-1.726764	0.524904	1.592673
C	-1.013336	1.712851	1.155452
C	-0.111792	1.514984	-0.028360
C	-0.004393	2.402822	-1.026968
H	0.765126	2.087197	-1.752540
H	-1.730122	2.502901	0.911330
O	-1.826338	4.522893	-0.866232
H	-1.094305	3.806768	-0.957939
H	-2.483479	4.254011	-1.515679
C	2.037496	0.141130	0.057391
N	1.077210	-1.935933	-0.047632
H	-0.451995	1.994257	2.053939

TS3

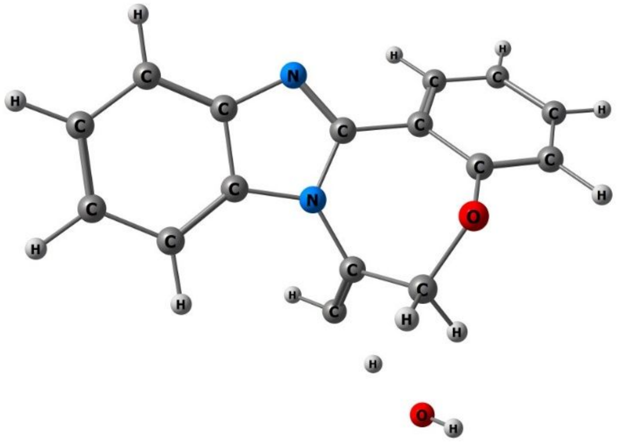

Atom type, (x,y,z) coordinates

C	-3.578920	-0.486667	0.565826
C	-2.198867	-0.301701	0.659092
C	-1.332429	-1.164217	-0.035665
C	-1.866435	-2.213786	-0.787507
C	-3.242415	-2.400796	-0.870440
C	-4.098614	-1.528369	-0.196832
H	-4.220931	0.194526	1.114752
H	-1.175161	-2.877009	-1.298770
H	-3.645410	-3.217453	-1.461596
H	-5.174891	-1.661374	-0.262513
C	0.122865	-0.978551	0.030178
C	2.231524	-1.287707	0.020704
N	0.663570	0.281734	0.065529
C	3.084714	1.025415	0.057817
C	3.536309	-1.798831	0.014882
C	4.368080	0.496046	0.051053
H	2.894713	2.094104	0.061704
C	4.591701	-0.897618	0.030398
H	3.701249	-2.871842	-0.003915
H	5.220690	1.168781	0.056961
H	5.612941	-1.267915	0.023090
O	-1.721962	0.620812	1.537994
C	-1.044065	1.798341	1.034334
C	-0.083450	1.515256	-0.087689
C	0.083480	2.368173	-1.101137
H	0.845768	2.095264	-1.839356
H	-1.759498	2.552614	0.685726
O	-1.583689	4.332814	-0.727773
H	-0.689235	3.403701	-1.064655
H	-2.037368	4.689049	-1.499438
C	2.028790	0.111361	0.050378
N	1.019116	-1.945001	0.002194
H	-0.520068	2.173315	1.919825

10a + OH^-

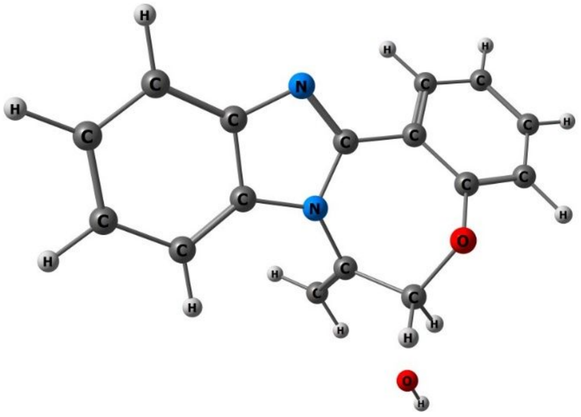

Atom type, (x,y,z) coordinates

C	-3.552130	-0.515445	0.550759
C	-2.174384	-0.316817	0.641516
C	-1.297831	-1.192546	-0.021979
C	-1.815707	-2.275411	-0.736831
C	-3.189660	-2.478031	-0.814834
C	-4.056790	-1.589376	-0.176074
H	-4.202258	0.183009	1.067158
H	-1.116934	-2.949510	-1.223051
H	-3.583646	-3.319448	-1.376581
H	-5.131294	-1.734870	-0.243001
C	0.153210	-0.975400	0.041560
C	2.270372	-1.236824	0.054677
N	0.670341	0.298865	0.029479
C	3.078881	1.091166	0.017415
C	3.584074	-1.722735	0.078709
C	4.372263	0.586699	0.040705
H	2.871715	2.156142	-0.011974
C	4.622292	-0.801661	0.070791
H	3.768532	-2.792339	0.102014
H	5.211299	1.276055	0.033457
H	5.650165	-1.152452	0.086439
O	-1.693536	0.641652	1.477622
C	-1.103516	1.829563	0.895908
C	-0.094884	1.502038	-0.171821
C	0.112395	2.290456	-1.225382
H	0.882777	2.032059	-1.950409
H	-1.847429	2.528001	0.476329
O	-1.903924	4.181894	-0.574899
H	-0.524475	3.193691	-1.307987
H	-2.472398	4.904620	-0.859712
C	2.041019	0.157285	0.029275
N	1.070798	-1.918948	0.055014
H	-0.611964	2.309486	1.747719

TS5

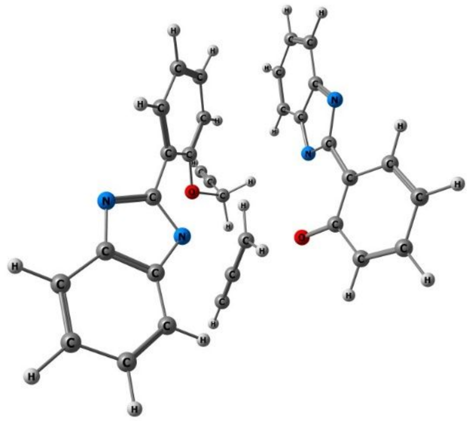

Atom type, (x,y,z) coordinates

C	-4.373132	-1.313909	-1.044356
C	-3.937276	-0.266564	-0.182261
C	-4.845051	0.651702	0.365216
C	-6.188103	0.505973	0.037353
C	-6.629091	-0.530774	-0.813659
C	-5.733801	-1.444554	-1.358428
H	-4.507230	1.441446	1.030457
H	-6.915772	1.203155	0.446010
H	-7.688815	-0.612065	-1.045320
H	-6.068014	-2.243700	-2.015589
N	-3.290816	-2.055564	-1.441013
N	-2.581865	-0.385653	-0.063361
C	-0.863265	-1.889242	-1.061570
C	-0.468942	-2.147559	-2.378241
C	0.101298	-2.005133	-0.043375
C	0.852336	-2.447829	-2.697528
H	-1.228479	-2.067331	-3.150101
C	1.426994	-2.306910	-0.358089
C	1.805347	-2.502809	-1.682606
H	1.141714	-2.598079	-3.733554
H	2.178327	-2.366462	0.420039
H	2.855832	-2.674678	-1.902209
C	-2.262532	-1.461583	-0.824513
O	-0.324259	-1.828377	1.244205
C	0.628011	-1.367070	2.200038
C	1.370374	-2.473283	2.827952
C	1.981398	-3.367757	3.364073
H	2.537095	-4.160133	3.809950
H	1.312099	-0.619095	1.761796
H	0.039972	-0.851111	2.963644
C	3.913195	0.450364	-0.968357
C	3.606876	0.091743	0.384691
C	4.285617	-0.963799	1.014231
C	5.245443	-1.663626	0.286848
C	5.544971	-1.314666	-1.050271
C	4.890261	-0.261403	-1.684047
H	4.045285	-1.238995	2.040035
H	5.774197	-2.493237	0.752270
H	6.301501	-1.881670	-1.590114
H	5.116175	0.003206	-2.715218
C	1.417160	2.849872	-0.198910
C	0.307079	2.963963	0.697055
C	1.642585	3.883484	-1.120535
C	-0.498619	4.130354	0.595260
C	0.838890	5.019281	-1.196443
H	2.484958	3.759409	-1.794793
C	-0.243979	5.140377	-0.322105
H	-1.336253	4.197088	1.286766
H	1.053851	5.794988	-1.927522
H	-0.889514	6.016886	-0.358928
C	2.371289	1.710283	-0.219727
N	3.119077	1.493297	-1.330184
N	2.608643	0.901702	0.839226
O	-0.034927	2.043947	1.568148
C	-1.365076	0.884511	0.817899
C	-1.922390	0.626405	2.129869
C	-2.423251	0.438535	3.212247
H	-2.857218	0.258623	4.168059
H	-1.776474	1.696352	0.228992
H	-0.532707	0.324098	0.427560

I-5

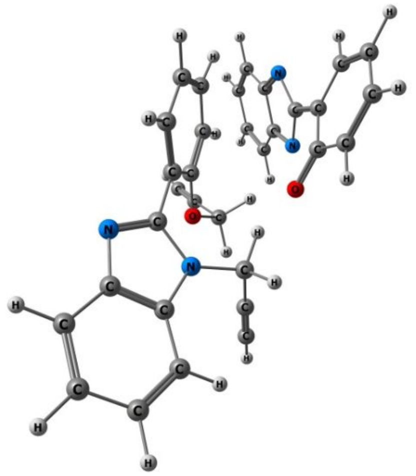

Atom type, (x,y,z) coordinates

C	4.959642	-0.319003	1.166607
C	4.605341	-0.348428	-0.200654
C	5.545475	-0.525760	-1.216723
C	6.872882	-0.678298	-0.825894
C	7.244202	-0.658044	0.532405
C	6.298866	-0.480248	1.537435
H	5.248847	-0.557908	-2.260664
H	7.637111	-0.821453	-1.584759
H	8.290815	-0.784794	0.796557
H	6.577965	-0.460406	2.586861
N	3.839752	-0.113766	1.940427
N	3.245376	-0.150451	-0.227084
C	1.451831	0.278905	1.478769
C	1.232440	1.328816	2.378878
C	0.353283	-0.441070	0.978325
C	-0.056284	1.698150	2.739447
H	2.098258	1.864963	2.756944
C	-0.941381	-0.089541	1.365683
C	-1.141081	0.994037	2.211455
H	-0.218753	2.550448	3.392062
H	-1.802199	-0.613206	0.964466
H	-2.166753	1.294047	2.411161
C	2.837288	-0.019507	1.085570
O	0.637635	-1.475089	0.142709
C	-0.433112	-1.986847	-0.658492
C	-1.272671	-2.943814	0.076557
C	-2.014581	-3.704897	0.650393
H	-2.745866	-4.294027	1.155540
H	-1.039264	-1.157764	-1.059111
H	0.064413	-2.489166	-1.491666
C	-4.698654	-0.224173	0.432190
C	-4.154580	-0.961171	-0.672144
C	-4.642835	-2.239771	-0.980426
C	-5.651561	-2.779787	-0.186339
C	-6.179169	-2.060452	0.909960
C	-5.712435	-0.786368	1.226094
H	-4.219240	-2.798013	-1.813057
H	-6.039355	-3.773320	-0.406909
H	-6.964733	-2.512167	1.514106
H	-6.120085	-0.231112	2.069194
C	-2.277243	2.073833	-0.782217
C	-0.932799	1.929517	-1.304477
C	-2.755284	3.342618	-0.442974
C	-0.205386	3.158035	-1.476665
C	-2.011931	4.512721	-0.613278
H	-3.757224	3.389559	-0.022028
C	-0.720757	4.400168	-1.143652
H	0.803536	3.066019	-1.876995
H	-2.427290	5.480137	-0.341451
H	-0.110457	5.292350	-1.291999
C	-3.174306	0.921992	-0.523513
N	-4.060090	0.976139	0.508446
N	-3.178126	-0.211893	-1.261246
O	-0.377961	0.808287	-1.573346
C	2.475040	0.060898	-1.447692
C	2.461822	-1.143969	-2.285991
C	2.480178	-2.120882	-2.996027
H	2.449936	-2.994860	-3.605278
H	2.947681	0.878065	-2.007916
H	1.433458	0.380883	-1.239416

I-5’

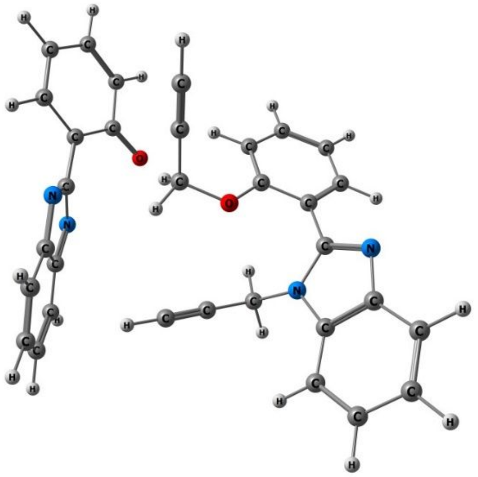

Atom type, (x,y,z) coordinates

C	-4.124260	1.701666	-0.329188
C	-2.786039	1.998466	-0.665427
C	-2.405579	3.210706	-1.245191
C	-3.418065	4.133033	-1.484613
C	-4.760606	3.856473	-1.153331
C	-5.127473	2.647341	-0.574874
H	-1.366658	3.422044	-1.478597
H	-3.167551	5.091207	-1.931131
H	-5.520222	4.607977	-1.352869
H	-6.159408	2.426240	-0.317541
N	-4.202171	0.440071	0.215395
N	-2.062231	0.883004	-0.318107
C	-2.622635	-1.380326	0.657050
C	-3.467844	-2.420221	0.256473
C	-1.513796	-1.670481	1.468890
C	-3.205618	-3.736004	0.615352
H	-4.327007	-2.165374	-0.356335
C	-1.239881	-2.996418	1.820339
C	-2.077337	-4.014748	1.389185
H	-3.857324	-4.536413	0.277829
H	-0.356076	-3.225783	2.402331
H	-1.837338	-5.041706	1.652134
C	-2.959993	-0.014329	0.211963
O	-0.792290	-0.615053	1.909703
C	0.573139	-0.829651	2.286862
C	0.688138	-1.299277	3.677159
C	0.801777	-1.682916	4.818194
H	0.901647	-2.023419	5.822739
H	1.064005	-1.491039	1.557759
H	1.055390	0.146169	2.184180
C	3.633777	1.632230	-0.384326
C	3.481333	0.770032	0.756817
C	3.813874	1.226874	2.041345
C	4.280937	2.534000	2.186178
C	4.431114	3.381509	1.065847
C	4.117172	2.939069	-0.219905
H	3.684171	0.576989	2.905646
H	4.533431	2.909920	3.176130
H	4.798196	4.396128	1.212315
H	4.214947	3.598802	-1.080176
C	2.247765	-1.267951	-1.890944
C	1.181815	-2.164997	-1.499079
C	2.798743	-1.358778	-3.172438
C	0.808577	-3.146480	-2.480782
C	2.400818	-2.319226	-4.105391
H	3.571879	-0.635946	-3.424946
C	1.394913	-3.221277	-3.733801
H	0.008878	-3.826321	-2.192324
H	2.861681	-2.364211	-5.089264
H	1.060210	-3.984962	-4.437711
C	2.778297	-0.220176	-0.986149
N	3.186929	0.975203	-1.487055
N	2.938457	-0.407176	0.345099
O	0.552590	-2.095219	-0.386188
C	-0.631406	0.726993	-0.607182
C	0.178577	1.676379	0.162089
C	0.884495	2.456063	0.754996
H	1.587543	3.079197	1.270027
H	-0.479443	0.908933	-1.678363
H	-0.308617	-0.309438	-0.413979

TS6

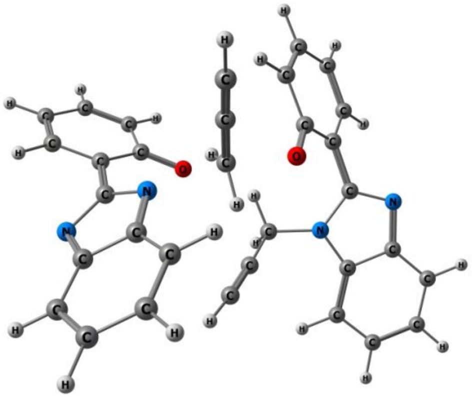

Atom type, (x,y,z) coordinates

C	4.999359	-0.777565	-0.239653
C	3.829513	-1.221614	-0.896170
C	3.815999	-2.339480	-1.732325
C	5.020589	-3.018104	-1.893614
C	6.194614	-2.596119	-1.240199
C	6.197528	-1.479353	-0.410575
H	2.901721	-2.671431	-2.214242
H	5.051915	-3.897590	-2.530757
H	7.114009	-3.156834	-1.388185
H	7.099297	-1.146512	0.095375
N	4.727095	0.352597	0.498278
N	2.848719	-0.330234	-0.532159
C	2.734941	1.767953	0.841225
C	3.345218	3.019570	0.713050
C	1.473173	1.635273	1.492889
C	2.711294	4.177696	1.147526
H	4.325885	3.063492	0.245422
C	0.828981	2.836024	1.891440
C	1.435840	4.068874	1.717637
H	3.184312	5.147200	1.022066
H	-0.152166	2.758143	2.348422
H	0.907366	4.966341	2.032898
C	3.441877	0.593777	0.305653
O	0.974757	0.451795	1.717201
C	-0.830194	0.142663	1.340732
C	-1.389812	0.462171	2.637977
C	-1.835496	0.722167	3.730411
H	-2.247774	0.951247	4.685131
H	-0.842856	0.852791	0.513846
H	-0.524497	-0.873617	1.152748
C	-3.960406	-2.189073	-0.127640
C	-2.964715	-1.807935	0.822702
C	-2.515737	-2.706380	1.801700
C	-3.061454	-3.988691	1.811981
C	-4.043389	-4.372919	0.875091
C	-4.501536	-3.482555	-0.094165
H	-1.766904	-2.411334	2.533407
H	-2.725925	-4.706108	2.557723
H	-4.447669	-5.382589	0.913535
H	-5.256887	-3.777120	-0.819570
C	-3.340245	1.154281	-1.182838
C	-2.115702	1.858885	-1.492868
C	-4.574980	1.696708	-1.550021
C	-2.288175	3.145248	-2.111897
C	-4.700936	2.941047	-2.168438
H	-5.459913	1.102726	-1.331546
C	-3.529260	3.663301	-2.437506
H	-1.375060	3.694416	-2.330422
H	-5.679460	3.337685	-2.426545
H	-3.592849	4.643785	-2.911484
C	-3.356760	-0.179791	-0.540796
N	-4.198608	-1.140478	-0.969347
N	-2.607830	-0.521296	0.544458
O	-0.946542	1.393182	-1.269854
C	1.524345	-0.272521	-1.146359
C	0.733695	-1.487439	-0.916784
C	0.038173	-2.462827	-0.768215
H	-0.642031	-3.266565	-0.581157
H	1.651200	-0.129788	-2.226814
H	0.968906	0.598088	-0.786941

I-6p

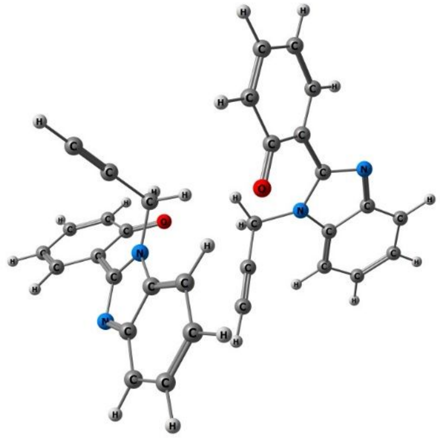

Atom type, (x,y,z) coordinates

C	4.806416	-1.184675	-0.069133
C	3.495095	-1.711656	-0.056286
C	3.232588	-3.077210	0.055365
C	4.334555	-3.922784	0.160260
C	5.648770	-3.417255	0.157066
C	5.898720	-2.052682	0.043938
H	2.214961	-3.455098	0.073246
H	4.175194	-4.993788	0.251330
H	6.482280	-4.109897	0.245245
H	6.910313	-1.656496	0.038137
N	4.764404	0.182064	-0.201532
N	2.666934	-0.624031	-0.191881
C	2.990497	1.862122	-0.490542
C	3.737545	2.666198	-1.361882
C	1.852776	2.399975	0.225923
C	3.390822	3.983138	-1.635826
H	4.609820	2.217820	-1.832286
H	1.511965	3.759565	-0.114942
H	2.251298	4.513135	-1.003152
H	3.974060	4.578554	-2.332071
H	0.635909	4.173724	0.379247
H	1.944083	5.536754	-1.217092
C	3.476108	0.494398	-0.271788
O	1.194230	1.743829	1.090384
C	-1.664016	1.354496	-0.291646
C	-2.485302	2.561861	-0.470519
C	-3.149603	3.560999	-0.612367
H	-3.742241	4.436229	-0.743616
H	-1.403568	0.897904	-1.252759
H	-0.727528	1.613149	0.225642
C	-3.299792	-0.628939	2.248825
C	-2.342117	0.342139	1.885950
C	-1.553685	1.023784	2.813253
C	-1.777576	0.721397	4.153142
C	-2.740235	-0.231982	4.544207
C	-3.502779	-0.919456	3.604503
H	-0.753494	1.688254	2.491588
H	-1.174907	1.210745	4.913233
H	-2.875125	-0.444870	5.602271
H	-4.228206	-1.673347	3.898498
C	-3.574041	-0.880480	-1.307580
C	-2.523823	-1.074317	-2.288373
C	-4.917350	-1.091015	-1.638984
C	-2.992174	-1.427256	-3.606570
C	-5.321894	-1.439233	-2.923178
H	-5.652049	-0.972302	-0.845366
C	-4.328410	-1.600926	-3.905924
H	-2.224103	-1.567340	-4.363399
H	-6.373145	-1.579839	-3.157442
H	-4.615394	-1.872075	-4.922413
C	-3.267891	-0.588414	0.099320
N	-3.859936	-1.189300	1.122312
N	-2.349361	0.354220	0.511321
O	-1.286108	-0.963126	-2.029510
C	1.224592	-0.732867	-0.397363
C	0.544554	-1.245731	0.797671
C	-0.006736	-1.708968	1.765570
H	-0.545545	-2.034916	2.628142
H	1.018021	-1.394301	-1.246008
H	0.838126	0.250667	-0.656293

I-6a

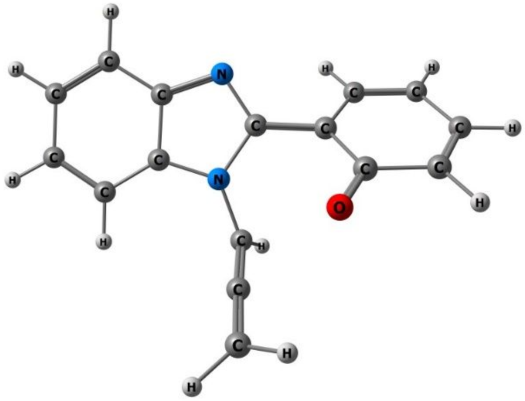

Atom type, (x,y,z) coordinates

C	3.799862	0.221872	-0.357206
C	2.374247	0.393025	-0.536850
C	1.575620	-0.703572	-0.010781
C	2.176919	-1.862676	0.500218
C	3.553919	-1.992548	0.614780
C	4.356023	-0.916709	0.184641
H	4.426185	1.037676	-0.710188
H	1.520971	-2.669884	0.820633
H	3.996430	-2.891746	1.031582
H	5.439967	-0.987850	0.274314
C	0.114376	-0.681518	-0.078328
C	-1.952611	-1.239202	-0.295868
N	-0.629613	0.419161	0.328647
C	-3.134400	0.784816	0.464886
C	-3.169345	-1.882659	-0.547204
C	-4.334235	0.121606	0.215396
C	-4.349881	-1.192201	-0.287431
H	-3.176241	-2.897845	-0.932438
H	-5.274681	0.629657	0.409181
H	-5.305087	-1.675141	-0.474612
O	1.892500	1.399602	-1.126668
C	-0.119355	1.554108	1.018024
C	-0.004586	2.710073	0.417017
C	0.175055	3.829281	-0.226677
H	-0.578247	4.610809	-0.254621
H	1.102081	3.940796	-0.784130
H	0.230728	1.393059	2.034715
C	-1.960120	0.085096	0.198427
N	-0.659470	-1.684974	-0.454996
H	-3.107445	1.801596	0.845088

TS7a

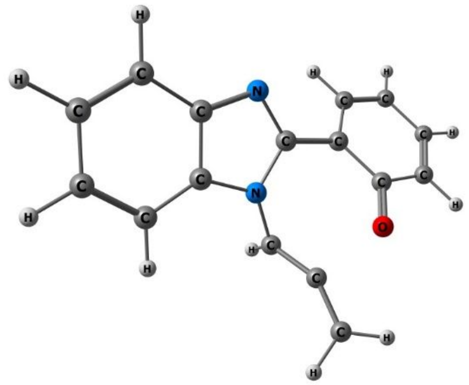

Atom type, (x,y,z) coordinates

C	3.694665	0.050391	-0.374086
C	2.290451	0.278241	-0.438375
C	1.457919	-0.781788	0.041363
C	2.020775	-1.985349	0.484402
C	3.395456	-2.180239	0.520553
C	4.229679	-1.140596	0.085142
H	4.334037	0.854103	-0.728538
H	1.340730	-2.772902	0.799320
H	3.811481	-3.116762	0.879280
H	5.310376	-1.269551	0.104633
C	-0.008207	-0.690871	-0.014177
C	-2.087712	-1.141535	-0.244360
N	-0.670417	0.468089	0.317018
C	-3.144586	0.996113	0.377317
C	-3.340085	-1.721871	-0.476909
C	-4.379202	0.398026	0.143581
C	-4.475206	-0.942469	-0.279384
H	-3.408451	-2.755703	-0.802564
H	-5.288166	0.975360	0.288828
H	-5.457673	-1.373386	-0.452351
O	1.834109	1.383664	-0.948340
C	-0.078571	1.630202	0.900688
C	0.684114	2.454618	0.166300
C	1.065437	3.689847	-0.128810
H	0.349762	4.499806	-0.033795
H	2.059206	3.893943	-0.507563
H	-0.102513	1.693278	1.983233
C	-2.012854	0.208402	0.171975
N	-0.821340	-1.675607	-0.353557
H	-3.051800	2.028392	0.700702

I-7

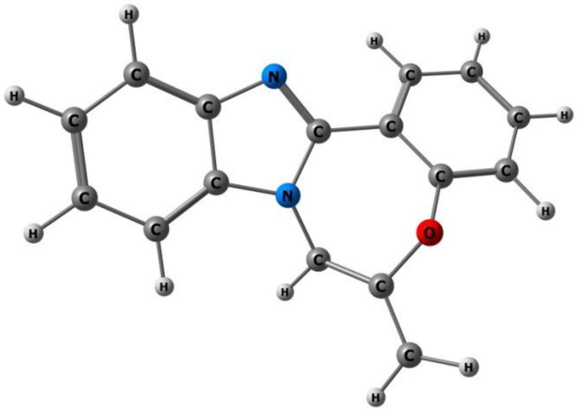

Atom type, (x,y,z) coordinates

C	-3.612590	0.043425	0.217419
C	-2.229087	0.201120	0.319676
C	-1.378652	-0.874021	0.016754
C	-1.938794	-2.103680	-0.347785
C	-3.317238	-2.262625	-0.435751
C	-4.156435	-1.179635	-0.161609
H	-4.236687	0.898018	0.459586
H	-1.260236	-2.925095	-0.557690
H	-3.736441	-3.222306	-0.723782
H	-5.234888	-1.291051	-0.236552
C	0.072166	-0.717494	0.064059
C	2.161974	-1.101025	0.193064
N	0.652354	0.493061	-0.213729
C	3.100551	1.127103	-0.302760
C	3.451957	-1.635969	0.332339
C	4.366507	0.574752	-0.159820
C	4.539262	-0.793349	0.150383
H	3.580809	-2.686655	0.576269
H	5.241946	1.206819	-0.280660
H	5.546390	-1.189006	0.251248
O	-1.767501	1.369072	0.864082
C	0.023286	1.682469	-0.673970
C	-1.051316	2.254839	0.021211
C	-1.535849	3.533913	0.012987
H	-0.991172	4.322598	-0.491913
H	-2.495409	3.751305	0.463558
H	0.625544	2.279752	-1.344153
C	2.008818	0.273048	-0.125627
N	0.935967	-1.694973	0.324501
H	2.954528	2.179200	-0.527399

I-7 + H_2O

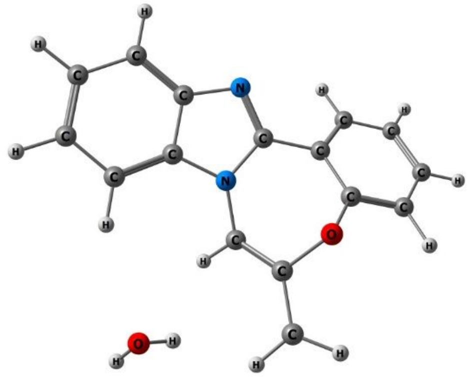

Atom type, (x,y,z) coordinates

C	-3.478721	-0.529493	-0.011135
C	-2.120730	-0.219358	0.063967
C	-1.161563	-1.241259	0.011995
C	-1.589207	-2.569902	-0.084475
C	-2.943081	-2.880251	-0.148894
C	-3.890162	-1.854321	-0.119701
H	-4.188714	0.290275	0.034978
H	-0.829443	-3.345482	-0.103634
H	-3.260074	-3.916051	-0.225302
H	-4.950111	-2.086491	-0.175046
C	0.267970	-0.937119	0.062685
C	2.373256	-1.072573	0.349191
N	0.754828	0.239009	-0.446576
C	3.137093	1.078943	-0.585809
C	3.693654	-1.436747	0.651816
C	4.435556	0.695843	-0.278753
H	2.911593	2.039427	-1.038538

C	4.710618	-0.549059	0.330132
H	3.898667	-2.392360	1.125500
H	5.257708	1.369590	-0.502737
H	5.741300	-0.810633	0.553722
O	-1.783362	1.084894	0.320277
C	-1.123049	1.806516	-0.714602
C	0.052865	1.234555	-1.181041
C	-1.756938	2.979670	-1.063194
O	-1.686655	3.502476	2.044957
H	-1.597016	3.647166	1.083875
H	-1.724925	2.538727	2.082607
C	2.118058	0.178676	-0.264634
N	1.196106	-1.744746	0.558031
H	-2.814718	3.096371	-0.855594
H	-1.281185	3.656725	-1.763134
H	0.586813	1.649205	-2.024043

TS8

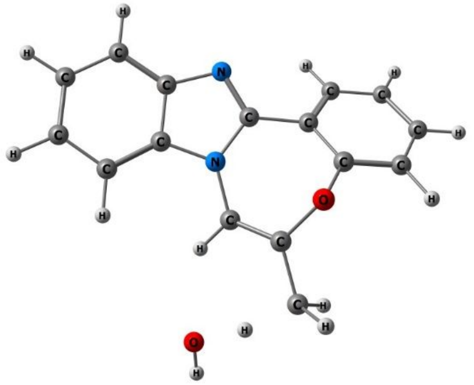

Atom type, (x,y,z) coordinates

C	-3.659532	-0.282526	0.401943
C	-2.272798	-0.136999	0.476660
C	-1.432158	-1.133484	-0.046121
C	-2.004724	-2.275438	-0.617831
C	-3.385308	-2.423874	-0.683418
C	-4.213511	-1.420757	-0.174264
H	-4.273541	0.511383	0.814011
H	-1.335240	-3.040067	-0.999389
H	-3.814852	-3.314496	-1.131594
H	-5.293617	-1.526882	-0.225501
C	0.028383	-1.021854	0.025833
C	2.110185	-1.503342	0.064511
N	0.676166	0.191468	0.011392
C	3.145481	0.737129	0.024457
C	3.369650	-2.117040	0.098813
C	4.381959	0.105804	0.052421
H	3.045437	1.818360	0.000797

C	4.492474	-1.300961	0.091096
H	3.449491	-3.199385	0.129313
H	5.285189	0.708531	0.048315
H	5.480988	-1.750966	0.116240
O	-1.766846	0.945783	1.131347
C	-1.053697	1.875685	0.307498
C	0.138195	1.491027	-0.161568
C	-1.630006	3.205089	0.070280
O	0.573832	4.381406	-0.745437
H	-0.552183	3.933991	-0.352025
H	0.407007	5.139487	-1.315737
C	2.023983	-0.095160	0.032454
N	0.848478	-2.055981	0.058641
H	-2.109138	3.568782	0.988417
H	-2.393996	3.155384	-0.720069
H	0.744603	2.210167	-0.706881

11a + OH$^-$

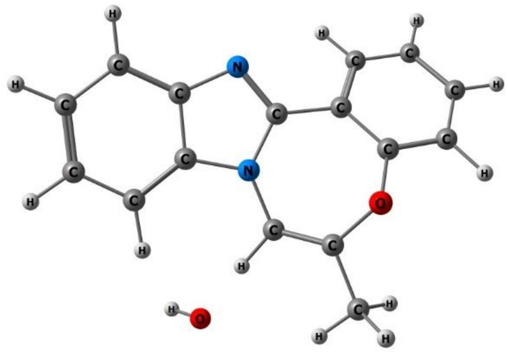

Atom type, (x,y,z) coordinates

C	3.799327	0.071706	-0.235370
C	2.411816	0.143947	-0.368375
C	1.606567	-0.934912	0.026633
C	2.228359	-2.078371	0.544701
C	3.610405	-2.154553	0.671001
C	4.399904	-1.072093	0.279984
H	4.382972	0.927211	-0.559606
H	1.589469	-2.906790	0.833492
H	4.068796	-3.051937	1.074907
H	5.481124	-1.117753	0.375002
C	0.141461	-0.932233	-0.096515
C	-1.888557	-1.601399	-0.161819
N	-0.614972	0.213314	-0.037527
C	-3.121870	0.540061	0.020615
C	-3.086003	-2.329658	-0.223513
C	-4.289793	-0.209077	-0.036359
H	-3.090943	1.626793	0.165764
C	-4.274890	-1.617674	-0.163570
H	-3.067266	-3.411947	-0.309626
H	-5.246250	0.302465	0.027127
H	-5.219935	-2.153079	-0.206072
O	1.905314	1.255471	-0.983188
C	0.946345	2.025999	-0.292517
C	-0.228218	1.563080	0.126615
C	1.334102	3.461973	-0.166592
O	-2.087315	3.372277	0.853543
H	0.484319	4.009000	0.254074
H	-2.274521	3.675695	1.748610
C	-1.935615	-0.197380	-0.059050
N	-0.578283	-2.034939	-0.171105
H	1.580176	3.873078	-1.152911
H	2.216501	3.583026	0.474515
H	-1.015698	2.297139	0.577084

TS2a

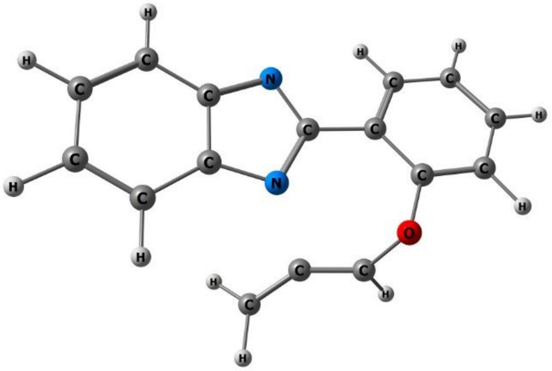

Atom type, (x,y,z) coordinates

C	3.770224	0.309501	-0.451967
C	2.380933	0.438210	-0.405182
C	1.575321	-0.628237	0.034016
C	2.217929	-1.807235	0.440339
C	3.601802	-1.934016	0.405262
C	4.385316	-0.872099	-0.050868
H	4.345040	1.159245	-0.809558
H	1.586981	-2.627899	0.768057
H	4.068650	-2.861724	0.726326
H	5.468306	-0.961529	-0.089601
C	0.102856	-0.592780	0.014335
C	-1.905612	-1.280436	0.045216
N	-0.612325	0.542132	-0.214220
C	-3.123846	0.811993	-0.397927
C	-3.108581	-2.002493	0.094985
C	-4.302634	0.080525	-0.336393
C	-4.297251	-1.311398	-0.091071
H	-3.095067	-3.075946	0.268611
H	-5.253444	0.586766	-0.488068
H	-5.243216	-1.847689	-0.057019
O	1.837348	1.617518	-0.847830
C	1.213226	2.336476	0.181476
C	-0.096600	2.283322	0.427537
C	-1.105291	2.975686	0.987800
H	-1.826179	2.504267	1.648948
H	-1.285227	4.002137	0.684217
H	1.891628	2.907762	0.807850
C	-1.916595	0.122847	-0.195787
N	-0.612787	-1.711060	0.173157
H	-3.127661	1.877757	-0.600288

TS4

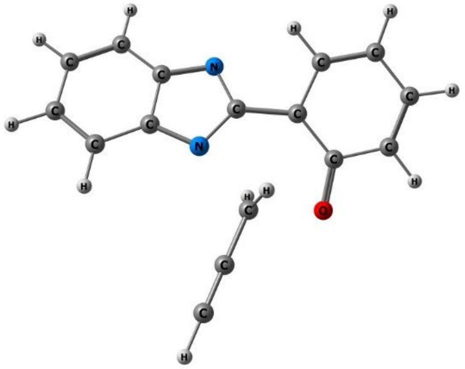

Atom type, (x,y,z) coordinates

C	-2.003443	-1.296573	0.125495
C	-2.071558	0.097498	-0.149885
C	-3.300107	0.761368	-0.262827
C	-4.456926	0.009207	-0.094348
C	-4.399001	-1.375368	0.175648
C	-3.182559	-2.038600	0.286169
H	-3.339576	1.826554	-0.474076
H	-5.426762	0.493826	-0.173744
H	-5.326521	-1.928850	0.298117
H	-3.133854	-3.104262	0.493419
N	-0.692161	-1.682830	0.196103
N	-0.784875	0.533013	-0.271957
C	1.533374	-0.615427	-0.032565
C	1.980549	-1.937707	-0.259803
C	2.575730	0.394987	0.185923
C	3.305635	-2.337684	-0.286177
H	1.206118	-2.680733	-0.410186
C	3.926638	-0.062779	0.138939
C	4.295522	-1.373021	-0.076857
H	3.557632	-3.377664	-0.470267
H	4.675113	0.707759	0.296562
H	5.350046	-1.641577	-0.089661
C	0.003785	-0.562303	-0.032599
O	2.460625	1.685961	0.418118
C	0.572169	1.914899	0.034052
C	0.138091	3.287484	-0.037696
C	-0.221244	4.439095	-0.091182
H	-0.537553	5.456182	-0.137446
H	0.537849	1.481693	1.011867
H	0.911838	1.497649	-0.892273

TS7p

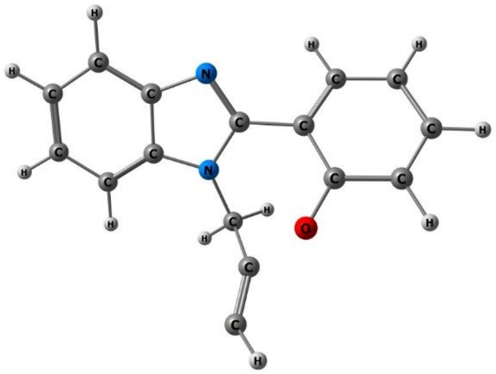

Atom type, (x,y,z) coordinates

C	3.690452	0.252826	-0.256904
C	2.278417	0.383090	-0.344782
C	1.508910	-0.767705	0.003173
C	2.138450	-1.970450	0.347506
C	3.521494	-2.074487	0.399354
C	4.292734	-0.942117	0.097511
H	4.277900	1.129346	-0.512853
H	1.504631	-2.824528	0.571608
H	3.994901	-3.012170	0.673200
H	5.378439	-1.001578	0.137685
C	0.047547	-0.721162	-0.034321
C	-2.027122	-1.185805	-0.254265
N	-0.622868	0.408985	0.360155
C	-3.103545	0.922854	0.436370
C	-3.275514	-1.771249	-0.499940
C	-4.331191	0.318516	0.188358
C	-4.415789	-1.010588	-0.272305
H	-3.334491	-2.793552	-0.861035
H	-5.243967	0.886268	0.343785
H	-5.394781	-1.444160	-0.456795
O	1.766289	1.498824	-0.807545
C	-0.009138	1.580029	0.973615
C	0.588698	2.524352	-0.003039
C	0.453047	3.750555	-0.333863
H	0.746179	1.211188	1.678715
C	-1.964137	0.148765	0.213010
N	-0.758679	-1.702911	-0.399934
H	-3.032737	1.955391	0.764549
H	-0.777666	2.109199	1.538843
H	1.062238	4.226956	-1.095748

(*Z*)-9a

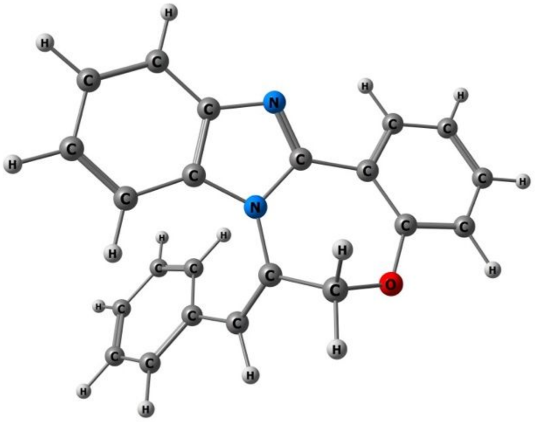

Atom type, (x,y,z) coordinates

C	0.183166	2.461624	-0.000654
C	0.814327	1.337642	-0.562998
C	2.130068	1.370119	-1.027732
C	2.805515	2.576236	-0.892312
C	2.193529	3.707251	-0.316843
C	0.881540	3.667241	0.130648
H	2.608175	0.498913	-1.461946
H	3.832683	2.645897	-1.235418
H	2.762271	4.627456	-0.229329
H	0.394454	4.534563	0.563527
N	-1.123901	2.167091	0.326468
N	-0.154986	0.339887	-0.543193
C	-2.585801	0.209686	0.210823
C	-3.474711	0.878413	1.080289
C	-3.004788	-1.029116	-0.312324
C	-4.698063	0.348192	1.442093
H	-3.150783	1.837513	1.467693
C	-4.242585	-1.568534	0.066003
C	-5.082228	-0.897021	0.934749
H	-5.345870	0.894473	2.118987
H	-4.514856	-2.527385	-0.362041
H	-6.036068	-1.336389	1.209425
C	-1.306148	0.916082	-0.019225
O	-2.323799	-1.828772	-1.183255
C	-1.272120	-1.286989	-1.964748
C	-0.076241	-0.943297	-1.122268
C	0.939575	-1.802609	-0.940836
H	-1.021178	-2.072781	-2.678627
H	-1.628821	-0.400372	-2.505539
C	2.088463	-1.710556	-0.025510
C	3.274144	-2.372857	-0.371307
C	2.032243	-1.023202	1.195887
C	4.388123	-2.324680	0.461759
C	3.142007	-0.985068	2.033031
H	1.115169	-0.523892	1.492953
C	4.324998	-1.627238	1.666731
H	3.082904	-0.450113	2.975545
H	5.190383	-1.590397	2.320852
H	5.301124	-2.836051	0.173347
H	3.321091	-2.924560	-1.307005
H	0.901841	-2.703455	-1.550836

(*E*)-9a

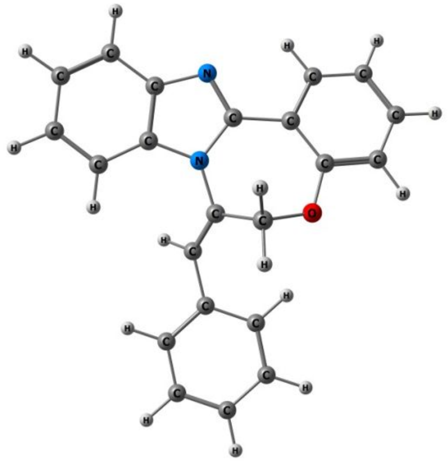

Atom type, (x,y,z) coordinates

C	2.665154	1.853514	-0.054667
C	1.267047	1.982416	0.052828
C	0.635538	3.225015	0.138501
C	1.455331	4.345414	0.086690
C	2.853596	4.233461	-0.042860
C	3.473592	2.994967	-0.108604
H	-0.438840	3.319496	0.252190
H	1.005812	5.331169	0.150207
H	3.453479	5.136868	-0.082492
H	4.550650	2.895018	-0.191814
N	3.025208	0.524097	-0.065216
N	0.795634	0.677262	0.101561
C	1.871860	-1.633328	-0.009703
C	3.089061	-2.231699	-0.402446
C	0.790161	-2.496794	0.255492
C	3.231804	-3.597830	-0.554231
H	3.921364	-1.565237	-0.596902
C	0.935687	-3.881091	0.090299
C	2.136993	-4.433033	-0.313504
H	4.185070	-4.010290	-0.866384
H	0.070296	-4.497856	0.308007
H	2.223969	-5.508548	-0.431298
C	1.905906	-0.152236	0.036338
O	-0.462555	-2.144354	0.672197
C	-0.654159	-0.875020	1.273935
C	-0.535970	0.243031	0.278770
C	-1.549125	0.820521	-0.383718
H	-1.655860	-0.903549	1.703417
H	0.081383	-0.732047	2.075810
C	-2.974283	0.456129	-0.283570
C	-3.943026	1.469430	-0.308456
C	-3.396598	-0.879698	-0.217162
C	-5.297046	1.161118	-0.227873
C	-4.753353	-1.186209	-0.139951
H	-2.660771	-1.678136	-0.260842
C	-5.705935	-0.169386	-0.138126
H	-5.066145	-2.224779	-0.095107
H	-6.762469	-0.411663	-0.081547
H	-6.033695	1.958416	-0.239395
H	-3.626299	2.506798	-0.382453
H	-1.306797	1.637484	-1.061286

(*Z*)-9b

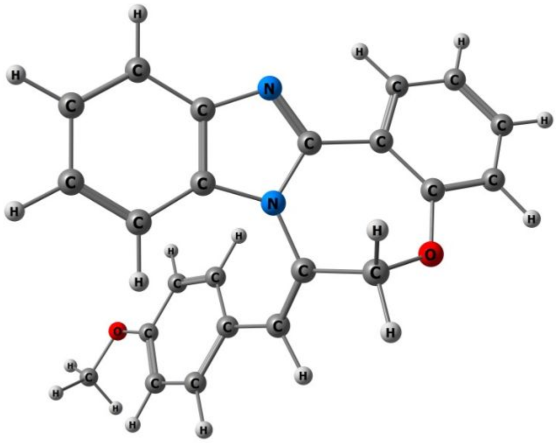

Atom type, (x,y,z) coordinates

C	0.708921	2.559515	-0.153908
C	0.018893	1.608783	0.619682
C	-1.200401	1.883224	1.240314
C	-1.719786	3.156948	1.047923
C	-1.049616	4.118291	0.265704
C	0.166033	3.836808	-0.339676
H	-1.722019	1.139143	1.832488
H	-2.668208	3.414900	1.508112
H	-1.496125	5.099484	0.139963
H	0.696412	4.573830	-0.933305
N	1.904391	2.037356	-0.603864
N	0.830381	0.480967	0.603315
C	3.086223	-0.100308	-0.417206
C	3.916358	0.303502	-1.485731
C	3.409342	-1.309233	0.230427
C	4.990557	-0.451394	-1.917022
H	3.670622	1.241667	-1.970372
C	4.494825	-2.076105	-0.217351
C	5.277715	-1.661713	-1.278061
H	5.596528	-0.106919	-2.747913
H	4.698480	-3.000036	0.313007
H	6.113716	-2.273687	-1.601649
C	1.961743	0.816792	-0.127660
O	2.771942	-1.878848	1.293050
C	1.897252	-1.107622	2.100189
C	0.660647	-0.703919	1.351154
C	-0.472558	-1.424199	1.387418
H	1.643077	-1.760236	2.936471
H	2.424561	-0.220209	2.474967
C	-1.698723	-1.268805	0.593865
C	-2.908857	-1.728669	1.118542
C	-1.710398	-0.708330	-0.697048
C	-4.107776	-1.610877	0.415796
C	-2.887821	-0.596426	-1.412223
H	-0.783800	-0.364027	-1.145523
C	-4.097276	-1.036936	-0.856742
H	-2.903377	-0.167335	-2.408337
H	-5.025569	-1.972468	0.863344
H	-2.921621	-2.184594	2.105669
H	-0.478027	-2.246526	2.101191
O	-5.197989	-0.872828	-1.632034
C	-6.444706	-1.276642	-1.102445
H	-6.457626	-2.353679	-0.897510
H	-6.680043	-0.725886	-0.184097
H	-7.185556	-1.044368	-1.866439

(*E*)-9b

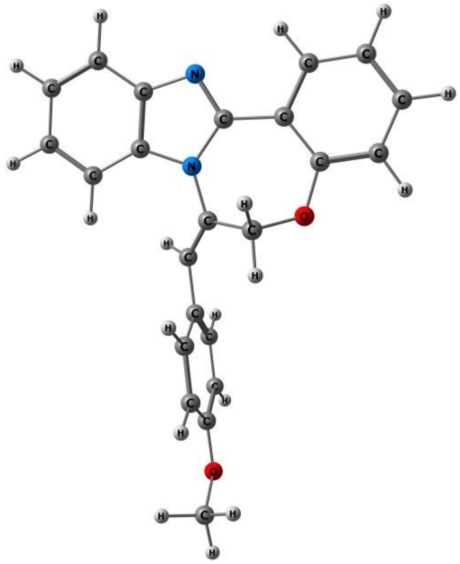

Atom type, (x,y,z) coordinates

C	-3.147019	2.003452	-0.029076
C	-1.740459	1.999020	-0.023380
C	-0.994579	3.179894	-0.071317
C	-1.709404	4.370726	-0.095279
C	-3.117614	4.389855	-0.073829
C	-3.850419	3.213515	-0.046514
H	0.088966	3.179140	-0.098182
H	-1.166091	5.309485	-0.131440
H	-3.633610	5.344470	-0.087625
H	-4.935210	3.210998	-0.048162
N	-3.629348	0.715002	-0.045403
N	-1.386241	0.651803	-0.027403
C	-2.708979	-1.540730	-0.035953
C	-4.028689	-2.020092	0.105972
C	-1.680497	-2.496730	-0.104131
C	-4.317198	-3.368234	0.208717
H	-4.819840	-1.280642	0.149116
C	-1.973543	-3.861694	0.012755
C	-3.275641	-4.300100	0.172812
H	-5.345438	-3.693487	0.324048
H	-1.139951	-4.553812	-0.038274
H	-3.480470	-5.362578	0.258733
C	-2.576632	-0.067498	-0.054901
O	-0.350435	-2.236718	-0.255245
C	0.056120	-1.081103	-0.972902
C	-0.069647	0.145710	-0.115947
C	0.925070	0.719878	0.577973
H	1.097066	-1.266652	-1.229468
H	-0.540070	-0.981134	-1.889623
C	2.363665	0.414096	0.454542
C	3.136975	0.196717	1.607341
C	3.011364	0.387919	-0.781476
C	4.491476	-0.073979	1.520370
C	4.376836	0.119014	-0.887892
H	2.447028	0.609761	-1.683694
C	5.120155	-0.119487	0.269212
H	4.841065	0.114062	-1.866623
H	5.090601	-0.254467	2.406611
H	2.659578	0.229153	2.582964
H	0.652244	1.483992	1.304338
O	6.450378	-0.389502	0.285383
C	7.130058	-0.429403	-0.953194
H	8.168549	-0.659782	-0.719026
H	6.720076	-1.210270	-1.604653
H	7.076693	0.539417	-1.463928
